# Supplementary material for: Vaginal ring acceptability and related preferences among women in low- and middle-income countries: A systematic review and narrative synthesis
Source: PLoS One. 2019 Nov 8;14(11):e0224898. doi: 10.1371/journal.pone.0224898 (PMC6839883; doi:10.1371/journal.pone.0224898)
Supplement: S1 File — (DOCX) [file pone.0224898.s001.docx]

**S1 File. Search strategy for PubMed, Embase, and Web of Science**

**PubMed**

#1 "vaginal ring"[tw] OR "intravaginal ring"[tw] OR "v-ring"[tw] OR "microbicide ring"[tw] OR "contraceptive ring"[tw] OR "vaginal rings"[tw] OR "intravaginal rings"[tw] OR "v-rings"[tw] OR "microbicide rings"[tw] OR "contraceptive rings"[tw] Filters: Publication date from 1970/01/01; English 1078

#2 opinion*[Title/Abstract] OR acceptability[Title/Abstract] OR acceptable[Title/Abstract] OR acceptance[Title/Abstract] OR values[Title/Abstract] OR preference*[Title/Abstract] OR prefer*[Title/Abstract] OR benefit*[Title/Abstract] OR harm*[Title/Abstract] OR unintended effect*[Title/Abstract] OR unintended consequence*[Title/Abstract] OR satisfaction[Title/Abstract] OR satisfy*[Title/Abstract] OR attitude*[Title/Abstract] OR choice*[Title/Abstract] OR adherence[Title/Abstract] OR non-adherence[Title/Abstract] OR nonadherence[Title/Abstract] OR self-efficacy[Title/Abstract] OR knowledge*[Title/Abstract] OR understand*[Title/Abstract] OR belief*[Title/Abstract] OR believe[Title/Abstract] OR perception*[Title/Abstract] OR preceiv*[Title/Abstract] OR "Patient Acceptance of Health Care"[Mesh:NoExp] OR "Social Values"[Mesh] OR "Patient Preference"[Mesh] OR "Patient Harm"[Mesh] OR "Patient Satisfaction"[Mesh] OR "Attitude to Health"[Mesh:NoExp] OR "Attitude"[Mesh:NoExp] OR "Choice Behavior"[Mesh:NoExp] OR "Treatment Adherence and Compliance"[Mesh:NoExp] OR "Self Efficacy"[Mesh] OR "Contraception Behavior"[Mesh] Filters: Publication date from 1970/01/01; English 4096861

#3 (#1 AND #2) NOT (("Animals"[Mesh] NOT "Humans"[Mesh]) OR "Comment"[Publication Type] OR "Letter"[Publication Type] OR "Editorial"[Publication Type]) Filters: Publication date from 1970/01/01; English 517

**Embase**

#1 'vagina ring'/exp/mj OR 'vaginal ring':ti,ab OR 'intravaginal ring':ti,ab OR 'v-ring':ti,ab OR 'microbicide ring':ti,ab OR 'contraceptive ring':ti,ab OR 'vaginal rings':ti,ab OR 'intravaginal rings':ti,ab OR 'v-rings':ti,ab OR 'microbicide rings':ti,ab OR 'contraceptive rings':ti,ab AND [english]/lim AND [embase]/lim AND [1970-2019]/py 1,541

#2 opinion*:ti,ab OR acceptability:ti,ab OR acceptable:ti,ab OR acceptance:ti,ab OR values:ti,ab OR preference*:ti,ab OR prefer*:ti,ab OR benefit*:ti,ab OR harm*:ti,ab OR 'unintended effect*':ti,ab OR 'unintended consequence*':ti,ab OR satisfaction:ti,ab OR satisfy*:ti,ab OR attitude*:ti,ab OR choice*:ti,ab OR adherence:ti,ab OR 'non-adherence':ti,ab OR nonadherence:ti,ab OR 'self-efficacy':ti,ab OR knowledge*:ti,ab OR understand*:ti,ab OR belief*:ti,ab OR believe:ti,ab OR perception*:ti,ab OR preceiv*:ti,ab OR 'patient attitude'/de OR 'social psychology'/de OR 'patient preference'/exp OR 'patient harm'/exp OR 'patient satisfaction'/exp OR 'attitude to health'/exp OR 'attitude'/de OR 'patient decision making'/exp OR 'patient compliance'/de OR 'self concept'/de OR 'contraceptive behavior'/de AND [english]/lim AND [embase]/lim AND [1970-2019]/py 4,226,421

#3 (#1 AND #2) NOT (('animal'/exp NOT 'human'/exp) OR comment*:ti OR letter:it OR editorial:it OR 'letter'/exp OR 'editorial'/exp OR [letter]/lim OR [editorial]/lim) AND [english]/lim AND [embase]/lim AND [1970-2019]/py 791

**Web of Science**

#6 566 (#4 NOT #5) AND LANGUAGE: (English) Indexes=SCI-EXPANDED, SSCI, CPCI-S, CPCI-SSH Timespan=1970-2019

#5 1,711,611 TOPIC: ("noetherian" OR "die roll" OR "single-digit replantation" OR "dermatoglyphics" OR "fine blanking" OR "mice" OR "mouse" OR "supramolecular" OR "explosive RDX" OR "higher plants" OR "hyperstructure" OR "wafer edge" OR "von Neumann regular ring" OR "fuzzy logic" OR "solar winds" OR "cotorsion module" OR "bisquaternary" OR "catalytic activity" OR "H-v-modules" OR "gas rings" OR "ball bearing" OR "H-v-rings" OR "centrifugal pump") AND LANGUAGE: (English) Indexes=SCI-EXPANDED, SSCI, CPCI-S, CPCI-SSH Timespan=1970-2019

#4 588 #2 AND #1 Refined by: [excluding] DOCUMENT TYPES: ( EDITORIAL MATERIAL OR LETTER ) Indexes=SCI-EXPANDED, SSCI, CPCI-S, CPCI-SSH Timespan=1970-2019

#3 598 #2 AND #1 Indexes=SCI-EXPANDED, SSCI, CPCI-S, CPCI-SSH Timespan=1970-2019

#2 7,554,633 TOPIC: (opinion* OR "acceptability" OR "acceptable" OR "acceptance" OR "values" OR preference* OR prefer* OR benefit* OR harm* OR "unintended effect*" OR "unintended consequence*" OR "satisfaction" OR satisfy* OR attitude* OR choice* OR "adherence" OR "non-adherence" OR "nonadherence" OR "self-efficacy" OR knowledge* OR understand* OR belief* OR "believe" OR perception* OR preceiv*) AND LANGUAGE: (English) Indexes=SCI-EXPANDED, SSCI, CPCI-S, CPCI-SSH Timespan=1970-2019

#1 1,509 TOPIC: ("vaginal ring" OR "intravaginal ring" OR "v-ring" OR "microbicide ring" OR "contraceptive ring" OR "vaginal rings" OR "intravaginal rings" OR "v-rings" OR "microbicide rings" OR "contraceptive rings") AND LANGUAGE: (English) Indexes=SCI-EXPANDED, SSCI, CPCI-S, CPCI-SSH Timespan=1970-2019
