# Supplementary material for: Vaginal ring acceptability and related preferences among women in low- and middle-income countries: A systematic review and narrative synthesis
Source: PLoS One. 2019 Nov 8;14(11):e0224898. doi: 10.1371/journal.pone.0224898 (PMC6839883; doi:10.1371/journal.pone.0224898)
Supplement: S2 File — (DOCX) [file pone.0224898.s002.docx]

**S2 File. Study characteristics, acceptability outcomes, and risk of bias assessment for studies in the peer-reviewed literature**

Table 1a. Study characteristics and risk of bias of randomized controlled trials addressing vaginal ring acceptability, by indication

| **Author;**  **Year;**  **Country;**  **Funding;**  **Trial or study name** | **Study design;**  **Setting;**  **Intervention (N group)** | **Timing of acceptability assessment** (prospective / concurrent / retrospective) | **Key Inclusion and Exclusion Criteria** | **Sample characteristics** | **Risk of bias** |
| --- | --- | --- | --- | --- | --- |
| HIV prevention | | | | | |
| Baeten 2016, Palanee-Phillips 2018, Mensch 2019;  Malawi, South Africa, Uganda, Zimbabwe;  National Institutes of Health;  MTN 020/ASPIRE | Phase III randomized placebo-controlled trial;  15 research sites in Malawi (Blantyre, Lilongwe), South Africa (Cape Town, Durban [7 sites], Johannesburg), Uganda (Kampala), Zimbabwe (Chitungwiza [2 sites], Harare);  G1*: Dapivirine vaginal ring (N=1,313)  G2*: Placebo vaginal ring (N=1,316) | Prospective, concurrent | Inclusion: Women age 18 through 45 years at screening, able and willing to provide written informed consent able and willing to provide adequate locator information, HIV uninfected based on testing performed at screening and enrollment, per participant report, sexually active, defined as having vaginal intercourse at least once in the 3 months prior to screening, using an effective method of contraception at enrollment, and intending to use an effective method for the duration of study participation, and, agrees not to participate in other research studies involving drugs, medical devices, vaginal products, or vaccines for the duration of study participation.  Exclusion: Women who intend to become pregnant during study, plan to relocate or travel away from the study site, pregnant, currently breastfeeding, diagnosed with a UTI, pelvic inflammatory disease, an STI or reproductive tract infection, has a clinically apparent Grade 2 or higher pelvic exam finding, has laboratory abnormalities or other clinical contraindications. | Mean age (SD):  G1*: 27.2 (6.1)  G2*: 27.3 (6.3)  N (%) married: 1,074 (41%)  N (%) nulliparous: NR  N (%) rural: NR | Low |
| Hardy;  2007;  Brazil;  CONRAD, FAPESP, and FAEPEX/Unicamp;  NR | Randomized cross-over trial;  Communities in Campinas and Campo Grande;  G1: Single size diaphragm (65mm) (N=405)  G2*: Vaginal ring (N=405)  G3: Gel with disposable applicator (N=405) | Concurrent | Inclusion: Age 18 to 49 years; living in Campinas or Campo Grande; having a stable sexual partner; using a nonhormonal contraceptive method (condom, copper IUD, tubal ligation or vasectomy); having had regular menstrual cycles during the previous 3 months; male partner agreed to participate.  Exclusion: NR | Mean age (SD): NR  N (%) married: NR  N (%) nulliparous: NR  N (%) rural: NR | High |
| Nel;  2016;  Kenya, Malawi, South Africa, Tanzania;  International Partnership for Microbicides;  NR | Phase I/II double-blind randomized placebo-controlled trial;  10 research centers in Kenya (Kisumu), Malawi (Lilongwe), South Africa (Edendale, Ladysmith, Pinetown, Brits, Cape Town [2 sites], Paarl), and Tanzania (Moshi);  G1*: Dapivirine vaginal ring (N=140)  G2*: Placebo ring (N=140) | Concurrent | Inclusion: Generally healthy, HIV negative, sexually active, tested negative for pregnancy, willing to use a stable form of contraception during the trial (oral contraceptives, transdermal patches, long-acting injectable progestins, intrauterine devices or had a surgical sterilization), willing to refrain from using vaginal products or objects (including tampons), had a normal appearing cervix and vagina based on pelvic examination and colposcopy, and normal Pap test results.  Exclusion: NR | Mean age (SD):  G1*: 25.8 (5.6)  G2*: 25.4 (5.4)  N (%) married:  G1*: 31 (22%)  G2*: 30 (21%)  N (%) nulliparous: NR  N (%) rural: NR | Unclear |
| van der Straten 2012, Montgomery 2012, Nel 2018;  Tanzania, South Africa;  NIH;  IPM 011 | Randomized cross-over trial;  3 research centers in  South Africa (Johannesburg, Durban and Cape Town), and 1 research center in Moshi, Tanzania;  G1*: Placebo vaginal ring (N=170)  G2: No treatment (N=170) | Prospective, concurrent | Inclusion: Women 18 to 35, HIV-negative, healthy, sexually active (at least one vaginal sexual act per month in the past 3 months), not pregnant or breastfeeding, regular menstrual cycle, normal appearing cervix and vagina, normal Pap test results, no recent gynecological surgery, asymptomatic for genital and STIs, and using an effective contraceptive method.  Exclusion: NR | Mean age (SD)^[[1]](#endnote-2)^: 27 (NR)  N (%) married: 47 (29.9%)  N (%) nulliparous: NR  N (%) rural: NR | Low |
| Contraception | | | | | |
| Fan;  2016;  China;  Merck;  NR | Phase III open-label, randomized, comparative-group multicenter trial;  29 obstetrics and gynecology centers;  G1*: Contraceptive vaginal ring (NuvaRing) (N=720)  G2: COC^[[2]](#endnote-3)^ (N=240) | NA | Inclusion: Healthy Chinese women of reproductive age (18 to 40 years), seeking contraception.  Exclusion: Contraindications to contraceptive steroids; previous use of an injectable hormonal method of contraception more recently than the pre-specified washout period or before spontaneous menstruation had occurred following a delivery or abortion; breastfeeding within 2 months of the start of trial medication; undiagnosed vaginal bleeding; an abnormal cervical smear diagnosed during screening; and use of drugs that interfered with the metabolism of contraceptive hormones. There was an additional exclusion of conditions that predisposed to hyperkalaemia (renal insufficiency, hepatic dysfunction and adrenal insufficiency), in relation to the antimineralocorticoid activity of DRSP^3^^[[3]](#endnote-4)^. | Mean age (SD):  G1*: 31.8 (4.0)  G2: 31.2 (3.9)  N (%) married: NR  N (%) nulliparous: NR  N (%) rural: NR | Low |
| Kestelyn 2018a, Kestelyn 2018b;  Rwanda;  European and Developing Countries Clinical Trials Partnership;  NR | Open-label single-center randomized clinical trial;  Rinda Ubuzima research site in Kigali;  G1*: NuvaRing, intermittent use (N=60)  G2*: NuvaRing, continuous use (N=60) | Prospective, concurrent, retrospective | Inclusion: Women between 18 and 35 years old, willing to provide informed consent, HIV negative, not pregnant, sexually active, in good physical and mental health, and not currently using a modern contraceptive method with the exception of barrier methods but interested in and eligible for NuvaRing use.  Exclusion: Used hormonal contraception in the three months prior to screening; were currently smoking, breastfeeding, or using antimicrobial medication; or had a (history of a) condition contraindicating NuvaRing use (hysterectomy, recent genital tract surgery, significant urogenital or uterine prolapse, undiagnosed vaginal bleeding, incontinence, chronic and/or recurrent vulvovaginal candidiasis, urethral obstruction, cardiovascular disease, venous thrombosis, migraine with focal neurological symptoms, diabetes mellitus with vascular involvement, pancreatitis, severe hepatic disease, or known/suspected hypersensitivity to any of the NuvaRing excipients). | Mean age in years (SD): 28 (NR)  Married N (%):  G1*: 37 (62%)  G2*: 36 (60%)  Nulliparous N (%): NR  Rural N (%): NR | Low |
| Mohamed;  2011;  Egypt;  NR;  NR | Randomized open-label trial;  Contraception clinic at Kasr El-Aini Hospital;  G1*: NuvaRing (N=300)  G2: COC (N=300) | Concurrent | Inclusion: Women age 17 to 42 years who attended the contraception clinic at Kasr El-Aini Hospital in Cairo, Egypt, between May 1, 2008, and July 31, 2010, had regular menstrual cycles, were at risk of becoming pregnant, and sought contraception.  Exclusion: Contraindications for contraceptive steroid use, the use of an injectable hormonal contraceptive 6 months prior, use of a hormone medicated intrauterine device or any other hormonal contraceptive within 2 months, abortion or breastfeeding within 2 months, an abnormal cervical smear, or a prolapse of the uterine cervix, cystocele, and/or rectocele. | Mean age (SD):  G1*: 29.7 (4.1)  G2: 30.9 (4.2)  N (%) married: NR  N (%) nulliparous: NR  N (%) rural: NR | High |
| Sharma;  2018;  India;  NR;  NR | Randomized controlled trial;  Department of Obstetrics and Gynecology, Swami Dayanand Hospital;  G1*: NuvaRing (N=225)  G2: COC (N=225) | Concurrent | Inclusion: Women attending gynecology outpatient department, aged 18 to 40 years, at risk of pregnancy, seeking contraception and willing to participate.  Exclusion: Contraindications for contraceptive steroids i.e. current or past history of ischemic heart disease, stroke, valvular heart disease, migraine, known or suspected breast carcinoma, diabetes with vascular disease, thrombophlebitis or thromboembolic disorder, history of deep vein thrombosis, hypertension, symptomatic gall bladder disease, past history of COC related cholestasis, acute viral hepatitis, cirrhosis, liver tumors- hepatocellular adenoma, malignant hepatoma, recent major surgery with prolonged immobilization, being less than six months postpartum, using injectable hormonal contraceptive use within last six months, abnormal cervical smear diagnosed during screening, currently using or used within last two months drugs that interfere with the metabolism of contraceptive hormones. | Mean age (SD):  G1*: 27 (3.36)  G2: 26.7 (3.39)  N (%) married: NR  N (%) nulliparous: NR  N (%) rural: NR | Unclear |
| Abnormal uterine bleeding | | | | | |
| Dahiya;  2016;  India;  NR;  NR | Prospective randomized study;  Obstetrics and Gynecology department;  G1*: Contraceptive vaginal ring (N=25)  G2: COC (N=25) | NA | Inclusion: Patients in reproductive age group (18 to 50 years), fibroid <4 cm, no other pelvic pathology and not on hormonal therapy for the last 6 months.  Exclusion: Women with known or suspected malignant condition of genital tract or breast, lactating women, any liver or heart disease, arterial or venous thrombosis, headache with focal neurological symptoms, severe hypertension, personal or family history of any bleeding disorder, vaginal/cervical infection, cervical descent, chronic constipation. | Mean age (SD):  G1*: 33.92 (2.96)  G2: 34.32 (3.11)  Married N (%): NR  Nulliparous N (%): NR  Rural N (%): NR | Low |
| Hashim;  2012;  Egypt;  None;  NR | Randomized controlled trial;  Outpatient clinic in Mansoura University Hospitals and a private practice setting;  G1*: Contraceptive vaginal ring (N=48)  G2: Norethisterone acetate tablets (N=47) | Concurrent, prospective | Inclusion: Heavy menstrual bleeding based on a PBAC^[[4]](#endnote-5)4^ score over 185 (mean of two control cycles), parous women desiring contraception and willing to use a male condom if required, aged between 20 and 35 years in good general health with a regular menstrual cycle with evidence of ovulation diagnosed when midluteal phase serum progesterone level was ≥5 ng/mL, a normal pelvic examination with a sound measurement of the uterus of <10 cm, no pathology identified in pelvic ultrasound, normal histology on endometrial biopsy, negative cervical smear and no contraindications.  Exclusion: Pregnancy; age >35 years; obesity (body mass index >30 kg/m^2^); smokers; current intrauterine contraceptive device users; abnormal uterine bleeding not fully investigated; hormone therapy or any medication that might affect the menstrual blood loss within the previous 3 months; women who used injectable hormones for contraception during the previous 12 months; use of drugs that interfere with contraceptive hormone metabolism; previous endometrial resection/ablation and other pathology, or heavy menstrual bleeding of endocrine or systemic origin. | Mean age (SD):  G1*: 27.8 (4.9)  G2: 28.2 (4.4)  N (%) married: NR  N (%) nulliparous: 0 (0%)  N (%) rural: NR | Low |
| Jain;  2016;  India;  Hospital budget;  NR | Randomized controlled trial;  Department of Obstetrics and Gynecology, Guru Teg Bahadur Hospital;  G1*: Nuvaring (N=30)  G2: COC (N=30, N=28 completed study) | Concurrent | Inclusion: Women in reproductive age group of 15 to 45 years suffering from abnormal uterine bleeding with no identifiable organic pathology.  Exclusion: Medical disorders like jaundice, migraine, epilepsy, hypertension or diabetes mellitus, history of thromboembolism, breast or genital tract malignancy, genital prolapse or infections. | Mean age (SD):  G1*: 36.00 (1.14)  G2: 34.48 (0.92)  N (%) married: NR  N (%) nulliparous: 0 (0%)  N (%) rural: NR | Low |
| Multipurpose prevention technology | | | | | |
| Minnis 2018, Weinrib 2018, van der Straten 2018;  South Africa, Kenya;  Bill & Melinda Gates Foundation;  TRIO | Randomized cross-over trial;  Impact Research and Development Organization in Kisumu, Kenya and Setshaba Resesarch Center in Soshanguve, South Africa;  G1: Placebo daily oral tablet (N=277)  G2: Placebo monthly injection of 2 mL saline (N=277)  G3*: Placebo monthly vaginal ring (N=277)  Baseline included animated educational video about MPT daily tablets, monthly injections, and vaginal rings, followed by acceptability questionnaire (N=277) | Prospective, concurrent | Inclusion: Women aged 18 to 30 years who were sexually active, non-pregnant, HIV-negative, and had never participated in HIV-prevention or MPT product trials or demonstration studies.  Exclusion: NR | Mean age (SD): 23.8 (NR)  N (%) married: 70 (25.3%)  N (%) nulliparous: 61 (22.0%)  N (%) rural: NR | Low |
| Thurman;  2018;  United States, Dominican Republic (DR);  USAID, PEPFAR;  CONRAD A13-128 | Randomized placebo-controlled phase I trial;  CONRAD Intramural Clinical Research Center, Eastern Virginia Medical School, PROFAMILIA, Santo Domingo;  G1*: TVF^[[5]](#endnote-6)^ vaginal ring (N=21, N=11 in DR)  G2*: TVF and LNG^[[6]](#endnote-7)^ vaginal ring (N=20, N=11 in DR)  G3*: Placebo vaginal ring (N=10, N=5 in DR) | Concurrent | Inclusion: Women who were healthy, 18 to 45 years old, had a body mass index less than 30 kg/m^2^ and reported no use of exogenous hormones and regular menstrual cycles.  Exclusion: Bacterial vaginosis, active HSV-2, Neisseria gonorrhoeae, Chlamydia trachomatis, Trichomonas vaginalis, HIV-1, Hepatitis B. | Mean age (SD): 34.1 (4.70) for DR  N (%) married: NR for DR  N (%) nulliparous: NR for DR  N (%) rural: NR for DR | Low |
| Chronic pelvic pain | | | | | |
| Priya;  2016;  India;  No external funding;  NR | Randomized prospective interventional study;  Gynecology Department of University College of Medical Sciences & Guru Teg Bahadur  Hospital;  G1*: Nuvaring (N=30)  G2: COC (N=30) | Concurrent | Inclusion: Women 18 to 45 years with chronic pelvic pain for more than six months and no definite cause by history, examination, transabdominal, transvaginal ultrasonography and laparoscopy.  Exclusion: Gynecologic or gastrointestinal disorders, urinary problems, musculoskeletal disorders, neurological diseases, or psychosocial issues, postmenopausal women, treatment other than NSAIDs up to three months before study entry, women planning pregnancy and those with contraindications to use of estrogens and progestins. | Mean age (SD):  G1: 31.6 (5.9)  G2: 33.5 (5.8)  N (%) married:  G1: NR  G2: NR  N (%) nulliparous:  G1: 0 (0%)  G2: 0 (0%)  N (%) rural:  G1: NR  G2: NR | Unclear |

Table 1b. Acceptability outcomes of randomized controlled trials and clinical trials addressing vaginal ring acceptability, by indication

| **Author;**  **Year;**  **Country;**  **Funding;**  **Trial or study name** | **Product attributes** | **Global acceptability outcomes: N (%)** | **Secondary acceptability outcomes: N (%)** | | **Choice/use outcomes: N (%)** | **Values and preferences outcomes: N (%)** |
| --- | --- | --- | --- | --- | --- | --- |
|  |  |  | **Benefits** | **Harms** |  |  |
| HIV prevention | | | | | | |
| Baeten 2016, Palanee-Phillips 2018, Mensch 2019;  Malawi, South Africa, Uganda, Zimbabwe;  National Institutes of Health;  MTN 020/ASPIRE | G1*:  Materials: Silicone elastomer  Dimensions: NR (IPM)  Dose: 25 mg of dapivirine administered monthly, no ring-free period  G2*:  Materials: Silicone elastomer  Dimensions: NR (IPM)  Dose: Placebo, administered monthly, no ring-free period | NR | Palanee-Phillips, 2018: Partner aware of ring use: 1680 (64.2%) | Baeten 2016: Serious adverse events related to ring use, N (%):  G1: 0 (0%)  G2: 0 (0%)  Palanee-Phillips 2018:Worried about ring use, baseline, N (%):  Very worried: 39 (1.5%)  Somewhat worried: 737 (28.0%)  Not at all worried: 1853 (70.5%)  Palanee-Phillips 2018: Worried partner will feel ring, baseline, N (%): 459 (17.5%)  Palanee-Phillips 2018:Worried partner will not like/approve of ring, baseline, N (%): 253 (9.6%)  Palanee-Phillips 2018: Any partner-related ring worries, N (%): 547 (20.8%)  Palanee-Phillips 2018: Experience of social harm^[[7]](#endnote-8)^ during study: Worried partner will feel ring (vs. not worried), baseline: RR=1.75 (95%CI 1.09, 2.80); Worried partner will not like/approve of ring (vs. not worried), baseline: RR=1.85 (95%CI 1.06, 3.24)  Palanee-Phillips 2018:N=94 social harms were reported, of which N=87 were partner, N=2 were family, N=1 was personal, N=2 were employment, N=1 was housing, and N=1 was other (community rumor) related.  Palanee-Phillips 2018:Triggers of partner-related social harms, N (%):  Discovery of the ring during sex or foreplay: 23 (26.4%)  Partner notification of a sexually transmitted infection: 4 (4.6%)  Partner suspicion that  the ring was associated with witchcraft, promiscuity, or ill health: 16 (18.4%) | Palanee-Phillips 2018: Instances ring declined at a scheduled visit (out of 51,614 visits), N (%): 105 (0.2%)  Palanee-Phillips 2018: More likely to decline ring if experienced a social harm: aRR=18.18 (95%CI 7.39 to 44.70, P<0.001).  Adjusted for age, study site, time on study  Mensch 2019: G1* only, visits in which participants self-reported ring ever out, N (%):  Visits with self-report, plasma, and residual ring data (N=6,245 visits): NR (4.1%)  Visits with self-report and plasma data (N=7,631 visits): NR (5.5%)  Mensch 2019: G1* only, visits in which participants self-reported ring ever out more than 12 hours, N (%):  Visits with self-report, plasma, and residual ring data (N=6,245 visits): NR (1.4%)  Visits with self-report and plasma data (N=7,631 visits): NR (2.5%)  Mensch 2019: G1* only, reasons for the ring being removed, N (%):  G1* participants age 18 to 21 (N=117)  Physical/hygienic: 20 (17.1%)  Study related/procedural: 43 (36.8%)  Social/sexual: 16 (13.7%)  Came out on its own: 10 (8.5%)  G1* participants age ≥22 (N=301)  Physical/hygienic: 69 (22.9%)  Study related/procedural: 93 (30.9%)  Social/sexual: 42 (14.0%)  Came out on its own: 24 (8.0%)  Baeten 2016: Quarterly study retention, N (%):  G1*: 1290 (98.5%) 3 mo., 1243 (96.2%) 6 mo., 1206 (94.3%) 9mo., 1180 (93.1%) 12 mo., 1040 (91.1%) 15 mo., 876 (89.9%) 18 mo., 761 (88.3%) 21 mo., 645 (87.2%) 24 mo., 523 (86.4%) 27 mo., 361 (86.4%) 30 mo., 171 (85.9%) 33 mo.  G2*: 1287 (98.2%) 3 mo., 1244 (96.7%) 6 mo., 1206 (95.0%) 9 mo., 1180 (93.3%) 12 mo., 1035 (92.1%) 15 mo., 873 (90.7%) 18 mo., 752 (89.6%) 21 mo., 637 (89.3%) 24 mo., 517 (88.5%) 27 mo., 354 (87.0%) 30 mo., 157 (87.2%) 33 mo. | NR |
| Hardy;  2007;  Brazil;  CONRAD, FAPESP, and FAEPEX/Unicamp;  NR | G1:  Materials: Silicone  Dimensions: 65mm  Dose: 3g of gel with no substances administered prior to sexual intercourse  G2*:  Materials: NR (NuvaRing)  Dimensions: 54mm x 4mm  Dose: 0.12 mg Esonogestrel and 0.015 mg EE^[[8]](#endnote-9)^ (used because placebo ring NA) administered monthly  G3:  Materials: Plastic applicator for lubricant gel  Dimensions: 12cm x 0.9cm  Dose: 3g of gel with no active substances administered prior to sexual intercourse | Persons reporting liking the device the most, self-reported, N (%):  G1: 41 (10.7%)  G2*: 202 (52.9%)  G3: 139 (36.4%)  Persons reporting liking the device the least, self-reported, N (%):  G1: 252 (66.7%)  G2*: 64 (16.9%)  G3: 62 (16.4%) | NR | Difficulty inserting and/or removing, self-reported, N (%):  G1: 258 (65.5%)  G2*: 55 (14.1%)  G3: 12 (3.0%)  Difficulty inserting and/or removing, partner-reported, N (%):  G1: 150 (38.8%)  G2*: 28 (7.2%)  G3: 16 (4.0%) | Incorrect use (failed to adhere to instructions for its use), self-reported, N (%):  G1: 156 (39.6%)  G2*: 43 (11.0%)  G3: 23 (5.8%)  Incorrect use (failed to adhere to instructions for its use), partner-reported, N (%):  G1: 101 (26.0%)  G2*: 32 (8.2%)  G3: 14 (3.5%) | NR |
| Nel;  2016;  Kenya, Malawi, South Africa, Tanzania;  International Partnership for Microbicides;  NR | G1*:  Materials: Platinum-catalyzed silicone  Dimensions: 56mm x 7.7mm  Dose: 25mg dapivirine administered 4-weekly  G2*:  Materials: Platinum-catalyzed silicone  Dimensions: 56mm x 7.7mm  Dose: None administered 4-weekly | Acceptability (if thought at risk of HIV, willingness to use ring if effective) G1* and G2*, N (%):  4wk: 265 (96%)  12wk: 157 (96%) | Ring usually comfortable, G1* and G2*, N (%):  4wk: NR (96%)  12wk: NR (97%)  Ring easy to insert (at last insertion), G1* and G2*, N (%):  4wk: 261 (95%)  12wk: 243 (95%)  Never aware of ring during daily activities, G1* and G2*, N (%):  4wk: 233 (85%)  12wk: 237 (89%)  Wearing ring daily was usually comfortable, G1* and G2*, N (%):  4wk: 265 (95%)  12wk: 257 (97%)  Never felt ring during sex, G1* and G2*, N (%):  4wk: 233 (87%)  12wk: 242 (92%)  Partner did not feel the ring during sex, G1* and G2*, N (%):  4wk: 165 (61%)  12wk: 169 (63%)  Partner felt the ring during sex, but it was not a problem, G1* and G2*, N (%):  4wk: 55 (20%)  12wk: 60 (22%)  Does not know if partner felt the ring during sex, G1* and G2*, N (%):  4wk: 42 (16%)  12wk: 34 (13%) | Ring usually uncomfortable, G1* and G2*, N (%):  4wk: 3 (1%)  12wk: 3 (1%)  Concerned that ring may get lost or stuck in the body, G1* and G2*, N (%):  4wk: 76 (28%)  12wk: 59 (22%)  Concerned that ring may fall out, G1* and G2*, N (%):  4wk: 60 (20%)  12wk: 42 (16%)  Ring out of vagina (expelled or removed) , G1* and G2*, N (%):  2wk: 24 (9%)  4wk: 8 (3%)  8wk: 10 (4%)  12wk: 11 (4%)  Expulsions, G1* and G2*, N: 20  Defecation and/or urination was associated with 70% of expulsions.  Vaginal discharge, N (%):  G1*: 10 (7.1%)  G2*: 7 (5.0%)  Metrorrhagia (intermenstrual bleeding or breakthrough bleeding), N(%):  G1*: 26 (18.6%)  G2*: 27 (19.3%) | NR | Prefers daily (versus non-daily use), G1* and G2*, N (%):  4wk: 264 (95%)  12wk: 258 (97%)  Prefer to wear during menses (versus not) , G1* and G2*, N (%):  4wk: 190 (69%)  12wk: 182 (69%)  Important that partner does not feel ring during sex, G1* and G2*, N (%):  4wk: 144 (53%)  12wk: 132 (49%) |
| van der Straten 2012, Montgomery 2012, Nel 2018;  Tanzania, South Africa;  NIH;  IPM 011 | G1*:  Materials: Silicone elastomer  Dimensions: 56mm x 7.7mm  Dose: None, worn continuously  G2:  Materials: NA  Dimensions: NA  Dose: NA | van der Straten 2012: Willing to use ring if it were effective, wk. 12, N (%): NR (100%)  van der Straten 2012: Interest in using ring after first hearing about it, prior to use, N (%):  Very keen: NR (69%)  A little keen: NR (25%)  Not at all keen: NR (6%)  van der Straten 2012: Felt ring was better than had first thought, during use, N (%): 81 (52.6%) wk. 4, 72 (47.4%) wk. 12  Felt ring was worse than first thought, during use, N (%): 0 (0%) wk. 4, 0 (0%) wk. 12 | van der Straten 2012: Successfully inserted ring at first attempt, N (%): 127 (81%)  van der Straten 2012: Required staff assistance for insertion, N (%): 5 (3.2%)  van der Straten 2012: Ring easy to insert last time they tried, N (%): 137 (89.5%) wk. 4, 143 (94.7%) wk. 12  van der Straten 2012: Ring very easy to remove, N (%): 134 (88.2%) wk. 4, 140 (92.1%) wk. 12  van der Straten 2012: Ring very comfortable, N (%): 147 (95.5%) wk. 4, 144 (94.7%) wk. 12  van der Straten 2012: Disclosed study participation to partner, N (%): NR (78%) prior to ring use, NR (90%) prior to wk. 4, NR (93%) prior to wk. 12. | van der Straten 2012: Concerned ring would get lost in body, N (%): 31 (20.1%) wk. 4, 28 (18.4%) wk. 12; 11 (7%) at all visits  van der Straten 2012: Checked once a day or more to ensure ring was in place, N (%): 31 (20.1%) wk. 4, 25 (16.4%) wk. 12  van der Straten 2012: Checked once a week or more (but less than once a day) , N (%): 40 (26%) wk. 4, 53 (34.9%) wk. 12  van der Straten 2012: Concerned about ring getting lost in the body:  Durban (vs. Moshi) aOR=2.56 (95%CI: 1.12, 5.84); think about ring sometimes/often (vs. rarely) aOR=2.16 (95%CI: 1.28, 3.65); Ring removal very easy (vs. less than very easy) aOR=0.38 (95%CI: 0.19, 0.76); Johannesburg (vs. Moshi) aOR=1.84 (95%CI 0.79, 4.28); Cape Town (vs. Moshi) aOR=0.78 (95%CI 0.25, 2.50)  Adjusting NR  van der Straten 2012: Concerned about ring coming out, N (%): 18 (11.7%) wk. 4, 11 (7.2%) wk. 12; 2 (1.32%) at all visits  van der Straten 2012: Physical problems with ring, N (%): 5 (3.2%) wk. 4, 0 (0%) wk. 12.  van der Straten 2012: At wk. 4, N=2 of 5 experiencing physical problems said it made them want to stop using ring  van der Straten 2012: Emotional problems (fear) due to using ring, N (%): 1 (0.6%) wk. 4, 0 (0%) at wk. 12; 2 (1.3%) at any time point  van der Straten 2012: At any time point, N=2 of 2 experiencing emotional problems said it made them want to stop using ring  van der Straten 2012: Thought often/sometimes about ring in past 7 days, N (%): 40 (26%) wk. 4, 26 (17.1%) wk. 12  Thought often/sometimes about ring, wk. 12 (vs. wk. 4)  OR=0.58 (95%CI 0.36, 0.94)  van der Straten 2012: Felt ring sometimes/often during normal activities, N (%): 6 (3.9%) wk. 4, 2 (1.3%) wk. 12; 1 (0.64%) at all visits  van der Straten 2012: Partner might become angry if he were not told about ring, wk. 12, N (%): 95 (63%)  van der Straten 2012: Partner might become violent if he were told about ring, wk. 12, N (%): 20 (13% of study, 21% of those who said partner might become angry)  Nel 2018: Vaginal discharge, N (%):  During ring intervention phase: 7 (4.4%)  During observation phase: 3 (1.9%) | Montgomery 2012: Ring coming out since last visit, N (%):  4 wk: 140 (91%) never, 14 (9%) 1 time  8 wk: 144 (93%) never, 10 (6%) 1 time, 1 (1%) 2 times  12 wk: 142 (95%) never, 6 (4%) 1 time, 1 (1%) 2 times    Montgomery 2012: Among those reporting ring out since last visit:  Ring came out on its own, N (%): 6 (43%) wk. 4, 7 (64%) wk. 8, 3 (38%) wk. 12  Participant removed ring, N (%): 8 (57%) wk. 4, 4 (36%) wk. 8, 5 (63%) wk. 12  Reasons for ring coming out on its own, N (%):  During defecation: 1 (14%) wk. 8  During urination: 1 (14%) wk. 8, 1 (33%) wk. 12  During sex: 2 (40%) wk. 4  Menses-related: 1 (20%) wk. 4, 4 (57%) wk. 8, 2 (67%) wk. 12  Other: 2 (40%) wk. 4, 1 (14%) wk. 8  Reasons for participant removing ring, N (%):  During defecation: 1 (14%) wk. 8  During urination: 1 (14%) wk. 8, 1 (33%) wk. 12  During sex: 2 (40%) wk. 4  Menses-related: 1 (20%) wk. 4, 4 (57%) wk. 8, 2 (67%) wk. 12  Other: 2 (40%) wk. 4, 1 (14%) wk. 8  Montgomery 2012: Among women reporting a ring removal:  Reason for removal, N (%):  Discomfort: 1 (13%) wk. 4  Felt ring was not in place: 0 (0%)  Didn't want partner to know about it: 0 (0%)  Partner told them to remove it: 2 (25%) wk. 4  Menses-related: 2 (25%) wk. 4, 1 (25%) wk. 8, 1 (20%) wk. 12  Other: 3 (38%) wk. 4, 3 (75%) wk. 8, 4 (80%) wk. 12  Montgomery 2012: Ring never removed at wk. 12: Use of vaginal products for menstruation (vs. no use) aOR=0.22 (95%CI 0.05, 0.89); Johannesburg (vs. Cape Town) aOR=6.23 (95%CI 1.21, 32.17); Married (vs. not married) aOR=0.69 (95%CI 0.18, 2.60); Moshi (vs. Cape Town) aOR=8.59 (95%CI 1.00, 73.38); Durban (vs. Cape Town) aOR=2.42 (95%CI 0.47, 12.46); Ever had concerns about VR coming out (vs. never) aOR=0.77 (95%CI 0.23, 2.61)  Adjusted for marital status, perceived HIV risk, ever use of male condom, frequency of male condom use, concerns about VR coming out, and ever experienced physical problems with ring.  Nel 2018: Trial completion, wk. 12, G1* and G2, N (%): 145 (85.3%)  Nel 2018: Reasons for early trial discontinuation, G1* and G2, N (%):  Loss to follow-up: 9 (NR)  Non-compliance: 2 (NR)  Inappropriate enrollment: 1 (NR)  Other^[[9]](#endnote-10)^: 12 (NR) | van der Straten 2012: Liked having the ring in place every day, wk. 12, N (%): NR (96%)  van der Straten 2012: Partner's approval of ring was important, wk. 12, N (%): NR (86%)  van der Straten 2012:Willing to use both condoms and ring for sex if recommended, wk. 12, N (%): NR (81%)  van der Straten 2012: Important that partner would not notice ring during sex, wk. 12, N (%): NR (67%)  van der Straten 2012: Would use ring without telling partner, wk. 12, N (%): 97 (65%)  van der Straten 2012: Thought it was important that the ring can be used without partner’s knowledge, wk. 12, N (%): NR (59%)  van der Straten 2012: Most liked product characteristics at wk. 12: (1) "might someday be used to prevent HIV," (2) "does not interfere with 'normal' or 'natural' sex," (3) "you must use a condom with the VR during the study."  van der Straten 2012: Least liked product characteristics at wk. 12: (1) "it might get lost in the body," (2) "it might come out during sex," (3) "it might change the feeling of sex for the male partner" |
| Contraception | | | | | | |
| Fan;  2016;  China;  Merck;  NR | G1*:  Materials: Ethylene-vinyl acetate  Dimensions: NR (NuvaRing)  Dose: 120 μg etonogestrel and  15 μg EE/day,  administered monthly, one ring-free week  G2:  Materials: NA  Dimensions: NA  Dose: 3 mg DSRP and 30 ug EE taken daily, one pill-free week per month | NR | Breakthrough bleeding/spotting, N (%):  Cycle 1:  G1*: 111 (18.6%)  G2: 43 (21.6%)  Cycle 2:  G1*: 74 (12.3%)  G2: 36 (18.5%)  P=0.029  Cycle 3:  G1*: 49 (8.5%)  G2: 28 (14.3%)  P=0.019  Cycle 4:  G1*: 44 (7.5%)  G2: 30 (15.5%)  P=0.001  Cycle 5:  G1*: 40 (6.8%)  G2: 15 (8.0%)  Cycle 6:  G1*: 41 (6.9%)  G2: 20 (10.8%)  Cycle 7:  G1*: 44 (7.5%)  G2: 21 (11.3%)  Cycle 8:  G1*: 37 (6.3%)  G2: 20 (10.6%)  P=0.049  Cycle 9:  G1*: 40 (6.9%)  G2: 15 (8.0%)  Cycle 10:  G1*: 34 (5.9%)  G2: 19 (10.4%)  P=0.041  Cycle 11:  G1*: 24 (4.2%)  G2: 14 (7.9%)  P=0.046  Cycle 12:  G1*: 30 (5.3%)  G2: 17 (9.7%)  P=0.037  Cycle 13:  G1*: 35 (6.4%)  G2: 19 (11.0%)  P=0.048  Absence of withdrawal bleeding, N (%):  Cycle 1:  G1*: 51 (8.6%)  G2: 29 (14.6%)  P=0.015  Cycle 2:  G1*: 29 (4.8%)  G2: 19 (9.7%)  P=0.012  Cycle 3:  G1*: 30 (5.2%)  G2: 23 (11.7%)  P=0.002  Cycle 4:  G1*: 24 (4.1%)  G2: 15 (7.7%)  P=0.044  Cycle 5:  G1*: 32 (5.4%)  G2: 12 (6.4%)  Cycle 6:  G1*: 34 (5.8%)  G2: 18 (9.7%)  Cycle 7:  G1*: 20 (3.4%)  G2: 14 (7.5%)  P=0.019  Cycle 8:  G1*: 29 (5.0%)  G2: 13 (6.9%)  Cycle 9:  G1*: 25 (4.3%)  G2: 16 (8.6%)  P=0.027  Cycle 10:  G1*: 21 (3.7%)  G2: 12 (6.6%)  Cycle 11:  G1*: 17 (3.0%)  G2: 13 (7.3%)  P=0.009  Cycle 12:  G1*: 23 (4.0%)  G2: 18 (10.2%)  P=0.002  Cycle 13:  G1*: 32 (5.9%)  G2: 18 (10.4%)  P=0.043 | Expulsion, N (%):  G1*: 16 (2.2%)  G2: NA  Vaginal infection, N (%):  G1*: 20 (2.8%)  G2: 4 (1.7%) | Reasons for discontinuation, N (%):  G1*:  Adverse events: 60 (8.4%)  Pregnancy: 9 (1.3%)  Lost to follow-up: 14 (2.0%)  Subject did not wish to continue, not treatment related: 7 (1.0%)  Subject did not wish to continue, treatment related: 10 (1.4%)  Subject withdrew consent: 9 (1.3%)  Non-compliance with protocol: 13 (1.8%)  Did not meet protocol eligibility: 1 (0.1%)  Administrative: 3 (0.4%)  Pregnancy wish: 0 (0%)  G2:  Adverse events: 22 (9.5%)  Pregnancy: 4 (1.7%)  Lost to follow-up: 9 (3.9%)  Subject did not wish to continue, not treatment related: 0 (0%)  Subject did not wish to continue, treatment related: 0 (0%)  Subject withdrew consent: 9 (3.9%)  Non-compliance with protocol: 4 (1.7%)  Did not meet protocol eligibility: 0 (0%)  Administrative: 0 (0%)  Pregnancy wish: 2 (0.9%)  Cycles compliant with regimen, N (%):  G1*: NR (92.7%)  G2: NR (97.6%)  Participants using ring without temporary removals, G1* only, N (%): NR (70.1%)  Participants temporarily removed ring for ≤2 hours, G1* only, N (%): NR (12.1%)  Participants temporarily removed ring for >24 hours, G1* only, N (%): NR (2.1%) | NR |
| Kestelyn 2018a, Kestelyn 2018b;  Rwanda;  European and Developing Countries Clinical Trials Partnership;  NR | G1*:  Materials: NR (NuvaRing)  Dimensions: NR x 4 mm (NuvaRing)  Dose: 0.120 mg etonogestrel  and 0.015 mg EE/day, administered monthly, one ring-free week per month  G2*:  Materials: NR (NuvaRing)  Dimensions: NR x 4 mm (NuvaRing)  Dose: 0.120 mg etonogestrel  and 0.015 mg EE/day, administered 3-weekly, no ring-free period | Kestelyn 2018a: G1* and G2*, would recommend the vaginal ring to others, N (%):  Prior to study: 1 (0.83%)  End of study: 118 (98.3%) | Kestelyn 2018a: G1* and G2*, Thought that vaginal ring was the best method of family planning, N (%):  Prior to study: 1 (0.83%)  End of study: 118 (98.3%)  Kestelyn 2018a: G1* and G2*, told main partner about the ring/study, N (%):  Prior to study: 112 (93.3%)  End of study: 119 (99.2%)  Kestelyn 2018a: G1* and G2*, never felt ring during sex at end of study, N (%): 99 (82.5%)  Kestelyn 2018a: G1* and G2*, main partner felt ring during sex at least once at end of study, N (%): 63 (52.5%)  Kestelyn 2018b: G1* and G2*, male partner thoughts about feeling ring, N (%):  Indifferent: NR (33%)  Liked how it felt: NR (19%)  Kestelyn 2018a: G1* and G2*, main partner disliked the way ring felt during sex at least once, N (%):  During study: 4 (3.33%)  End of study: 0 (0%)  Kestelyn 2018a: G1* and G2*, Ring made sex feel better, N (%):  During study: 97 (80.6%)  End of study: 105 (87.5%)  Kestelyn 2018a: G1* and G2*, insertion was painful immediately after first inserting ring, N (%): 2 (1.67%)  Kestelyn 2018a: G1* and G2*, thought that reinsertion would be easy, N (%):  Immediately after first inserting ring: 116 (96.7%)  End of study: 119 (99.2%)  Kestelyn 2018a: G1* and G2*, increased vaginal wetness or lubrication, considered positive attribute, N (%):  During study: NR (52.9%)  End of study: NR (74.8%)  Kestelyn 2018a: G1* and G2*, increased vaginal wetness or lubrication was problematic^[[10]](#endnote-11)^, N (%):  During study: 2 (1.67%)  End of study: 1 (0.83%)  Kestelyn 2018b: Abnormal vaginal discharge, N (%):  G1*: 0 (0%)  G2*: 0 (0%)  Kestelyn 2018a: G1* and G2*, discontinued menstrual periods at end of study, N (%): 69 (65.8%)  Kestelyn 2018b: No bleeding days during ring use, final ring removal visit (3 wk/1 mo) , N (%):  G1*: 6 (10%)  G2*: 34 (57%)  Kestelyn 2018b: Felt that having no bleeding was a problem, N (%): G1*: 0 (0%)  G2*: 1 (1.7%)  Kestelyn 2018b: Any vaginal bleeding not attributed to menses or withdrawal bleeding, N (%):  G1*: 3 (5%) first ring removal visit 1 (1.7%) at second ring removal visit, 0 (0%) subsequent visits  G2*: 1 (1.7%) first ring removal visit, 0 (0%) subsequent visits  Kestelyn 2018a: G1* and G2*, discontinued menstrual periods was problematic, N (%): 1 (0.83%) | Kestelyn 2018a: G1* and G2*, at least one worry about ring use, N (%):  During study: 19 (16%)  End of study: 4 (3.33%) (N=2 worried about partner not liking the ring, N=2 worried about ring expulsion)  Kestelyn 2018b: G1* and G2*, worries about ring use prior to insertion, N (%):  Main partner might not like the ring: NR (13%)  Ring might come out spontaneously: NR (11%)  Ring might be uncomfortable during sex: NR (9%)  Ring might cause infection: NR (8%)  Ring might not adequately protect against pregnancy: NR (8%)  Kestelyn 2018b: G1* and G2*, Ring removal visits in which previous ring expulsion was reported (N=416), N (%): 51 (12.3%)  Kestelyn 2018b: Reasons ring was expelled, within ring use periods in which one expulsion took place (N=58), N (%):  Came out on its own: 45 (77.6%) (most commonly during/after sex: 15 (25.9%), during urination: 12 (20.7%), or during defecation: 11 (19.0%))  Removed by woman: 13 (22.4%)  Removed by woman, causing discomfort: 4 (6.9%)  Removed by woman, perceived to be incorrectly placed: 3 (5.2%)  Removed by woman, partner wanted it taken out: 1 (1.7%)  Removed by woman, other reasons: 5 (8.6%)  Removed by husband: 2 (3.4%) | NR | Kestelyn 2018a: G1* and G2*, liked that they did not feel ring during daily activities, N (%):  During study: NR (95.4%)  End of study: NR (95.8%)  Kestelyn 2018a: G1* and G2*, felt it was important their main partner would not feel the ring during sex prior to study, N (%): 70 (58.3%)  Kestelyn 2018a: G1* and G2*, worries about using the vaginal ring prior to study (multiple responses possible), N (%):  At least one worry: NR (31.7%)  Ring might come out: NR (10.8%)  Be uncomfortable during sex: NR (9.2%)  Cause infection: NR (7.5%)  Not adequately protect against pregnancy: NR (7.5%)  Kestelyn 2018a: G1* and G2*, partner refused for women to participate in the study (N=351 screened), N (%): 8 (2.3%) |
| Mohamed;  2011;  Egypt;  NR;  NR | G1*:  Materials: NR (NuvaRing)  Dimensions: NR (NuvaRing)  Dose: NR, administered 4-weekly with 1 ring-free week  G2:  Materials: NR (COC^[[11]](#endnote-12)^)  Dimensions: NA  Dose: 30 µg EE and 3 mg drospirenone, presumably administered daily for 3 weeks with 1 week of hormone-free pills | NR | Breakthrough bleeding, N (%):  G1*:  Early: 14 (5.9%)  Midcycle: 4 (1.7%)  Late: 9 (3.8%)  G2:  Early: 18 (7.3%)  Midcycle: 5 (2.0%)  Late: 13 (5.3%)  Any breakthrough bleeding lower in G1* than G2, N (%):  11.3% vs. 14.7%; P<0.05 | Vaginitis, N (%):  G1*: 11 (4.6%)  G2: 3 (1.2%)  P<0.05  Decreased libido, N (%):  G1*: 8 (3.3%)  G2: 2 (0.8%)  Ring-related problems, N (%):  G1*: 16 (6.7%)  G2: NA  Withdrawal bleeding, N (%):  G1*:  Early: 26 (10.9%)  Late: 14 (5.9%)  G2:  Early: 19 (7.8%)  Late: 16 (6.5%) | Retention, N (%):  G1*: 263 (87.7%) at 3 mo., 248 (82.7%) at 6 mo., 244 (81.3%) at 9 mo., 239 (79.7%) at 12 mo.  G2: 270 (90.0%) at 3 mo., 259 (86.3%) at 6 mo., 251 (83.7%) at 9 mo., 245 (81.7%) at 12 mo.  Among those who discontinued, reasons for discontinuation (G1* N=61, G2 N=55), N (%):  G1*:  Adverse effects: 27 (9.0%)  Wished to become pregnant: 2 (0.7%)  G2:  Adverse effects: 17 (56.7%)  Wished to become pregnant: 3 (1.0%)  Became pregnant: 2 (0.7%) | NR |
| Sharma;  2018;  India;  NR;  NR | G1*:  Materials: NR (NuvaRing)  Dimensions: NR (NuvaRing)  Dose: NR (NuvaRing), administered monthly with one ring-free week per month    G2:  Materials: NR (Mala D)  Dimensions: NR (Mala D)  Dose: 30 µg EE and 150 µg LNG^[[12]](#endnote-13)^ per day, administered daily with one pill-free week per month | Acceptability, N (%):  G1*: 205 (95.34%)  G2: 200 (93.02%) | Felt ring during use, G1* only, N (%): 0 (0%)  Partner felt ring during intercourse but did not object to use of ring, G1* only, N (%): 1 (0.47%)  Ring use bothersome to users or partners, G1* only, N (%): 0 (0%)  "Majority of women never had a problem with insertion or removal of NuvaRing during the trial." | Vaginal discharge, N (%):  G1*: 22 (10.23%)  G2: 3 (1.4%)  P=0.0009 | Compliance^[[13]](#endnote-14)^, N (%):  G1*: 200 (93.02%)  G2: 197 (91.62%)  Discontinued due to adverse events, N (%):  G1*: 0 (0%)  G2: 0 (0%) | NR |
| Abnormal uterine bleeding | | | | | | |
| Dahiya;  2016;  India;  NR;  NR | G1*:  Materials: Ethinyl vinyl acetate (Evatane)  Dimensions: 54mm x 4mm  Dose: 15 μg of EE and 120 μg of etonogestrel/day, administered monthly with one ring-free week  G2:  Materials: NA  Dimensions: NA  Dose: 30 μgm EE and 150 μgm LNG, administered daily, one pill-free week per month | NR | Mean (SD) PBAC^[[14]](#endnote-15)^ value:  G1*: 327.84 (47.71) baseline, 96.96 (7.68) endline, 70.73% reduction.  G2: 331.36 (56.60) baseline, 99.32 (8.60) endline, 70.02% reduction  Mean (SD) days bleeding per cycle:  G1*:  7.84 (1.07) cycle 1,  6.6 (1.00) cycle 2, 6.00 (1.15) cycle 3, 5.32 (0.85) cycle 4, 4.68 (0.80) cycle 5, 4.64 (0.86) cycle 6  G2:  7.68 (1.18) cycle 1, 6.50 (1.02) cycle 2, 5.84 (0.69) cycle 3, 5.42 (0.93) cycle 4, 4.88 (0.83) cycle 5, 4.60 (0.87) cycle 6  Incidence of intended bleeding pattern (withdrawal bleeding in method-free period, no early or late withdrawal bleeding, no irregular bleeding) in G1* was significantly higher in cycle 3, 4, 5 and 6 (Data NR)  Continuous withdrawal bleeding/spotting per cycle, N (%):  G1*:  15 (60.0%) cycle 1, 11 (44.0%) cycle 2, 5 (20.0%) cycle 3, 3 (12.0%) cycle 4, 4 (16.0%) cycle 5, 1 (4.0%) cycle 6  G2:  18 (72.0%) cycle 1, 12 (48.0%) cycle 2, 14 (56.0%) cycle 3, 11 (44.0%) cycle 4, 12 (48.0%) cycle 5, 7 (28.0%) cycle 6  Difference between groups significant at 3 weeks (P=0.014), 4 weeks (P=0.049), 5 weeks (P=0.03), 6 weeks (P=0.04)  Intermenstrual bleeding per cycle, N (%):  G1*:  4 (16.0%) cycle 1, 3 (12.0%) cycle 2, 1 (4.0%) cycle 3, 0 (0.0%) cycle 4, 1 (4.0%) cycle 5, 0 (0.0%) cycle 6  G2:  6 (24.0%) cycle 1, 5 (20.0%) cycle 2, 7 (28.0%) cycle 3, 6 (24.0%) cycle 4, 5 (20.0%) cycle 5, 2 (8.0%) cycle 6  Difference between groups significant at 3 weeks (P=0.045) and 4 weeks (P=0.033) | Early withdrawal bleeding/spotting per cycle, N (%):  G1*:  3 (12.0%) cycle 1, 1 (4.0%) cycle 2, 0 (0.0%) cycle 3, 0 (0.0%) cycle 4, 2 (8.0%) cycle 5, 1 (4.0%) cycle 6  G2:  2 (8.0%) cycle 1, 1 (4.0%) cycle 2, 3 (12.0%) cycle 3, 1 (4.0%) cycle 4, 1 (4.0%) cycle 5, 0 (0.0%) cycle 6  Vaginitis, N (%):  G1*: 7 (28.0%)  G2: 4 (16.1%)  Vaginal discharge, N (%):  G1*: 4 (16.0%)  G2: 1 (4.0%)  Device-related adverse event, N (%):  G1*: 3 (12.0%)  G2: 0 (0%) | NR | NR |
| Hashim;  2012;  Egypt;  None;  NR | G1*:  Materials: NR (Nuvaring)  Dimensions: NR (Nuvaring)  Dose: NR (Nuvaring) administered 4-weekly with 1 ring-free week  G2:  Materials: Norethisterone acetate tablets  Dimensions: NA  Dose: 5mg three times daily | Overall very satisfied or satisfied with treatment:  G1*: 34 (70.8%)  G2: 20 (42.5%)  P=0.003 | PBAC score, 3 mo., mean (SD):  G1*: 90.2 (24.4)  G2: 92.3 (26.7)  NS^[[15]](#endnote-16)^  Reduction of PBAC score, %:  G1*: 68.6%  G2: 69.5%  NS  Duration of menses, days, mean (SD):  G1*: 5.3 (1.2)  G2: 5.5 (1.1)  NS  Breakthrough bleeding/spotting, N (%):  G1*: 2 (4.2%)  G2: 6 (12.8%)  P=0.02 | Ring-related events^[[16]](#endnote-17)^, N (%):  G1*: 3 (6.25%)  G2: NA  Vaginal discomfort, N (%):  G1*: 2 (4.2%)  G2: NA  Expulsions, N (%):  G1*: 0 (0%)  G2: NA | Completion of three cycles of treatment, N (%): 95 (100%)  Continuation with treatment after study, N (%):  G1*: 37 (77%)  G2: 12 (25.5%)  P=0.001  Discontinued treatment due to ring-related events, N (%):  G1*: 0 (0%)  G2: NA | NR |
| Jain;  2016;  India;  Hospital budget;  NR | G1*:  Materials: NR (Nuvaring)  Dimensions: NR (Nuvaring)  Dose: NR (Nuvaring) administered 4-weekly with 1 ring-free week  G2:  Materials: Combined oral contraceptive tablet (Mala N)  Dimensions: NA  Dose: 30µg EE and  150 µg LNG, administered daily, 1 week of hormone-free pills | Satisfaction, N (%):  G1*: 29 (96.7%)  G2: 23 (82%)  NS  Recommendation, N (%):  G1*: 27 (90%)  G2: 20 (71%)  NS | Ideal bleed, N (%):  G1*:  Cycle 1: 15 (50%)  Cycle 2: 20 (66.6%)  Cycle 3: 25 (83.3%)  G2:  Cycle 1: 12 (42.8%)  Cycle 2: 16 (57%)  Cycle 3: 20 (71.4%)  Coital difficulty or irritation reported by user or partner, N (%):  G1*: 0 (0%)  G2: NA | Major side effects, N (%):  G1*: 0 (0%)  G2: 0 (0%)  Minor side effects "seen more" in G2 (not statistically significant)  Expulsions, N (%):  G1*: 2 (6.7%)  G2: NA  PBAC scores, mean (SD):  G1*:  Baseline: 214.87 (86.29) Cycle 1: 102.13 (71.33)  Cycle 2: 87.90 (65.62)  Cycle 3: 87 (42.70)  G2:  Baseline: 237.57 (86.29) Cycle 1: 137.68 (61.56)  Cycle 2: 99.00 (66.01)  Cycle 3: 74.75 (51.58)  P value within group P<0.001, F=106.788  Late withdrawal bleed, N (%):  G1*:  Cycle 1: 12 (40%)  Cycle 2: 9 (30%)  Cycle 3: 4 (13.3%)  G2:  Cycle 1: 14 (50%)  Cycle 2: 8 (28.5%)  Cycle 3: 4 (14.2%)  Early withdrawal bleed, N (%):  G1*:  Cycle 1: 3 (10%)  Cycle 2: 1 (3.3%)  Cycle 3: 0 (0%)  G2:  Cycle 1: 0 (0%)  Cycle 2: 0 (0%)  Cycle 3: 0 (0%)  Breakthrough bleed, N (%):  G1*:  Cycle 1: 0 (0%)  Cycle 2: 0 (0%)  Cycle 3: 1 (3.3.%)  G2:  Cycle 1: 2 (7%)  Cycle 2: 4 (14%)  Cycle 3:4 (14.2%)  Leucorrhoea, N (%):  G1*: 6 (20%)  G2: 5 (17.8%)  P=1.0 | Continuation after end of study, N (%):  G1*: 27 (90%)  G2: 20 (71%)  NS  Reasons for discontinuation:  G1*: Persistence of dysmenorrhea  G2: Poor compliance, breakthrough bleed | NR |
| Multipurpose prevention technology | | | | | | |
| Minnis 2018, Weinrib 2018, van der Straten 2018;  South Africa, Kenya;  Bill & Melinda Gates Foundation;  TRIO | G1:  Materials: NA (Placebo Truvada, Gilead)  Dimensions: NA  Dose: None, administered daily  G2:  Materials: NA  Dimensions: NA  Dose: Two 2 mL saline injections, administered monthly  G3*:  Materials: Silicone elastomer (International Partnerships for Microbicide)  Dimensions: NR  Dose: None, administered monthly, no ring-free period | Weinrib 2018: Product approval ratings prior to use, N (%):  G1: 14% dislike very much, 15% dislike, 18% neutral, 37% like, 16% like very much  G2: 6% dislike very much, 12% dislike, 13% neutral, 34% like, 35% like very much  G3*: 23% dislike very much, 21% dislike, 21% neutral, 30% like, 6% like very much  Weinrib 2018, G3* only: Approval increased post-educational video by mean 0.35 points (p<0.001).  Minnis 2018: Mean (CI) score of "liking"^[[17]](#endnote-18)^ product after 1 mo. use:  G1: 2.96 (95%CI: 2.81, 3.12)  G2: 4.26 (95%CI: 4.14, 4.38)  G3*: 3.28 (95%CI: 3.11, 3.45)  Significantly higher for injection than other methods, (P<0.0001); significantly higher for rings than tablets (P=0.015)  Minnis 2018: Mean "liking" score before use:  G1: 3.25 (95%CI: 3.09, 3.40)  G2: 3.80 (95%CI: 3.66, 3.95)  G3*: 2.77 (95%CI: 2.61, 2.92)  Minnis 2018: Change in mean "liking" score from before use to after 1 mo. use:  G1: 0.24 (95%CI: 0.05, 0.42)  P=0.013  G2: 0.62 (95%CI: 0.45, 0.79)  P<0.001  G3*: 0.97 (95%CI: 0.75, 1.16)  P<0.001  van der Straten 2018: Disinterested in using product in future, after using each for 1 mo. (N=249), N (%):  G1: 108 (43%)  G2: 13 (5%)  G3*: 118 (47%)  Would consider using all: 16 (6%) | Minnis 2018: Acceptable look of product after 1 mo. use, N (%):  G1: 157 (61.6%)  G2: 224 (88.2%)  G3*: 169 (66.5%)  Minnis 2018: Acceptable ease of use after 1 mo. use, N (%):  G1: 149 (58.4%)  G2: 236 (92.9%)  G3*: 189 (74.4%)  Minnis 2018: Acceptable interference with normal activities after 1 mo. use, N (%):  G1: 170 (66.7%)  G2: 230 (90.6%)  G3*: 186 (73.2%)  Minnis 2018: Possible to use without partner knowledge after 1 mo. use, N (%):  G1: 176 (69.0%)  G2: 198 (78.0%)  G3*: 159 (62.6%)  Minnis 2018: Possible to use without family knowledge after 1 mo. use, N (%):  G1: 167 (65.5%)  G2: 186 (73.2%)  G3*: 204 (80.3%)  Minnis 2018: G3* only, after 1 mo. use:  Ring size acceptable, N (%): 156 (61.4%)  Ring felt acceptable during sex, N (%): 142 (55.9%)  Ring felt acceptable to partner during sex, N (%): 151 (59.7%)  Ring felt acceptable during menses, N (%): 137 (53.9%)  Leaving ring for month acceptable, N (%): 180 (71.7%)  Ring felt acceptable in hands, N (%): 156 (61.7%) | NR | Weinrib 2018: Stated product choice prior to use, N (%):  G1: 75 (27.1%)  Injections: 172 (62.1%)  G3*: 30 (10.8%)  Weinrib 2018: Stated product choice of ring over tablet: Women aged 25 to 30 (vs. 18 to 24) aRRR=3.1 (95%CI 1.2, 8.2); Prior contraceptive use of implants/IUD (vs. no use) aRRR=2.88 (95%CI 1.12, 7.45); women aged 25 to 30 (vs. 18 to 24) in Soshanguve aRRR=NR, NS; women aged 25 to 30 (vs. 18 to 24) in Kisumu aRRR=5.7 (95%CI 1.4, 23.4); Somewhat/very/extremely worried about HIV infection (vs. not at all/a little worried) aRRR=0.50 (95%CI 0.19, 1.32); Prior contraceptive use of pills (vs. no use) aRRR=0.49 (95%CI 0.17, 1.39); Prior contraceptive use of injectables (vs. no use) aRRR=0.74 (95%CI 0.28, 1.93)  Adjusted for age, study site, and randomization sequence.  van der Straten 2018: Product choice, after using each for 1 mo. (N=249), N (%):  G1: 52 (21%)  G2: 160 (64%)  G3*: 37 (15%)  van der Straten 2018: Product switching, after initial choice (N=249) , N (%):  G1:  To product: 22 (9%)  Away from product: 11 (4%)  G2:  To product: 16 (6%)  Away from product: 27 (11%)  G3*:  To product: 12 (5%)  Away from product: 12 (5%) | Weinrib 2018: Stated product choice of ring over tablet: Side effects as most/second most important attribute (Comparator NR) aRRR=0.45 (95%CI 0.12, 1.64); Availability as most/second most important attribute (Comparator NR) aRRR=0.34 (95%CI 0.10, 1.12); Frequency as most/second most important attributes (Comparator NR) aRRR=0.99 (0.23, 4.22)  Adjusted for age, study site, and randomization sequence.  Weinrib 2018: Among women who chose ring (N=30):  Most/second most important attribute, N (%):  Experience with vaginal insertion: 14 (46.7%)  Side effects/safety: 9 (30%)  Availability/access: 5 (20%)  Frequency of use: 4 (13.3%)  van der Straten 2018: Participants ranking product first in preference after using each for 1 mo. (N=249), N (%):  G1: 37 (15%)  G2: 155 (62%)  G3*: 26 (10%)  None, preferred condoms: 26 (10%)  van der Straten 2018: Participants ranking product last in preference after using each for 1 mo. (N=249), N (%):  G1: NR (25%)  G2: NR (5%)  G3*: NR (41%)  Condoms: NR (19%)  Minnis 2018: Ring ratings after 1 mo. use:  Ease of use acceptable (vs. unacceptable) β=1.81 (95%CI 1.47, 2.14); Acceptable to leave in ring for entire month (vs. unacceptable) β=1.68 (95%CI 1.35, 2.00); Interference with normal activities acceptable (vs. unacceptable) β=1.45 (95%CI 1.11, 1.79); Product look acceptable (vs. unacceptable) β=1.36 (95%CI 1.02, 1.70); Ring felt acceptable during sex (vs. unacceptable) β=1.36 (95%CI 1.01, 1.70); Ring felt acceptable to partner during sex (vs. unacceptable) β=1.12 (95%CI 0.74, 1.49); Ring felt acceptable during menses (vs. unacceptable) β=0.93 (95%CI 0.52, 1.34); Ring size acceptable (vs. unacceptable) β=0.80 (95%CI 0.46, 1.14); Inserting the ring acceptable (vs. unacceptable) β=0.78 (95%CI 0.41, 1.14); Ring felt acceptable in hands (vs. unacceptable) β=0.58 (95%CI 0.24, 0.93); Removing the ring acceptable (vs. unacceptable) β=0.53 (95%CI 0.12, 0.94); Possible to use without partner knowledge (Comparator NR) β=0.49 (95%CI 0.14, 0.84); Number of sex acts in past month (continuous) β=0.04 (95%CI 0.00, 0.07); Possible to use without family knowledge (comparator NR) β=-0.01 (95%CI -0.45, 0.44); More than one sex partner in past month (vs. 1 or 0) β=0.26 (95%CI -0.35, 0.87); any vaginal insertion in past 3 mo. (comparator NR) β=-0.20 (95%CI -0.56, 0.16); Ever used IUD or implant (comparator NR) β=0.25 (95%CI -0.1, 0.59); Has privacy in the home (comparator NR) β=0.39 (95%CI -0.05, 0.84); Often experiences food insecurity (vs. never) β=0.47 (95%CI -0.03, 0.97); Rarely or sometimes experiences food insecurity (vs. never) β=0.19 (95%CI -0.21, 0.58); Kenya (vs. South Africa) β=-0.32 (95%CI -0.66, 0.01); Married or cohabitating (comparator NR) β=-0.32 (95%CI -0.81, 0.17)  Adjusted for age, study site, randomization sequence    van der Straten 2018:  G1:  Product choice after 1 mo. use of all products: Has privacy in the home (comparator NR) aOR=0.47 (95%CI 0.22, 1.01)  Disinterested/unwilling to use in future after 1 mo. use of all products: Has privacy in the home (comparator NR) aOR=2.36 (95%CI 1.08, 5.14)  G3*:  Product choice after 1 mo. use of all products: Own source of income (comparator NR) aOR=0.53 (95%CI 0.23, 1.24); Does not have a casual sex partner (comparator NR) aOR=0.55 (95%CI 0.24, 1.23); Nulliparous (vs. parous) aOR=0.99 (95%CI 0.40, 2.49); Has privacy in home (comparator NR) aOR=2.29 (95%CI 0.73, 7.21)  Disinterested/unwilling to use in future after 1 mo. use of all products: Own source of income (comparator NR) aOR=2.05 (95%CI 1.09, 3.85); Does not have a casual sex partner (comparator NR) aOR=2.01 (95%CI 1.02, 3.94); Nulliparous (vs. parous) aOR=1.99 (95%CI 1.03, 3.86) Has privacy in home (comparator NR) aOR=0.38 (95%CI 0.18, 0.80)  Adjusted for country, randomization sequence, age |
| Thurman;  2018;  United States, Dominican Republic (DR);  USAID, PEPFAR;  CONRAD A13-128 | G1*:  Materials: Hydrophilic polyurethane reservoir sheath  Dimensions: 55mm x 5.5mm  Dose: 8 to 10 mg/day of TFV^[[18]](#endnote-19)^, worn for 15-18 days  G2*:  Materials: Hydrophilic polyurethane reservoir sheath  Dimensions: 55mm x 5.5mm  Dose: 8 to 10 mg/day of TFV and 20 μg LNG, worn for 15-18 days  G3*:  Materials: Hydrophilic polyurethane reservoir sheath  Dimensions: 55mm x 5.5mm  Dose: None, worn for 15-18 days | NR | NR | Partial or complete expulsion, DR only, N (%):  G1*: 0 (0%)  G2*: 0 (0%)  G3*: 0 (0%) | Continuation (completed all study visits, DR only), N (%):  G1*: 10 (90.9%)  G2*: 11 (100%)  G3*: 5 (100%) | NR |
| Chronic pelvic pain | | | | | | |
| Priya;  2016;  India;  No external funding;  NR;  NR | G1*:  Materials: NR (Nuvaring, Organon India Ltd.)  Dimensions: NR (Nuvaring, Organon India Ltd.)  Dose: 15µg EE and 120 µg etonogesterol per day given every 21 days, no hormone-free interval  G2:  Materials: NR (MALA-N, Indian Drugs & Pharmaceuticals)  Dimensions: NR  Dose: 30µg EE and 150 µg of LNG, administered daily, no hormone-free interval | Compliance, satisfaction, and acceptability^[[19]](#endnote-20)^ (timepoint unspecified), N (%):  G1*: NR (80%)  G2: NR (70%) | NR | Expulsions, N (%):  G1*: 2 (6.7%)  G2: NA  Vaginal discomfort or irritation, N (%):  G1*: NR (13%)  G2: 0 (0%)  Vaginal discharge, N (%):  G1*: NR (3%)  G2: 0 (0%)  Breakthrough bleeding, N (%):  G1*: 2 (6.7%)  G2: 6 (20%)  NS | NR | NR |

Table 1c. Risk of bias table for randomized controlled trials addressing vaginal ring acceptability

| **Author;**  **Year** | **Selection Bias** | **Performance Bias** | **Detection Bias** | **Attrition Bias** | **Reporting Bias** | **Other sources of bias** | **Summary Assessment** | **Comments** |
| --- | --- | --- | --- | --- | --- | --- | --- | --- |
|  | *Low/High/Unclear* | *Low/High/Unclear* | *Low/High/Unclear* | *%Attrition and/or % Crossover*  *Low/High/Moderate* | *Low/High/Unclear* | *Low/High/Unclear* | *Low/High/Unclear* | *Narrative if high or unclear* |
| Baeten 2016, Palanee-Phillips 2018, Mensch 2019 | Low | Low | Low | Low | Low | Low | Low | None |
| Dahiya;  2016 | Low | Low | Low | Low | Low | Low | Low | None |
| Fan;  2016 | Low | Low | Low | Low | Low | Low | Low | None |
| Hardy;  2007 | High | Low | Unclear | NA  Low | Unclear | High | High | Unclear randomization technique, no blinding of participants and assessors, vaginal ring included dose not in other groups, snowball sampling, lack of reporting on attrition |
| Hashim;  2012 | Low | Low | Low | 0 (0%) attrition  Low | Low | Low | Low | None |
| Jain;  2016 | Low | Unclear | Unclear | 2 (3.3%) attrition  Low | Low | Low | Low | None |
| Kestelyn 2018a, Kestelyn 2018b | Low | Low | Unclear | Low | Low | Low | Low | Lack of detail about outcome collection and selection of reported results. |
| Minnis 2018, Weinrib 2018, van der Straten 2018 | Low | Low | Low | 19 (6.9%) attrition  Low | Low | Low | Low | None |
| Mohamed,  2011 | Unclear | Unclear | Unclear | 19.3% attrition  Moderate | Low | Low | High | Moderate attrition and lack of blinding and detail regarding randomization procedures. |
| Nel;  2016 | Unclear | Unclear | Low | 14 (5.0%) attrition  Low | Low | Unclear | Unclear | Unclear selection, unclear randomization technique, unclear blinding of participants and assessors |
| Priya, 2016 | Low | Unclear | Unclear | Low | High | Low | High | Blinding of participants, personnel, outcome assessment not reported. Outcome data on satisfaction, acceptability reported in aggregate although multiple measures were taken. |
| Sharma,  2018 | Unclear | Unclear | Low | 14 (3.1%) attrition  Low | High | Low | Unclear | Timeframe of outcome reporting unclear, incomplete reporting of acceptability data, unclear how compliance was assessed (definition was given per cycle, reported per participant across 12 cycles). |
| Thurman; 2018 | Low | Low | Low | 1 (3.8%) attrition, DR only  Low | Unclear | Low | Low | None |
| van der Straten 2012, Montgomery 2012, Nel 2018 | Unclear | Low | Low | 13 (7.7%) attrition  Low | Low | Low | Low | Unclear selection of participants. |

Table 2a. Study characteristics and risk of bias of observational and comparative studies addressing vaginal ring acceptability, by indication

| **Author;**  **Year;**  **Country;**  **Funding;**  **Trial or study name** | **Study design;**  **Setting;**  **Study years;**  **Intervention (N group)** | **Timing of**  **acceptability assessment** (prospective / concurrent / retrospective) | **Study population (N);** | **Sample characteristics** | **Risk of bias** |
| --- | --- | --- | --- | --- | --- |
| Contraception | | | | | |
| Barreiros 2007, Guazzelli 2009;  Brazil;  Unfunded;  NR | Prospective observational study;  Gynecology clinic;  2004 to 2006;  G1*: Contraceptive vaginal ring (N=75, 62 had cycle data)  G2: COC^[[20]](#endnote-21)^ (N=75) | NA | Women who attended an educative session on pregnancy prevention at  the Gynecology Clinic of the Universidade do Oeste Paulista in the city of Presidente Prudente, Sao Paulo, Brazil, and who voluntarily opted for extended regimens of the contraceptive vaginal ring (84 days with the ring and 7-ring free days). Exclusion criteria included pregnancy and contraindications for estrogen use such as lactation, active liver disease, active thromboembolic disease, severe hypertension (blood pressure >140/100 mmHg) and diabetes mellitus, among others. Women who reported using oral or monthly injectable hormonal contraceptives during the last 3 months were also excluded, as well as those who had been using DMPA^[[21]](#endnote-22)^ injections in the 6 months prior to enrollment. | Mean age in years (SD):  G1*: 24.4 (6.4)  G2: 25.5 (7.5)  Married N (%): NR  Nulliparous N (%): NR  Rural N (%): NR | Low |
| Bitzer 2012, Egarter 2013, Bitzer, 2013;  Austria, Belgium, Czech Republic and  Slovakia, the Netherlands, Poland, Sweden, Switzerland, Israel, Russia, and Ukraine;  Merck, Sharp & Dohme;  CHOICE Study | Prospective observational study;  General practitioner, midwife, or gynecologist setting;  2009 to 2010;  Contraceptive choice before and after provider counseling (N=3,616: N=1,749 Russia, N=1,867 Ukraine) | Prospective | Women 15 to 40 years of age (in the Netherlands, women aged 16 years and older were included; in Israel, the youngest participants were 17 years old; and in Belgium, Poland, Russia and Ukraine, they were required to be at least 18 years old). Women of these ages who consulted their HCP and expressed an interest in at least one of three CHC methods (pill, patch or ring), or women who considered switching from a COC to another COC or alternative CHC, were eligible to participate in the CHOICE study. | Mean age in years (SD):  Russia: 27.3 (5.7)  Ukraine: 27.4 (5.8)  Married N (%): NR  Nulliparous N (%): NR  Rural N (%): NR | Low |
| Buckshee;  1990;  India;  NR;  NR | Prospective observational study;  All India Institute of Medical Sciences;  NR;  Contraceptive vaginal ring (N=96) | Concurrent | Healthy parous women who attended the gynecologic outpatient department at All India Institute of Medical Sciences (AIIMS) for contraception and were age 18 to 35 years, had regular menstrual cycles (28 + 7 days) for at least the last 3 cycles, regular vaginal intercourse, minimum twice a week, proven fertility with at least one living child, no hormonal contraceptive used for the last 3 months, the ability to keep a menstrual diary, and were residing within an easily travelled distance from hospital. Excluded women with known or suspected genital tract or other malignancy, genital prolapse, severe incontinence of urine, severe or chronic constipation, recurrent urinary tract infections, pelvic inflammatory disease, dyspareunia or other coital difficulties, and a previous history of idiopathic jaundice of pregnancy or history of liver disease in the past 6 months. | Mean age in years (SD): NR  Married N (%): NR  Nulliparous N (%): 0 (0%)  Rural N (%): NR | High |
| Chen;  1998;  China;  Population Council;  NR | Prospective observational study;  Two urban and suburban areas of Beijing;  NR;  G1: TCu 380A IUD (N=97)  G2*: Progesterone-releasing  vaginal ring (N=100) | Concurrent | Healthy, fully nursing women who desired to maintain breastfeeding for as long as possible; they were sexually active and between 18 and 35 years of age and had no contraindications for IUD or for vaginal ring use. | Mean age in years (SD):  G1: 26.8 (NR)  G2*: 26.5 (NR)  Married N (%): NR  Nulliparous N (%): 0 (0%)  Rural N (%): NR | Low |
| Dakhly;  2018;  Egypt;  NR;  NR | Cross-sectional survey;  Outpatient clinics at a university hospital;  2017 to 2018;  NA (N=2,128) | NA | Married women attending outpatient clinics, of all specialties, in Kasr El Aini Hospital, Cairo, who were aged 15 to 45 years and were not pregnant at the time of the survey were included. Postmenopausal or pregnant women and those who refused to participate in the survey were excluded from the study. | Mean age in years (SD): 30.75 (6.71)  Married N (%): 2,128 (100%)  Nulliparous N (%): NR  Rural N (%): NR | Low |
| Das;  2016;  India;  Self-funded;  NR | Prospective longitudinal study;  Eden Hospital, a tertiary care center in Kolkata;  NR;  Combined contraceptive vaginal ring (Nuvaring) (N=50) | Concurrent | Married women from the Department of obstetrics and gynecology, Eden  Hospital, Medical College, Kolkata, aged 18 to 45 years that have not used any form of hormonal contraceptive in the last 6 months seeking reversible means of contraception. Excluded women who are currently pregnant, those with any medical disorders such as diabetes, cardiovascular disease, severe hepatic disease, epilepsy, migraine with focal neurological symptoms, venous or arterial thrombosis, with severe pelvic inflammatory diseases, with known or suspected malignant condition of genital organ or breast, suffering from sexually transmitted diseases with undiagnosed vaginal bleeding, hypersensitivity to the active substances or any of the excipients of contraceptive vaginal rings and having irregular cycles. | Mean age in years (SD): NR  Married N (%): 50 (100%)  Nulliparous N (%): NR  Rural N (%): NR | High |
| Faundes, Hardy;  1981, 1983;  Brazil and the Dominican Republic;  International Development Research Centre, Ottawa, Canada;  NR | Observational study;  Urban and rural clinics in Santiago (n=12 clinics) and Puerto Plata (n=9), Dominican Republic, and Recife (n=2) and Campinas (n=2), Brazil;  NR;  G1*: Contraceptive vaginal ring (N=341)  G2: OCP^[[22]](#endnote-23)^ (Progestogen-only pill) (N=3146) | Concurrent | Women requesting contraceptive services at the included clinics. | Mean age in years (SD): NR  Married N (%):  G1*: NR (52%)  G2: NR (51%)  Nulliparous N (%):  G1*: NR (6%)  G2: NR (8%)  Rural N (%): NR | Unclear |
| Gupta;  1986;  India;  WHO;  NR | Observational study;  Family planning clinic;  1982 to 1983;  Vaginal ring releasing 20/ug LNG^[[23]](#endnote-24)^ daily (N=70) | Concurrent | Healthy female volunteers in the age group 18 to 35 years who came for interval contraception to the Family Planning Clinic at the Postgraduate Institute of Medical Education and Research, Chandigarh. All women had regular menstruation, proven fertility, and had had no hormonal contraception for at least 3 months. Women with genital prolapse, urinary incontinence, suspected malignancy, chronic constipation, PID dyspareunia, or recent liver disease were excluded. | Mean age in years (SD): NR  Married N (%): NR  Nulliparous N (%): NR  Rural N (%): NR | High |
| Koetswang;  1990;  NR  NR;  NR | Multicenter clinical trial;  19 centers;  1980 to 1986;  Contraceptive vaginal ring (N=1005, N=789 in LMIC: Africa [Tunisia, Zambia]: N=100, China: N=230, Asia [India, Thailand, Pakistan]: N=177, Latin America [Brazil, Colombia, Cuba]: N=282) | NA | Women between 18 and 35 years old with regular menstruation for at least the last two months, regular vaginal intercourse with minimum of twice a week, proven fertility (at least one living child or an abortion), not using hormonal contraception in intramuscular or implant form for at least three months prior to admission, not using oral contraceptives for at least one month prior to admission, and able to fill in a menstrual diary card. Women were not included if there were contraindications to the use of the vaginal ring: known or suspected genital tract, or other malignancy; genital prolapse; severe incontinence of urine; severe or chronic constipation; recurrent urinary tract infections; pelvic inflammatory disease, purulent cervical discharge, or vaginal infection; hormonal dependent cancer, or congenital disorders of renal and/or hepatic excretory function: dyspareunia or other coital difficulties; a past history of idiopathic recurrent jaundice, or pruritus of late pregnancy, or recent (within the last six months) liver disease. | Mean age in years (SD):  Africa: 27.0 (4.0)  Asia: 27.8 (3.6)  China: 29.9 (2.3)  Latin America: 26.0 (4.1)  Married N (%):NR  Nulliparous N (%):  Africa: 0 (0%)  Asia: 0 (0%)  China: 2 (0.87%)  Latin America: 12 (4.3%)  Rural N (%): NR | Low |
| Madhavan Nair;  1986;  India;  Indian Council of Medical Research;  NR | Single-group prospective observational study;  NR;  NR;  Contraceptive vaginal ring with different doses of LNG and estradiol | NA | Healthy, regularly menstruating, non-lactating women belonging to low socioeconomic group, aged 25 to 35 years. | Mean age in years (SD): NR  Married N (%): NR  Nulliparous N (%): NR  Rural N (%): NR | High |
| McLellan-Lemal;  2018;  Kenya;  CDC;  NR | Open-label single-group study;  Family planning and reproductive health clinics plus community referrals;  2014 to 2015;  Contraceptive vaginal ring (N=202) | Concurrent | Women 18 to 34 years residing within 150 km of Kisumu City, fluent in English, Swahili, or Dholuo, used DMPA or OCPs in the past 3 months, self-reported ≥2 episodes of vaginal intercourse on different days in the past 30 days, tested negative for pregnancy and HIV, no current or history of known medical contraindications for CVR use, not breastfeeding or within three months of parturition, prepared to use the CVR for six months in place of injectable or oral contraceptives. | Mean age in years (SD): NR  Married^[[24]](#endnote-25)^ N (%): 153 (77.3%)  Nulliparous^[[25]](#endnote-26)^ N (%): 14 (7.0%)  Rural N (%): NR | Low |
| Mehta;  1981;  India;  NR;  NR | Single-group pre-test post-test study;  NR;  NR;  Contraceptive vaginal ring (N=39) | Concurrent | Healthy women of proven fertility, sterilized, with history of regular cycles and indirect evidence of ovulation (N=8); healthy women of proven fertility, at least 6 months postpartum and not lactating, with at least one living child and exposed to risk of pregnancy, who opted to use contraceptive vaginal ring (N=31). | Mean age in years (SD): NR  Married N (%):NR  Nulliparous N (%): NR  Rural N (%): NR | High |
| Pandit;  2014;  India;  Organon (India) Private Ltd.;  NR | Prospective single-arm interventional study;  Six multispecialty hospitals;  December 2011 to December 2012;  NuvaRing (N=252) | Concurrent | Women (≥18 years) who were at risk of pregnancy and were seeking contraception without having any contraindication to the use of NuvaRing were enrolled in the study. | Mean age in years (SD): 29.5 (NR)  Married N (%): NR  Nulliparous N (%): 31 (14.3%)  Rural N (%): NR | Low |
| RamaRao;  2018;  Kenya, Nigeria, Senegal;  Bill & Melinda Gates Foundation;  NR | Prospective observational study;  15 family planning units within primary health centers or hospitals;  November 2013 to August 2014;  G1*: Women who chose progesterone vaginal ring (N=189; 174 participated in quantitative surveys)  G2: Women who did not choose the ring (N=174) | Prospective, concurrent | Women aged 18 to 35 years that were 6 to 9 weeks postpartum and seeking contraceptive services. | Mean age in years (SD): NR  Married or cohabitating N (%):  Kenya:  G1*: NR (98.3%)  G2: NR (93.2%)  Nigeria:  G1*: NR (100.0%)  G2: NR (100.0%)  Senegal:  G1*: NR (98.2%)  G2: NR (96.3%)  Nulliparous N (%): 0 (0%)  Rural N (%):  Kenya: G1*: NR (33.3%)  G2: NR  Nigeria:  G1*: NR (0.0%)  G2: NR  Senegal:  G1*:NR (0.0%)  G2: NR | High |
| Santibenchakula;  2016;  Thailand;  Family Planning and Reproductive Health Unit, Department of Obstetrics and Gynecology, King Chulalongkorn Memorial Hospital;  NR; | Retrospective observational cohort study;  Family planning clinic in Bangkok;  2012;  Contraceptive vaginal ring (N=39) | Concurrent | Clients who attended the Family Planning and Reproductive Health Clinic, Chulalongkorn Memorial Hospital without contraindications for using combined hormonal contraceptives, received standard physical examination, comprehensive counseling, and initiated use of the combined contraceptive vaginal ring for six cycles. | Mean age in years (SD): 29.2 (4.9)  Married^[[26]](#endnote-27)^ N (%): 39 (100%)  Nulliparous N (%): 3 (7.7%)  Rural N (%): NR | Unclear |
| Shaaban;  1991;  Egypt;  WHO, Population Council, Rockefeller Foundation;  NR | Two-group prospective study;  Assiut clinic;  NR;  G1*: Contraceptive vaginal ring (N=103)  G2: Copper IUD (N=83) | NA | Breastfeeding mothers, 5^th^ to 7^th^ week postpartum. | Mean age in years (SD): NR  Married N (%): NR  Nulliparous N (%): 0 (0%)  Rural N (%): NR | Unclear |
| Sheriar;  2014;  India;  Organon;  NR | Cross-sectional observational study;  36 sites across India;  Contraceptive choice before and after provider counseling (N=825)  G1: LNG-IUS  G2: DMPA  G3: OCP (POP^[[27]](#endnote-28)^)  G4*: Ring  G5:COC | Prospective | Sexually active women ≥18 and ≤40 years who consulted HCPs for contraception and who considered starting hormonal contraceptive method or expressed interest in switching (changing) to hormonal methods were invited to participate in the study. Women were included only if they gave informed consent and agreed and were able to fill the questionnaire. | Mean age in years (SD): 29.1 (4.8)  Married N (%): NR  Nulliparous N (%): 148 (17.9%)  Rural N (%): NR | Unclear |
| Sivin;  1981;  Brazil, Chile, Dominican Republic, Sweden, United States, Denmark/Finland, Nigeria;  PopCouncil, USAID, International Development Research Centre of Canada, the Ford Foundation, the Rockefeller Foundation, and the George J. Hecht Fund;  NR | Three-group prospective nonrandomized study;  Nine clinics;  1978 to NR;  G1*: 50 mm contraceptive vaginal ring (N=547, N=316 LMIC: Brazil [Salvador]: N=74, Brazil [Campinas]: N=77, Chile^[[28]](#endnote-29)^: N=64, Dominican Republic: N=74, Nigeria: N=27)  G2*: 58 mm contraceptive vaginal ring (N=556, N=336 LMIC: Brazil [Salvador]: N=85, Brazil [Campinas]: N=72, Chile: N=56, Dominican Republic: N=75, Nigeria: N=48)  G3: COC (Nordette) (N=533, N=356 LMIC: Brazil [Salvador] N=75, , Brazil [Campinas] N=52, Chile N=85, Dominican Republic N=75, Nigeria N=69) | Concurrent | Women within the ages of 18 and 35, regularly exposed to risk of pregnancy, not currently breastfeeding, at least one menses since last pregnancy, no injectable contraceptive in last year, and no steroid use within last 30 days. Both nulliparous and parous women were included. Medical and general criteria for subject selection and exclusion included the standard contraindications to use of oral contraceptives. | Mean age in years (SD): NR for LMIC  Married N (%): NR for LMIC  Nulliparous N (%): NR for LMIC  Rural N (%): NR for LMIC | High |
| Soni;  2013;  India;  NR;  NR | Prospective observational study;  Outpatient department in tertiary care center;  January 2010 to March 2012;  Contraceptive vaginal ring (NuvaRing) (N=184) | Concurrent | Healthy women of the age group 18 to 40 years who came to the OPD for contraceptive advice and opted for the vaginal ring. Exclusion criteria for the study were contraindications to contraceptive steroids, the presence of certain conditions relevant to ring use such as cervicitis, vaginitis, bleeding cervical erosion, dyspareunia, or other coital problems. | Mean age in years (SD): NR  Married N (%): NR  Nulliparous N (%): 90 (48.9%)  Rural N (%): NR | High |
| Weisberg;  1999;  Dominican Republic, United States, Australia;  USAID and UNFPA;  NR | Multicenter prospective study;  Centers in Sydney, Australia, Los Angeles, United States, and Santo Domingo, Dominican Republic;  NR;  Contraceptive vaginal ring (N=60, N=20 LMIC) | NA | Women aged 18 to 35 with regular menstrual cycles of 28 ± 7 days. Exclusion criteria were the usual contraindications for combined oral contraceptives. The women were required to avoid use of oral steroid medication for 4 weeks or injectables for ≥3 months before starting the study. | Mean age in years (SD): 24.8 (5.4) in LMIC  Married N (%): NR  Nulliparous N (%): 0 (0%)  Rural N (%): NR | Low |
| HIV prevention | | | | | |
| Luecke;  2016;  Uganda, South Africa, Zimbabwe;  NIH;  VOICE-D (MTN-003D) | Qualitative study following the VOICE trial;  15 sites in Uganda, South Africa,  and Zimbabwe;  2013 to 2014;  NA (prior involvement in trial comparing oral tablet to vaginal gel) (N=68) | Prospective (Hypothetical) | Former VOICE trial participants who had provided permission to be recontacted and had plasma tenofovir data. | Median age in years (SD): 28 (NR)  Married^[[29]](#endnote-30)^ N (%): 33 (49%)  Nulliparous N (%): NR  Rural N (%): NR | Unclear |
| Minnis;  2019;  Kenya, South Africa;  Bill & Melinda Gates Foundation;  TRIO | Discrete choice experiment with participants from TRIO trial and women from the same communities who did not participate in trial;  Impact Research and Development Organization in Kisumu, Kenya, and Setshaba Research Centre in Soshanguve, South Africa;  NR;  NA (N=536 total; product-experienced, N=235, and product-naïve, N=301) | Prospective (Hypothetical) | Product-experienced women had participated in the TRIO study, were 18 to 30 years, and had tried 3 placebo products—vaginal rings, oral tablets, and injections for 1 month each and then selected one to use for the subsequent 2 months. Product-naïve women were from the same communities who had not used the 3 delivery forms in the context of the TRIO Study. | Median age in years (SD): 24 (NR)  Married^[[30]](#endnote-31)^ N (%): NR (35%)  Nulliparous N (%): NR (26%)  Rural N (%): NR | Low |
| Menopause symptom management | | | | | |
| Nash;  1997;  Dominican Republic;  NR;  NR | Three-group prospective intervention study;  PROFAMILIA in Santo Domingo;  NR;  G1*: Vaginal ring releasing 100 µg estradiol/day (N=7)  G2*: Vaginal ring releasing 150 µg estradiol/day (N=7)  G3*: Vaginal ring releasing 200 µg estradiol/day (N=7) | Concurrent, retrospective | Women 3- to 15 years postmenopausal, in general good health, have experienced at least 6 months of secondary amenorrhea and have a plasma FSH level above 40 mIU/ml. They had not used estrogen therapy in the 10 days prior to pretreatment blood sampling. Exclusions were contraindications such as cancer, undiagnosed vaginal bleeding, hypertension, vaginal abnormalities or vaginal disorders, and thromboembolic disorders. | Mean age in years (SD):  G1*: 53 (4)  G2*: 54 (3)  G3*: 54 (5)  Married N (%): NR  Nulliparous N (%): NR  Rural N (%): NR | Low |
| Multipurpose prevention technology | | | | | |
| Kuteesa;  2019;  Uganda;  NIH;  HIVCOMB | Discrete choice experiment nested in an HIV-combination prevention cluster randomized pilot study;  Four fishing communities around Lake Victoria (two rural, two urban) in Mpigi, Wakiso, and Kampala districts;  December 2016 to March 2017;  NA (N=805; N=393 women) | Prospective (Hypothetical) | Members of selected community residing in the fishing community for >3 months, age 18 years and older. Excluded those who did not understand English or Luganda. | Mean age (SD)^[[31]](#endnote-32)^: NR  N (%) married^[[32]](#endnote-33)^: 162 (42%)  N (%) nulliparous: NR  N (%) rural: NR | Low |

Table 2b. Product attributes and acceptability outcomes of observational and comparative studies addressing vaginal ring acceptability

| **Author;**  **Year;**  **Country;**  **Funding;**  **Trial or study name** | **Product attributes** | **Global acceptability outcomes: N (%)** | **Secondary acceptability outcomes: N (%)** | | **Choice/use outcomes: N (%)** | **Values and preferences outcomes: N (%)** |
| --- | --- | --- | --- | --- | --- | --- |
|  |  |  | **Benefits** | **Harms** |  |  |
| Contraception | | | | | | |
| Barreiros 2007, Guazzelli 2009;  Brazil;  Unfunded;  NR | G1*:  Materials: NR (Organon, Brazil)  Dimensions: NR (Organon, Brazil)  Dose: 120 μg etonogestrel and 15 μg EE^[[33]](#endnote-34)^ daily, administered every 4 weeks, one ring-free week per 12 week period  G2:  Materials: NA  Dimensions: NA  Dose: 30 μg EE and 150 μg desogestrel, administered daily for 84 days, then 7 pill-free days | NR | NR | Guazzelli 2009: Median scheduled bleeding days per 91-day interval:  Days 1 to 91:  G1*: 4.5  G2: 4.5  Days 92 to 182:  G1*: 4.0  G2: 0  Days 183 to 273:  G1*: 4.0  G2: 3.0  Days 274 to 364:  G1*: 3.0  G2: 3.0  Total:  G1*: 16.0  G2: 15.0  Guazzelli 2009: Median scheduled bleeding/spotting days per 91-day interval:  Days 1 to 91:  G1*: 5.0  G2: 4.5  Days 92 to 182:  G1*: 4.5  G2: 4.5  Days 183 to 273:  G1*: 4.0  G2: 4.0  Days 274 to 364:  G1*: 3.0  G2: 3.0  Total:  G1*: 17.5  G2: 16.5  G1* vs G2 for total mean  scheduled bleeding/spotting for 1 year:  F=11.19, P=0.001  Guazzelli 2009: Median unscheduled bleeding days per 91-day interval:  Days 1 to 91:  G1*: 5.0  G2: 6.0  Days 92 to 182:  G1*: 3.0  G2: 4.0  Days 183 to 273:  G1*: 3.0  G2: 3.0  Days 274 to 364:  G1*: 3.0  G2: 3.0  Total:  G1*: 15.0  G2: 16.0  G1* vs G2 for total mean  unscheduled bleeding for 1 year:  F=4.85, P=0.028  Guazzelli 2009: Median unscheduled bleeding/spotting days per 91-day interval:  Days 1 to 91:  G1*: 7.0  G2: 7.5  Days 92 to 182:  G1*: 5.5  G2: 5.5  Days 183 to 273:  G1*: 3.5  G2: 4.5  Days 274 to 364:  G1*: 4.0  G2: 4.0  Total:  G1*: 21  G2: 22.5  G1* vs G2 for total mean  unscheduled bleeding/spotting for 1 year:  F=8.7, P=0.003 | Total discontinuation rate, N (%):  Barreiros 2007: G1*: 13 (17.3%)  Guazzelli 2009: G2: 14 (18.7%)  Reasons for discontinuation, N (%):  Irregular bleeding episodes:  Barreiros 2007: G1*: 3 (4.0%)  Guazzelli 2009G2: 3 (4.0%)  Amenorrhea:  Barreiros 2007: G1*: 2 (2.6%)  Guazzelli 2009G2: 2 (2.6%)  Perception of the ring by the partner:  Barreiros 2007: G1*: 2 (2.6%)  Guazzelli 2009: G2: 0 (0%)  Desire for pregnancy:  Barreiros 2007: G1*: 2 (2.6%)  Guazzelli 2009: G2: 0 (0%)  Did not return for follow-up:  Barreiros 2007: G1*: 1 (1.3%)  Guazzelli 2009: G2: 2 (2.6%)  Incorrect use:  Barreiros 2007: G1*: 1 (1.3%)  Guazzelli 2009: G2: 2 (2.6%)  Perception of the ring by the patient:  Barreiros 2007: G1*: 1 (1.3%)  Guazzelli 2009: G2: 0 (0%)  Persistent vaginal discharge:  Barreiros 2007: G1*: 1 (1.3%)  Guazzelli 2009: G2: 0 (0%)  Guazzelli 2009: Gastric intolerance:  G1*: NR  G2: 5 (6.6%) | NR |
| Bitzer 2012, Egarter 2013, Bitzer 2013;  Austria, Belgium, Czech Republic and  Slovakia, the Netherlands, Poland, Sweden, Switzerland, Israel, Russia, and Ukraine;  Merck, Sharp & Dohme;  CHOICE Study | NA | NR | NR | NR | Bitzer 2012: Method selection pre- and post-counseling:  Russia:  Pill: NR (37.6%) pre, NR (48.5%) post; 10.9 percentage point increase  P<0.0001  Patch: NR (3.0%) pre, NR (10.1%) post, 7.1 percentage point increase  P<0.0001  Ring: NR (5.3%) pre, NR (34.0%) post, 28.7 percentage point increase  P<0.0001  Other: NR (10.8%) pre, NR (3.4%) post, 7.5 percentage point decrease  P<0.0001  No preference: NR (43.3%) pre, NR (4.1%) post, 39.2 percentage point decrease  P<0.0001  Ukraine:  Pill: NR (27.4%) pre, NR (23.8%) post, 3.6 percentage point decrease  P<0.01  Patch: NR (5.8%) pre, NR (10.7%) post, 4.9 percentage point increase  P<0.0001  Ring: NR (11.3%) pre, NR (47.4%) post, 36.0 percentage point increase  P<0.0001  Other: NR (16.4%) pre, NR (7.9%) post, 8.4 percentage point decrease  P<0.0001  No preference: NR (39.2%) pre, NR (10.1%) post, 28.9 percentage point decrease  P<0.01  Bitzer 2013: Probability of switching from pill to another method: Russia (vs. Switzerland) OR=NR, NS^[[34]](#endnote-35)^; Ukraine (vs. Switzerland) OR=2.28 (95%CI 1.75, 2.98)  Bitzer 2013: Probability of switching from another method to the patch: Russia (vs. Switzerland) OR=NR, P<0.05; Ukraine (vs. Switzerland) OR=NR, P<0.05  Bitzer 2013: Probability of switching from another method to ring: Russia (vs. Switzerland) OR=NR, NS; Ukraine (vs. Switzerland) OR= 1.66 (95%CI 1.39, 1.98) | Egarter 2013: Among women who chose the ring (N=593 Russia, N=884 Ukraine):  Reasons women selected the ring, N (%):  Monthly use  Russia: 389 (65.6%)  Ukraine: 557 (63.0%)  Convenience  Russia: 451 (76.1%)  Ukraine: 674 (76.2%)  Recommended by my doctor  Russia: 427 (72.0%)  Ukraine: 610 (69.0%)  Steady, low hormone levels  Russia: 411 (69.3%)  Ukraine: 590 (66.7%)  Egarter 2013: Among women who selected daily pill (N=843 Russia, N=441 Ukraine):  Reasons for not selecting monthly ring, N (%):  Don’t like to use foreign body  Russia: 573 (68.0%)  Ukraine: 256 (58.0%)  More convenient methods are available  Russia: 386 (45.8%)  Ukraine: 164 (37.2%)  Not comfortable inserting ring in vagina  Russia: 347 (41.2%)  Ukraine: 188 (42.6%)  Don’t know anybody who uses it  Russia: 261 (31.0%)  Ukraine: 153 (34.7%)  Egarter 2013: Among women who selected weekly patch (N=176 Russia, N=199 Ukraine):  Reasons for not selecting monthly ring:  Don’t like to use foreign body  Russia: 130 (73.9%)  Ukraine: 124 (62.3%)  Not comfortable inserting ring in vagina  Russia: 83 (47.2%)  Ukraine: 99 (49.7%)  More convenient methods are available  Russia: 105 (59.7%)  Ukraine: 119 (59.8%)  Not easy to use  Russia: 66 (37.5%)  Ukraine: 78 (39.2%): |
| Buckshee;  1990;  India;  NR;  NR | Materials: Silastic  Dimensions: 55.6mm x 9.5mm  Dose: 20 μg LNG/day, used continuously for 90 days | NR | NR | Vaginal irritation, N (%): 11 (11.5%)  Repeated expulsion^[[35]](#endnote-36)^, N (%): 3 (3.1%) | “Women with cesarean sections were more likely to choose the vaginal ring as the contraceptive method than those who had vaginal deliveries”    Discontinued within 52wk due to medical reasons, N (%): 35 (36.5%)  Medical reasons for discontinuation, N (%):  Menorrhagia/metrorrhagia: 16 (16.7%)  Amenorrhea: 2 (2.1%)  Vaginal irritation: 11 (11.5%)  Repeated expulsion: 3 (3.1%)  Giddiness/tiredness: 2 (2.1%)  Ovarian cyst: 1 (1.0%)  Discontinued within 52wk due to non-medical reasons, N (%): 19 (19.8%)  Non-medical reasons for discontinuation, N (%):  Desire for pregnancy: 3 (3.1%)  Did not come for a change: 5 (5.2%)  Disliking for method: 2 (2.1%)  Involuntary pregnancy: 1 (1.0%)  Moved away: 8 (8.3%) | NR |
| Chen;  1998;  China;  Population Council;  NR | G1:  Materials: Polyethylene IUD with copper wire  Dimensions: 36mm x 32mm  Dose: NR  G2*:  Materials: Hollow Silastic ring  Dimensions: 58mm x 8.4mm  Dose: 10mg progesterone daily, ring administered every 90 days | NR | Normal bleeding by followup, N (%):  3 months  G1: NR (15.6%)  G2*: NR (4.1%)  P<0.05 6 months  G1: NR (38.2%)  G2*: NR (3.4%)  P<0.0001  9 months  G1: NR (58.0%)  G2*: NR (4.3%)  P<0.0001  12 months  G1: NR (59.0%)  G2*: NR (6.5%)  P<0.0001  Amenorrhea by followup, N (%):  3 months  G1: NR (2.1%)  G2*: NR (17.8%)  P<0.001  6 months  G1: NR (19.1%) G2*: NR (88.1%)  P<0.0001  9 months  G1: NR (7.4%) G2*: NR (80.9%)  P<0.0001  12 months  G1: NR (5.1%) G2*: NR (77.4%)  P<0.0001 | Any complaints by followup, N (%):  1 months  G1: NR (8.2%)  G2*: NR (46.0%)  P<0.001  3 months  G1: NR (5.2%)  G2*: NR (15.6%)  P<0.05  6 months  G1: NR (5.3%)  G2*: NR (16.1%)  P<0.05  9 months  G1: NR (4.5%)  G2*: NR (8.3%)  12 months  G1: NR (4.3%)  G2*: NR (12.1%)  No sexual activity by followup, N(%):  1 months  G1: NR (42.3%)  G2*: NR (41.0%)  3 months  G1: NR (36.5%)  G2*: NR (24.7%)  6 months  G1: NR (38.3%)  G2*: NR (24.2%)  9 months  G1: NR (32.6%)  G2*: NR (31.3%)  12 months  G1: NR (31.9%)  G2*: NR (30.3%) | Total discontinuation rate, N(%):  G1: 2 (2.3%)  G2*: 54 (65.4%)  Removal by reason, N(%):  Menstrual problems:  G1: 0 (0%)  G2*: 3 (6.8%)  P=0.048  Vaginal problems:  G1: 0 (0%)  G2*: 10 (13.5%)  P=0.011  Frequent ring expulsions:  G1: 0 (0%)  G2*: 7 (11.0%)  P=0.003  Ring out more than 48 hours, N(%):  G1: 0 (0%)  G2*: 12 (17.0%)  P<0.001  Unpleasant ring use:  G1: 0 (0%)  G2*: 13 (19.1%)  P<0.001  Other medical reasons:  G1: 0 (0%)  G2*: 5 (7.7%)  P=0.009  Use related problems, N(%):  G1: 1 (1.1%)  G2*: 50 (55.5%)  P<0.001  Personal reasons, N(%)  G1: 1 (1.2%)  G2*: 4 (9.9%)  P=0.072 | NR |
| Dakhly;  2018;  Egypt;  NR;  NR | Materials: NA  Dimensions: NA  Dose: NA | NR | NR | NR | Among women currently using contraceptives (N=815):  Current method used, N (%):  IUD: 413 (50.7%)  OCP: 192 (23.6%)  Injectable: 32 (3.9%)  Implant: 0 (0%)  Sterilization: 0 (0%)  Lactational amenorrhea: 34 (4.2%)  Safe period: 80 (9.8%)  Emergency contraception: 16 (2.0%)  Male condom: 48 (5.9%)  Contraceptive vaginal ring: 0 (0%)  Among women reporting past contraceptive use (N=811)  Prior method used, N (%):  IUD: 387 (47.7%)  OCP: 126 (15.5%)  Injectable: 128 (15.8%)  Implant: 16 (2.0%)  Sterilization: 0 (0%)  Lactational amenorrhea: 64 (7.9%)  Safe period: 48 (5.9%)  Emergency contraception: 17 (2.1%)  Male condom: 11 (1.4%)  Contraceptive vaginal ring: 14 (1.7%)  Current and previous contraceptive use significantly varied by level of education (no, reading and writing, primary, or secondary): P<0.001 | NR |
| Das;  2016;  India;  Self-funded;  NR | Materials: Evatane (Nuvaring)  Dimensions: 54mm x 4 mm (Nuvaring)  Dose: 15 μg EE and 120 μg etonorgestrel/day (Nuvaring), administered monthly with one ring-free week | Acceptability, N (%):  Strongly agree: NR (80%)  Agree: NR (92%)  Undecided: NR (3%) Disagree: NR (5%)  Strongly disagree: NR (2%)  Satisfied with ring usage, N (%): NR (95%)  Recommended the ring to others, N (%): NR (96%) | Easy to insert, N (%): NR (98%)  Easy to remove, N (%): NR (93%)  Ring felt by self during intercourse, N (%): NR (30%)  Ring felt by partner during intercourse, N (%): NR (18%) | Device-related adverse events^[[36]](#endnote-37)^, N (%): NR (8%)  Vaginitis, N (%): NR (2.5%)  Early withdrawal bleeding, , N (%) cycles: NR (1.2%)  Late withdrawal bleeding, , N (%) cycles: NR (13.7%)  Irregular bleeding, , N (%) cycles: NR (3.6%) | Discontinuation, N (%): 6 (NR)  Reasons for discontinuation, N:  Bleeding irregularities: 2  Coital problems: 2  Spontaneous expulsion: 1  Foreign body sensation: 1 | NR |
| Faundes 1981, Hardy 1983;  Brazil and the Dominican Republic;  International Development Research Centre (partial funding);  NR | G1*:  Materials: Silastic core, silicone rubber tubing exterior  Dimensions: 58mm  Dose: Levonorgestrel and estradiol (dose NR), administered monthly with one ring-free week  G2:  Materials: NR  Dimensions: NR  Dose: Progestogen (dose NR) | Faundes 1981: Recommended to relative, friends, and/or neighbors, N (%):  G1*: NR (62.1%)  Brazil: NR (54.5%)  Dominican Republic: NR (67.5%)  G2: NR (47.3%)  Brazil: NR (27.8%)  Dominican Republic: NR (63.4%)  Difference between G1* and G2 significant for Brazil (P<0.001) and combined locations (P<0.001)  Faundes 1981: “7 out of every 10 vaginal ring users were happy to have selected the ring.”  Faundes 1981: Among participants who did not accept method, reasons for not^[[37]](#endnote-38)^:  G1* (N=144):  Menstrual problems: 1 (0.7%)  Health problems: 16 (7.6%)  Ineffectiveness: 13 (6.3%)  Use-related problems: 95 (47.2%)  Personal: 68 (35.4%)  Others: 6 (2.8%)  G2 (N=201):  Menstrual problems: 4 (2.6%)  Health problems: 155 (62.7%) Ineffectiveness: 3 (1.0%)  Use-related problems: 9 (5.5%)  Personal: 41 (24.4%)  Others: 9 (4.0%)  Difference between G1* and G2 was significant for health problems (P<0.001), use-related problems (P<0.001), and personal (P<0.05).    Hardy 1983: Experience with method, N (%):  G1*  Very good: NR (17%)  Good: NR (47%)  Fair: NR (30%)  Bad or very bad: NR (6%)  G2  Very good: NR (7%)  Good: NR (60%)  Fair: NR (27%)  Bad or very bad: NR (6%)  Difference between G1* and G2 was significant for very good (P<0.001) and good (P<0.01). | Faundes 1981: Relatives, friends, and/or neighbors knew they used the method, N (%):  G1*: NR (87.7%)  Dominican Republic: NR (85.1%)  Brazil: NR (92.5%)  G2: NR (93.5%)  Dominican Republic: NR (89.3%)  Brazil: NR (99.0%)  Faundes 1981: Recommendation about method given to relatives, friends, and/or neighbors, N (%):  G1*(N=162):  Health: NR (16.7%)  Brazil: NR (25.0%)  Dominican Republic: NR (11.8%)  Effectiveness: NR (59.3%)  Brazil: NR (46.7%)  Dominican Republic: NR (66.7%)  Use-related: NR (24.1%)  Brazil: NR (28.3%)  Dominican Republic: NR (21.5%)  G2 (N=112):  Health: NR (26.7%)  Brazil: NR (35.5%)  Dominican Republic: NR (24.1%)  Effectiveness: NR (64.3%)  Brazil: NR (55.2%)  Dominican Republic: NR (67.5%)  Use-related: NR (8.9%)  Brazil: NR (10.3%)  Dominican Republic: NR (8.4%)  Difference between G1* and G2 significant in Dominican Republic (X^2^=9.06, P<0.02) and overall (X^2^=12.03, P<0.002).  Faundes 1981: Reasons for not recommending method to relatives, neighbors and/or friends, N (%):  G1* (N=95):  Use-related: NR (24.2%) Brazil: NR (25.0%)  Dominican Republic: NR (23.4%)  Personal: NR (75.8%)  Brazil: NR (75.0%)  Dominican Republic: NR (76.6%)  G2 (N=124):  Use-related: NR (3.2%) Brazil: NR (3.8%)  Dominican Republic: NR (2.2%)  Personal: NR (98.8%)  Brazil: NR (96.2%)  Dominican Republic: NR (97.8%)    Faundes 1981: Libido changes, N (%):  G1*:  No change: NR (24%)  Increased: NR (52%)  Decreased: NR (24%)  G2:  No change: NR (36%)  Increased: NR (39%)  Decreased: NR (25%)  Increased libido significantly higher in G1* vs G2, P<0.001  Hardy 1983: Duration of bleeding, N (%):  G1*:  Decreased: NR (46%)  No change: NR (44%)  Increased: NR (10%)  G2:  Decreased: NR (39%)  No change: NR (47%)  Increased: NR (14%)  Hardy 1983: Amount of bleeding, N (%):  G1*:  Decreased: NR (49%)  No change: NR (37%)  Increased: NR (14%)  G2:  Decreased: NR (44%)  No change: NR (44%)  Increased: NR (12%) | Faundes 1981: Partner complaints during intercourse, G1* only (N=192) , N (%):  Discomfort: 25 (13.0%)  Brazil: 3 (3.9%)  Dominican Republic: 22 (19.0%)  Discomfort at first, not any longer: 6 (3.1%)  Brazil: 2 (2.6%)  Dominican Republic: 4 (3.4%)  Intercourse is different: 3 (1.6%)  Brazil: 1 (1.3%)  Dominican Republic: 2 (1.7%)  No complaint: 158 (82.3%)  Brazil: 70 (92.1%)  Dominican Republic: 88 (75.8%)  Hardy 1983: G1* only, difficulty of insertion (N=207) , N (%):  No difficulty: NR (90%)  Too wide: NR (5%)  Pain: NR (1%)  Difficult to fold: NR (3%)  Could not see vagina: NR (1%)  Hardy 1983: G1* only, difficulty of removal, N (%):  No difficulty: NR (83%)  Pain: NR (4%)  Difficult to find: NR (6%)  Difficult to hold: NR (6%)  Subject nervous: NR (1%)  Hardy 1983: G1* only, worried about whether ring was inserted correctly, N (%): NR (43%)  Hardy 1983: G1* only, expulsion, N (%):  Never: NR (90%)  While defecating: NR (8%)  After straining: NR (1%)  While walking or sitting: NR (1%)    Hardy 1983: G1* only, problems experienced during use (N=207) , N (%):  Vaginal odor: NR (17%)  Pain: NR (33%)  Felt ring move: NR (13%)  Changed color: NR (50%)  Disliked color change: NR (32%)  Hardy 1983: G1* only, aware of ring (N=206) , N (%):  Never: NR (62%)  After or during intercourse: NR (18%)  After straining: NR (7%)  While defecating: NR (8%) While bathing: NR (2%)  When menstruating: NR (1%)  Always: NR (2%)  Hardy 1983: Vaginal discharge, N (%):  G1*:  No discharge: NR (33%) No change: NR (24%)  Improved: NR (13%)  Aggravated: NR (30%)  G2:  Hardy 1983: No discharge: NR (48%) No change: NR (16%)  Improved: NR (15%)  Aggravated: NR (21%)  Hardy 1983: Problems during use of method, N (%):  G1*:  None: NR (71%)  Menstrual: NR (10%)  Health: NR (17%)  Other: NR (2%)  G2:  None: NR (69%)  Menstrual: NR (5%)  Health: NR (26%)  Other: NR (0%) | Faundes 1981: Number that chose method, N (%):  G1*:  Santiago: 99 (2.9%)  Puerto Plata: 75 (7.6%)  Campinas: 99 (9.2%)  Recife: 68 (12.5%)  G2:  Santiago: 2024 (60.4%)  Puerto Plata: 592 (61.2%)  Campinas: 274 (25.4%)  Recife: 256 (47.1%)  Faundes 1981: Would have preferred the other method, N (%):  G1*:  Dominican Republic: NR (32.6%)  Brazil: NR (26.6%)  G2:  Dominican Republic: NR (13.2%)  Brazil: NR (11.1%)  Difference between G1* and G2 significant for Brazil (P<0.05), Dominican Republic (P<0.02), and combined locations (P<0.001)  Faundes 1981: Among those who discontinued, reasons for discontinuation, N (%):  G1* (N=155):  Menstrual problems: NR (9.0%)  Brazil: NR (13.2%)  Dominican Republic: NR (5.8%)  Health problems: NR (41.3%)  Brazil: NR (39.7%)  Dominican Republic: NR (42.5%)  Ineffectiveness: NR (1.3%)  Brazil: NR (1.5%)  Dominican Republic: NR (1.2%)  Use-related problems: NR (25.8%)  Brazil: NR (32.2%)  Dominican Republic: NR (20.7%)  Personal: NR (15.5%)  Brazil: NR (7.4%)  Dominican Republic: NR 21.8%)  Planning pregnancy: NR (7.1%)  Brazil: NR (5.9%)  Dominican Republic: NR (8.0%)  G2* (N=107):  Menstrual problems: NR (3.7%) Brazil: NR (2.3%)  Dominican Republic: NR (4.7%)  Health problems: NR (57.9%)  Brazil: NR (76.7%)  Dominican Republic: NR (45.3%)  Ineffectiveness: NR (0.9%)  Brazil: 0 (0%)  Dominican Republic: NR (1.6%)  Use-related problems: NR (2.8%)  Brazil: NR (4.7%)  Dominican Republic: NR (1.6%)  Personal: NR (14.0%)  Brazil: NR (4.7%)  Dominican Republic: NR (20.3%)  Planning pregnancy: NR (20.6%)  Brazil: NR (11.6%)  Dominican Republic: NR (26.6%)  Difference between G1* and G2 was significant for health problems (P<0.01), use-related problems (P<0.001), and planning pregnancy (P<0.001).  Faundes 1981: Removed ring for intercourse, G1* only (N=201), N (%):  Never: 153 (76.1%)  Sometimes: 27 (13.4%)  Always: 21 (10.4%)  Brazil (N=80):  Never: 61 (76.3%)  Sometimes: 8 (10.0%)  Always: 11 (13.7%)  Dominican Republic (N=121):  Never: 92 (76.0%)  Sometimes: 19 (15.7%)  Always: 10 (8.3%)  Faundes 1981: Checked if ring was in place, G1* only (N=208*), N (%): 137 (65.9%)  Brazil: 27 (33.7%)  Dominican Republic: 110 (85.9%) | Faundes 1981: Most liked characteristic after experience of use, N (%):  G1*:  None: NR (14.2%)  Brazil: NR (15.6%)  Dominican Republic: NR (13.5%)  Effectiveness: NR (10.7%)  Brazil: NR (11.7%)  Dominican Republic: NR (10.1%)  Easy to use: NR (55.6%)  Brazil: NR (44.2%)  Dominican Republic: NR (61.5%)  Health: NR (11.1%)  Brazil: NR (15.6%)  Dominican Republic: NR (8.8%)  Personal: NR (0.4%)  Brazil: 0 (0%)  Dominican Republic: NR (0.7%)  Everything: NR (8.0%)  Brazil: NR (13.0%)  Dominican Republic: NR (5.4%)  G2:  None: NR (7.5%)  Brazil: NR (12.1%)  Dominican Republic: NR (3.9%)  Effectiveness: NR (28.5%)  Brazil: NR (46.5%)  Dominican Republic: NR (15.0%)  Easy to use: NR (24.3%)  Brazil: NR (16.2%)  Dominican Republic: NR (30.7%)  Health: NR (30.1%)  Brazil: NR (20.2%)  Dominican Republic: NR (37.8%)  Personal: NR (4.9%)  Brazil: NR (4.0%)  Dominican Republic: NR (5.5%)  Everything: NR (4.4%)  Brazil: NR (1.0%)  Dominican Republic: NR (7.1%)  Difference between G1* and G2 was significant for none (P=0.025), effectiveness (P<0.001), easy to use (P<0.001), health (P<0.001), and personal (P<0.005). For Brazil, difference between G1* and G2 significant for everything (P<0.001).  Hardy 1983: Opinion on decrease in duration of menstrual bleeding during use of method, combined G1* and G2, N (%):  Liked: NR (58%)  Indifferent: NR (17%)  Disliked: NR (25%)  Hardy 1983: Opinion on decrease in amount of menstrual bleeding during use of method, combined G1* and G2, N (%):  Liked: NR (52%)  Indifferent: NR (19%)  Disliked: NR (29%)  Hardy 1983: Opinion on decrease in body weight during use of method, combined G1* and G2, N (%):  Liked: NR (27%)  Indifferent: NR (13%)  Disliked: NR (60%)  Hardy 1983: Opinion on increase in body weight during use of method, combined G1* and G2, N (%):  Liked: NR (70%)  Indifferent: NR (5%)  Disliked: NR (25%)  P<0.001 |
| Gupta;  1986;  India;  WHO;  NR | Materials: Silastic medical grade polysiloxane 382  Dimensions: 55.6mm x 9.5mm  Dose: 20 μg progesterone daily, administered every 90 days, no ring-free period | NR | NR | Any expulsions, N (%): 24 (34.3%)  Number of expulsions, N (%):  One: 19 (27.1%)  Two: 3 (4.3%)  Three: 2 (2.9%)  Discomfort to user, N (%): 4 (5.7%)  Discomfort to partner, N (%): 1 (1.4%)  Excessive vaginal discharge, N (%): 10 (14.3%) | Continuation rate: 27 (38.6%)  Discontinuation by reason, N (%):  3 months  Menstrual: 2 (2.9%)  Expulsions: 13 (18.6%)  Medical reasons: 6 (8.6%)  Non-medical reasons: 7 (10.0%)  Method failure: 3 (4.3%)  6 months  Menstrual: 3 (4.3%)  Expulsions: 2 (2.9%)  Medical reasons: 1 (1.4%)  Non-medical reasons: 1 (1.4%)  Method failure: 0 (0%)  9 months  Menstrual: 1 (1.4%)  Expulsions: 0 (0%)  Medical reasons: 0 (0%)  Non-medical reasons: 4 (5.7%)  Method failure: 1 (1.4%) | NR |
| Koetswang;  1990;  Tunisia, Zambia, England, Sweden, Switzerland, Russia, India, Thailand, Pakistan, Brazil, Colombia, Cuba;  NR;  NR | Materials: Silastic polysiloxane elastomer  Dimensions: 55.6mm x 9.5mm  Dose: ~20 μg LNG^[[38]](#endnote-39)^ per day, administered every 90 days, worn continuously | NR | NR | NR | 12-month cumulative discontinuation rate, N (%):  Africa (N=100): NR (68.1%)  Asia (N=177): NR (59.9%)  China (N=230): NR (31.7%)  Latin America (N=282): NR (57.1%)  X^2^=63, P<0.0001 | NR |
| Madhavan Nair;  1986;  India;  Indian Council of Medical Research;  NR | G1*:  Materials: NR  Dimensions: 60mm x 9mm  Dose: 57 to 60 mg LNOG and 28 to 30 mg estradiol total, administered monthly with one ring-free week^[[39]](#endnote-40)^.  G2*:  Materials: NR  Dimensions: 50mm x 9mm  Dose: 107 to 128 mg LNG and 50 to 60 mg estradiol total, administered monthly with one ring-free week^[[40]](#endnote-41)^. | NR | NR | Expulsions, G1* and G2*, N (%): 4 (22.2%) | NR | NR |
| McLellan-Lemal;  2018;  Kenya;  CDC;  NR | Materials: NR (Nuvaring)  Dimensions:  Dose: 15 μg EE and 120 μg etonorgestrel/day (Nuvaring), administered monthly | Would recommend CVR to others (agree or strongly agree), N (%): 202 (100%) | NR | More than 3 aspects of dissatisfaction, N (%): 27 (30.0%) non-adherent^[[41]](#endnote-42)^, 47 (29.0%) total, PR=1.05 (95%CI 0.78, 1.41), NS  CVR dissatisfaction aspects (N=491 aspects reported across 6 visits) N (%):  Sexual discomfort: 165 (33.6%)  Dislike CVR: 121 (24.6%)  Difficult to use: 66 (13.4%)  Non-compliance: 38 (7.7%)  Physical discomfort: 36 (7.3%)  CVR expulsion: 27 (5.5%)  Other^[[42]](#endnote-43)^: 38 (7.7%)  Among those with self-reported adherence data (N=172):  >3 dissatisfaction aspects, N (%): 13 (54.2%) non-adherent, 43 (30.5%) total, PR=2.69 (95%CI 1.39, 5.52)  Among all participants:  Side effects (N=143 side effects reported across 6 visits), N %:  Headache: 98 (68.5%)  Vaginal discharge: 22 (15.4%)  Fatigue: 15 (10.5%)  Other^[[43]](#endnote-44)^: 8 (5.6%) | Completed all 6 study visits, N (%): 142 (70.3%) | Negative attitude towards pelvic exam, N (%): 29 (20.4%) |
| Mehta;  1981;  India;  NR;  NR | Materials: Polysiloxane (PopCouncil)  Dimensions: 61mm x 9.5mm  Dose: 40 to 77 mg estradiol and 96 to 160 mg d-norgestrel, administered monthly | Participants were “satisfied with the method and were easily taught to remove and insert the ring themselves” | No discomfort during sexual intercourse with ring in place, N (%):  39 (100%) | Husband could feel ring during sex, N (%): 1 (2.6%) | Removed ring during sex, N(%): 0 (0%)  Completed trial, N (%): 22 (56.4%)  Among those that discontinued trial (n=17), reasons for discontinuation:  Non-medical reasons (N=6), delayed onset of bleeding (N=2), short cycles (N=1), scanty flow (N=1), lost ring when expelled in toilet (N=2), pelvic inflammatory disease (N=3), edema and tenderness of legs (n=1). | “Good cycle control and the personal involvement in regulating their own fertility were the main reasons quoted for acceptability” |
| Pandit;  2014;  India;  Organon (India) Private Ltd. ;  NR | Materials: NR (NuvaRing)  Dimensions: NR (NuvaRing)  Dose: NR (NuvaRing), administered monthly with one ring-free week | Very satisfied/ satisfied, N (%): 195 (94.2%)  Would recommend ring to others, N (%): 193 (93.2%) | Ring insertion was very easy/easy, N (%):  141 (69.1%) at first cycle191 (93.6%) at third cycle  Ring removal very easy/easy, N (%):  198 (97%) at first cycle  200 (98%) at second cycle  201 (98.5%) at third cycle  Never/rarely felt ring at any time, N (%):  176 (86.3%) at first cycle  193 (94.6%) at second cycle  196 (96.1%) at third cycle  Never/rarely felt ring during intercourse, N (%):  166 (81.4%) at first cycle  182 (89.2%) at second cycle  194 (95.1%) at third cycle  Partner never/rarely felt ring during intercourse, N (%):  163 (79.9%) at first cycle  177 (86.8%) at second cycle  190 (93.1%) at third cycle  Partner never objected to ring, N (%): NR (75%) | Expulsion, N (%): 3 (1.2%)  Vaginal discharge, N (%): 6 (2.4%)  Vulvovaginal pruritus, N (%): 7 (2.8%)  Discontinued due to adverse events, N (%): 17 (6.7%) | Wished to continue ring use after third cycle, N (%): 159 (76.8%)  Discontinued use after third cycle, N (%): 47 (22.7%)  Common reasons included plan for conceiving (20 [42.5%)]), expensive (10 [21.3%]), planning for permanent/long duration method of contraception (7 [14.9%]). | NR |
| RamaRao;  2018;  Kenya, Nigeria, Senegal;  Bill & Melinda Gates Foundation;  NR | G1*:  Materials: Silicone elastomer  Dimensions: 58mm x 8.4mm  Dose: 10 mg progesterone daily, administered continuously up to 3 months  G2:  Materials: NA  Dimensions: NA  Dose: NA | Among ring users who completed two cycles (N=94):  Satisfied/very satisfied with ring, N (%): NR (94.7%) 3 mo., NR (96.8%) 6 mo.  Would use ring in future, N (%): NR (90.4%) 3 mo., NR (95.7%) 6 mo.  Already recommended ring, N (%): NR (72.3%) 3 mo., NR (85.1%) 6 mo.  Would recommend ring: NR (93.6%) 3 mo., NR (97.9%) 6 mo.  Among ring users who terminated before two cycles (N=34):  Satisfied/very satisfied with ring, N (%): NR (63.0%) 3 mo., NR (100%) 6 mo.  Would use ring in future, N (%): NR (60.9%) 3 mo., NR (100%) 6 mo.  Already recommended ring: NR (52.2%) 3 mo., NR (100%) 6 mo.  Would recommend ring, N (%): NR (73.9%) 3 mo., NR (100%) 6 mo.  Family would support use of ring, N (%): NR (52.2%) 3 mo., NR (100.0%) 6 mo.  Satisfaction at 3 mo. Between ring users who completed two cycles vs terminated before two cycles P<0.01.  Among ring non-users (N=174):  Felt the ring would be acceptable to other women in community, N (%):  Kenya: NR (56%)  Nigeria: NR (86%)  Senegal: NR (69%)  P<0.01 | Among ring users who completed two cycles (N=94):  Acceptability of ring size, N (%):  Just fine: NR (51.1%) prior to use, NR (78.7%) follow-up  Too small: NR (1.1%) prior to use, NR (2.1%) follow-up  Too big: NR (45.7%) prior to use, NR (18.1%) follow-up  No opinion/neutral: NR (2.1%) prior to use, NR (1.1%) follow-up  Acceptability of ring color, N (%):  Just fine: NR (88.3%) prior to use, NR (94.7%) follow-up  Too bright: NR (9.6%) prior to use, NR (2.1%) follow-up  Too pale: NR (1.1%) prior to use, NR (2.1%) follow-up  No opinion/neutral: NR (1.1%) prior to use, NR (1.1%) follow-up  Acceptability of ring texture, N (%):  Just fine: NR (53.2%) prior to use, NR (86.2%) follow-up  Too hard: NR (10.6%) prior to use, NR (3.2%) follow-up  Too soft: NR (36.2%) prior to use, NR (9.6%) follow-up  No opinion/neutral: NR (0.0%) prior to use, NR (1.1%) follow-up  Never felt ring during sex, N (%): NR (81.5%) 3 mo., NR (68.5%) 6 mo.  Partner did not feel ring during sex, N (%):NR (69.6%) 3 mo., NR (69.5%) 6 mo.  No change in frequency of sex, N (%): NR (73.9%) 3 mo., NR (78.3%) 6 mo.  No change in sexual pleasure, N (%): NR (80.4%) 3 mo., NR (82.6%) 6 mo.  Easy to insert, N (%):NR (96.4%) baseline  Easy to remove, N (%): NR (89.3%) baseline  Easy to reinsert, among those that reinserted, N (%):NR (96.1%) baseline, NR (100%) 3 mo., NR (100%) 6 mo.  Family would support use of ring, N (%): NR (90.4%) 3 mo., NR (89.4%) 6 mo.  Among ring users who terminated before two cycles (N=34):  Acceptability of ring size, N (%):  Just fine: NR (55.9%) baseline, NR (67.7%) follow-up  Too small: NR (0.0%) baseline, NR (0.0%) follow-up  Too big: NR (44.1%) baseline, NR (23.5%) follow-up  No opinion/neutral: NR (0.0%) baseline, NR (0.0%) follow-up  Acceptability of ring color, N (%):  Just fine: NR (79.4%) baseline, NR (79.4%) follow-up  Too bright: NR (17.7%) baseline, NR (8.8%) follow-up  Too pale: NR (2.9%) baseline, NR (0.0%) follow-up  No opinion/neutral: NR (0.0%) baseline, NR (0.0%) follow-up    Acceptability of ring texture, N (%):  Just fine: NR (44.1%) baseline, NR (61.8%) follow-up  Too hard: NR (20.6%) baseline, NR (8.8%) follow-up  Too soft: NR (35.2%) baseline, NR (20.6%) follow-up  No opinion/neutral: 0 (0.0%) baseline, NR (8.8%) follow-up  Never felt ring during sex, N (%):NR (61.1%) 3 mo., NR (100.0%) 6 mo.  Partner did not feel ring during sex, N (%):NR (38.9%) 3 mo., NR (66.7%) 6 mo.  No change in frequency of sex, N (%):NR (72.2%) 3 mo., NR (66.7%) 6 mo.  No change in sexual pleasure, N (%):NR (72.2%) 3 mo., NR (66.7%) 6 mo.  Easy to insert, N (%):NR (96.4%) baseline  Easy to remove, N (%): 94.1%) baseline  Easy to reinsert, among those that reinserted, N (%): 96.1%) baseline, 100%) 3 mo., 100%) 6 mo. | Among ring users who completed two cycles (N=94):  Expulsion, N (%): NR (4.3%) 3 mo., NR (6.4%) 6 mo.  Felt ring slipping, N (%):NR (51.1%) 3 mo., NR (39.4%) 6 mo.  Among ring users who terminated before two cycles (N=34):  Expulsion, N (%):NR (17.4%) 3 mo., NR (25.0%) 6 mo.  Felt ring slipping, N (%):NR (32.6%) 3 mo., NR (75.0%) 6 mo. | Among ring non-users (N=174):  Reason for not choosing ring, N (%):  Uncomfortable inserting into vagina: NR (23%)  Could affect their health: NR (20%)  Would be uncomfortable during sex: NR (17%)  Required repeat follow-up visits: NR (15%)  Would be uncomfortable going to the toilet: NR (5%)  Would interfere with daily routine: NR (4%)  Could affect the baby's health: NR (2%)  Reasons for choosing other methods, N (%):  They or someone else had used it before: NR (37%)  Already knew about the method: NR (30%)  Felt the method was easy to use: NR (26%)  Among ring users (N=174):  Continuation: Easy/very easy to remove ring (comparator NR) aOR= 0.29 (95%CI 0.11, 0.75); Nigeria (vs. Kenya) aOR=4.68 (95%CI 0.80, 27.51); Senegal (vs. Kenya) aOR=2.30 (0.52, 10.18); Married/ cohabitating (vs. unmarried) aOR=1.96 (95%CI 0.15, 25.32); Prior use of a method (vs. no prior use) aOR=1.64 (95%CI 0.85, 3.19); Easy/very easy to insert ring (comparator NR) aOR=2.04 (95%CI 0.53, 7.82); Easy/very easy to reinsert ring (comparator NR) aOR=0.36 (95%CI 0.09, 1.53)  Adjusted for clustering within health facilities | NR |
| Santibenchakul;  2016;  Thailand;  Family Planning and Reproductive Health Unit, Department of Obstetrics and Gynecology, King Chulalongkorn Memorial Hospital;  NR | Materials: NR (NuvaRing)  Dimensions: NR (NuvaRing)  Dose: NR, given monthly | Satisfaction^[[44]](#endnote-45)^, end of 6^th^ cycle, N (%): NR (71%)  Would recommend to friends, N (%): 39 (100%) | NR | Early and continued withdrawal bleeding, N cycles (%) : 1 (<1%)  Breakthrough bleeding, N cycles (%): 5 (2%)  Breakthrough spotting, N cycles (%): 2 (1%)  Difficulty inserting or removing ring during first or second cycle, N (%): NR (12%)  Foreign body sensation after insertion, N (%): NR (~50%) (over time “these feelings gradually subsided”)  Client or partner felt ring during sexual intercourse, N (%): NR (~40%)  Affected sexual desire: "only a few"  Impact on sexual intercourse, clients, N (%): NR (>10%)  Impact on sexual intercourse, partners, N (%): NR (~12%)  Increased vaginal discharge, N (%): NR (~33%) | Continued use to 6^th^ cycle, N (%): 38 (97.4%)  N=1 discontinued because she believed her vaginitis,  leucorrhea and itching, was caused by the ring  Elected to extend use of ring after 6^th^ cycle, N (%): NR (94%) | NR |
| Shaaban;  1991;  Egypt;  WHO, Population Council, Rockefeller Foundation;  NR | G1*:  Materials: Silastic 382  Dimensions: 58.4mm x 8.8mm  Dose: 10 mg progesterone/day, administered every 90 days, worn continuously  G2:  Materials: NR (CuT-380A IUD)  Dimensions: NR (CuT-380A IUD)  Dose: NR (CuT-380A IUD) | NR | Resumption of menstruation was definitely delayed in ring users relative to IUD users (data NR) | NR | 12-month net continuation rate, N (%):  G1*: NR (66.59%)  G2: NR (85.54%)  12-month net discontinuation events (rate), by reason for discontinuation:  Pregnancy:  G1*: 1 (0.98%)  G2: 0 (1.00%)  Menstrual problems:  G1*: 0 (0%)  G2: 2 (2.42%)  Complete weaning:  G1*: 5 (4.97%)  G2: 4 (4.83%)  Severe infant illness:  G1*: 3 (2.96%)  G2: 2 (2.42%)  Failure to obtain a ring:  G1*: 3 (2.93%)  G2: 0 (0%)  Loss of ring:  G1*: 10 (9.71%)  G2: 0 (0%)  Unpleasant vaginal effects:  G1*: 2 (1.98%)  G2: 0 (0%)  Expulsion:  G1*: 0 (0%)  G2: 1 (1.21%)  Other use problem:  G1*: 0 (0%)  G2: 1 (1.21%)  Planning pregnancy:  G1*: 1 (1.01%)  G2: 0 (0%)  Separation from husband:  G1*: 1 (0.99%)  G2: 0 (0%)  Other personal reasons:  G1*: 7 (6.85%)  G2: 0 (0%)  Loss to follow-up:  G1*: 4 (3.99%)  G2: 0 (0%) | NR |
| Sheriar;  2014;  India;  Organon;  NR | G1:  Materials: NR  Dimensions: NR  Dose: NR  G2:  Materials: NR  Dimensions: NR  Dose: NR  G3:  Materials: NR  Dimensions: NR  Dose: NR  G4*:  Materials: NR  Dimensions: NR  Dose: NR  G5:  Materials: NR  Dimensions: NR  Dose: NR | NR | NR | NR | Contraceptive choice pre and post counseling, N (%):  Not decided: 260 (31.5%) pre, 30 (3.6%) post  G1: NR (3.8%) pre, NR (13.3% post  G2: NR (5.9%) pre, 112 (13.6%) post  G3: NR (1.6%) pre, 65 (7.9%) post  G4*: NR (1.8%) pre, 116 (14.1%) post  G5: 336 (30.8%) pre, NR (40.7%) post  Non-hormonal method: NR (24.6%) pre, 56 (6.8%) post  P<0.0001 for all | Pre-counseling contraceptive perceptions, N (%):  Many women use:  G1: NR (18.1%)  G2: NR (24.2%)  G3: NR (16.5%)  G4*: NR (7.2%)  G5: NR (67.0%)  Protects against certain forms of cancer:  G1:NR (10.5%)  G2: NR (7.6%)  G3: NR (6.2%)  G4*: NR (8.5%)  G5: NR (15.3%)  Gives you regular menstrual bleeding:  G1: NR (12.7%)  G2: NR (7.3%)  G3: NR (10.4%)  G4*: NR (22.2%)  G5: NR (49.0%)  Easy to forget:  G1: NR (4.7%)  G2: NR (14.5%)  G3: NR (25.7%)  G4: NR (11.6%)  G5: NR (56.8%)  Easy to use:  G1: NR (21.2%)  G2: NR (37.5%)  G3: NR (24.1%)  G4: NR (14.5%)  G5: NR (69.5%)  Can be dangerous for your health:  G1: NR (6.5%)  G2: NR (15.2%)  G3: NR (7.3%)  G4: NR (4.7%)  G5: NR (24.8%)  Many side effects:  G1: NR (12.4%)  G2: NR (25.0%)  G3: NR (16.1%)  G4: NR (6.9%)  G5: NR (38.9%)  Prevents pregnancy effectively:  G1: NR (36.5%)  G2: NR (47.9%)  G3: NR (29.8%)  G4: NR (29.3%)  G5: NR (69.3%)  Post-counseling reasons for selecting contraceptive methods, N (%):  G1:  Long acting: NR (96.4%)  Convenience: NR (76.4%)  Will not forget it: NR (74.5%)  G2:  Will not forget it: NR (70.5%)  Low chance of side effects: NR (47.3%)  G3:  Breast feeding: NR (83.1%)  Easy to use: NR (75.4%)  Convenience: NR (70.8%)  Daily use: NR (67.7%)  G4*:  Convenience: NR (75.9%)  Will not forget it: NR (74.1%)  Monthly use: NR (72.4%)  Regular menstrual bleeding: NR (64.7%)  G5:  Easy to use: NR (70.5%)  Convenience: NR (68.5%)  Regular menstrual bleeding: NR (67.9%)  Daily use: NR (60.4%)  Still effective if I experience vomiting/ diarrhea: NR (53.6%)  Method choice^[[45]](#endnote-46)^:  G1:  Many women use it (agree, vs. disagree) 1.29 (95%CI 0.86, 1.73); Starting is easy to forget (agree, vs. disagree) 1.43 (95%CI 0.71, 2.15); Starting is easy to forget (disagree, vs. agree) 0.5 (95%CI 0.06, 0.95); Many side effects (disagree, vs. agree) 1.39 (95%CI 0.93, 1.86); Regular menstrual bleeding (agree, vs. disagree) 1.46 (95%CI 0.98, 1.94); Easy to use (agree, vs. disagree) 1.53 (95%CI 1.10, 1.96); Prevents pregnancy effectively (agree, vs. disagree) 1.27 (95%CI 0.85, 1.69); Protects against certain forms of cancer (agree, vs. disagree) 0.75 (95%CI 0.19, 1.30); Protects against certain forms of cancer (disagree, vs. agree) 1.11 (95%CI 0.25, 1.96); Can be dangerous to your health (disagree, vs. agree); 1.3 (95%CI 0.87, 1.74)  G2:  Many women use it (agree, vs. disagree) 0.52 (95%CI 0.08, 0.95); Starting is easy to forget (disagree, vs. agree) 0.56 (95%CI 0.14, 0.99); Many side effects (agree, vs. disagree) -1.53 (95%CI -2.33, -0.74); Many side effects (disagree, vs. agree) 0.93 (95%CI 0.46, 1.39); Easy to use (disagree, vs. agree) -1.47 (95%CI -2.65, -0.28); Protects against certain forms of cancer (disagree, vs. agree) -1.54 (95%CI -2.96, -0.11); Can be dangerous to your health (agree, vs. disagree) -1.26 (95%CI -2.19, 0.33); Can be dangerous to your health (disagree, vs. agree) 0.69 (95%CI 0.26, 1.13)  G3:  Many side effects (agree, vs. disagree) -1.15 (95%CI -2.18, -0.11)  G4*:  Starting is easy to forget (disagree, vs. agree) 0.82 (95%CI 0.37, 1.28); Regular menstrual bleeding (agree, vs. disagree) 0.73 (95%CI 0.31 to 1.16); Easy to use (agree, vs. disagree) 0.77 (95%CI 0.33 to 1.22); Easy to use (disagree, vs. agree) -1.41 (95%CI -2.44 to 0.39); Prevents pregnancy effectively (agree, vs. disagree) 0.64 (95%CI 0.23 to 1.04); Protects against certain forms of cancer (agree, vs. disagree) 0.9 (95%CI 0.34 to 1.47); Can be dangerous to your health (disagree, vs. agree) 0.55 (95%CI 0.10 to 1.00)  G5:  Starting is easy to forget (agree, vs. disagree) -0.78 (95%CI -1.13 to 0.44); Regular menstrual bleeding (agree, vs. disagree) 0.42 (95%CI 0.13, 0.71); Many side effects (agree, vs. disagree) -0.87 (95%CI -1.19, -0.54); Easy to use (agree, vs. disagree) 0.41 (95%CI 0.05, 0.77); Easy to use (disagree, vs. agree) - 1.15 (95%CI - 1.81, -0.50); Prevents pregnancy effectively (agree, vs. disagree) 0.37 (95%CI 0.05 -0.70); Prevents pregnancy effectively (disagree, vs. agree) -1.08 (95%CI -2.07, -0.09); Protects against certain forms of cancer (agree, vs. disagree) 0.68 (95%CI 0.29, 1.06); Can be dangerous to your health (agree, vs. disagree) -1.18 (95%CI -1.58, -0.79) |
| Sivin;  1981;  Brazil, Chile, Dominican Republic, Sweden, United States, Denmark/Finland, Nigeria;  PopCouncil, USAID, International Development Research Centre of Canada, the Ford Foundation, the Rockefeller Foundation, and the George J. Hecht Fund;  NR | G1*:  Materials: Silastic ring  Dimensions: 50mm x NR  Dose: 250 µg LNG and 150 µg estradiol daily, administered monthly with one ring-free week  G2*:  Materials: Silastic ring  Dimensions: 58mm x NR  Dose: 290 µg LNG and 180 µg estradiol daily, administered monthly with one ring-free week  G3:  Materials: NA  Dimensions: NA  Dose: 150 ug of LNG and 30 ug of EE per tablet administered monthly with one pill-free week | NR | NR | NR | Continuation:  Brazil (Salvador):  G1*/G2*: 48%  G3: 47%  Brazil (Campinas):  G1*/G2*: 55%  G3: 8%  Dominican Republic:  G1*/G2*: 36%  G3: 23%  Nigeria:  G1*/G2*: 38%  G3: 27%  Chile:  G1*/G2*: 75%  G3: 58% | NR |
| Soni;  2013;  India;  NR;  NR | Materials: Ethinyl vinyl acetate (evatane)  Dimensions: 54mm x 4mm  Dose: 120 µg etonogestrel and 15 µg EE, administered monthly with one ring-free week | Would recommend ring to others, N (%): 97% | Ring easy to insert, N (%): 96%  Ring easy to remove, N (%): 98%  Partner did not object to ring during coitus, N (%): 94% | Device-related events^[[46]](#endnote-47)^, N (%): 2 (1%) (both discontinued)  Vaginitis, N (%): NR (3.8%) | Discontinuation, N (%): 10 (5.4%) 3 mo., 25 (13.6%) 12 mo./ 13 cycles  Reasons for discontinuation, N:  Device-related events: 2  Switched to LNG-IUS: 2  Satisfied with ring but relocated: 10  Desire for pregnancy: 12 | NR |
| Weisberg;  1999;  Dominican Republic, United States, Australia;  USAID and UNFPA;  NR | Materials: RTV^[[47]](#endnote-48)^ polydimethylsiloxane  Dimensions: 55mm x 9mm  Dose: NET-ac^[[48]](#endnote-49)^ and EE, administered annually with one ring-free week per month | NR | NR | Vaginal discharge, N (%): 14 (70%) | NR | NR |
| HIV prevention | | | | | |  |
| Luecke;  2016;  Uganda, South Africa, Zimbabwe;  NIH;  VOICE-D (MTN-003D) | Materials: NA  Dimensions: NA  Dose: NA | NR | NR | NR | NR | Preferred a product with long-lasting formulations (injectable, implant, vaginal ring), N (%): 55 (81%)  Preferred product formulations for HIV prevention (multiple responses possible), N (%):  Injectables: 40 (58.8%)  Implants: 37 (54.4%)  Vaginal ring: 20 (29.4%)  Oral tablets: 15 (22.1%)  Vaginal film: 12 (17.6%)  Vaginal suppository: 12 (17.6%)  Vaginal gel: 11 (16.2%)  Cervical barrier: 6 (8.82%)  Product formulation preference by demographic characteristics, N (%):  Preference for oral tablet, by country:  South Africa: 2 (23%)  Zimbabwe: 6 (25%)  Uganda: 4 (18%)  NS  Preference for oral tablet, by age:  ≤25: 8 (44%)  26+: 7 (14%)  P<0.05  Preference for oral tablet, by married or living with primary sex partner:  Yes: 4 (12%)  No: 11 (31%)  NS  Preference for oral tablet, by parity:  ≤1: 8 (35%)  2+: 7 (16%)  NS  Preference for oral tablet, by socioeconomic status:  Lowest: 5 (19%)  Middle: 8 (35%)  Highest: 2 (11%)  NS  Preference for oral tablet, by VOICE study product assignment:  Oral tablet: 8 (25%)  Vaginal gel: 7 (19%)  NS  Preference for oral tablet, by HIV serostatus:  HIV negative: 8 (15%)  HIV positive: 7 (54%)  P<0.01  Preference for vaginal gel, by country:  South Africa: 1 (5%)  Zimbabwe: 4 (17%)  Uganda: 6 (27%)  NS  Preference for vaginal gel, by age:  ≤25: 0 (0%)  26+: 11 (22%)  P<0.05  Preference for vaginal gel, by married or living with primary sex partner:  Yes: 6 (18%)  No: 5 (14%)  NS  Preference for vaginal gel, by parity:  ≤1: 1 (4%)  2+: 10 (22%)  NS  Preference for vaginal gel, by socioeconomic status:  Lowest: 7 (26%)  Middle: 3 (13%)  Highest: 1 (6%)  NS  Preference for vaginal gel, by VOICE study product assignment:  Oral tablet: 0 (0%)  Vaginal gel: 11 (31%)  P<0.001  Preference for vaginal gel, by HIV serostatus:  HIV negative: 10 (18%)  HIV positive: 1 (8%)  NS  Preference for implant, injectable, or ring, by country:  South Africa: 21 (95%)  Zimbabwe: 22 (92%)  Uganda: 12 (55%)  P<0.001  Preference for implant, injectable, or ring, by age:  ≤25: 17 (94%)  26+: 38 (76%)  NS  Preference for implant, injectable, or ring, by married or living with primary sex partner:  Yes: 28 (85%)  No: 27 (77%)  NS  Preference for implant, injectable, or ring, by parity:  ≤1: 21 (91%)  2+: 34 (76%)  NS  Preference for implant, injectable, or ring, by socioeconomic status:  Lowest: 20 (74%)  Middle: 20 (87%)  Highest: 15 (15%)  NS  Preference for implant, injectable, or ring, by VOICE study product assignment:  Oral tablet: 27 (84%)  Vaginal gel: 28 (78%)  NS  Preference for implant, injectable, or ring, by HIV serostatus:  HIV negative: 10 (18%)  HIV positive: 1 (8%)  NS  Preference for vaginal film or suppository, by country:  South Africa: 11 (51%)  Zimbabwe: 5 (21%)  Uganda: 3 (14%)  P<0.05  Preference for vaginal film or suppository, by age:  ≤25: 7 (39%)  26+: 12 (24%)  NS  Preference for vaginal film or suppository, by married or living with primary sex partner:  Yes: 4 (12%)  No: 15 (43%)  P<0.01  Preference for vaginal film or suppository, by parity:  ≤1: 11 (48%)  2+: 8 (18%)  P<0.05  Preference for vaginal film or suppository, by socioeconomic status:  Lowest: 3 (11%)  Middle: 3 (13%)  Highest: 13 (72%)  P<0.001  Preference for vaginal film or suppository, by VOICE study product assignment:  Oral tablet: 9 (28%)  Vaginal gel: 10 (28%)  NS  Preference for vaginal film or suppository, by HIV serostatus:  HIV negative: 14 (25%)  HIV positive: 5 (38%)  NS |
| Minnis;  2019;  Kenya, South Africa;  Bill & Melinda Gates Foundation;  NR | Materials: NA  Dimensions: NA  Dose: NA | NR | NR | NR | NR | Product preferences, Kenya product-experienced:  Monthly injection: NR (72%)  Monthly ring: NR (13%)  Daily pill: NR (16%)  Preferred monthly injection over monthly vaginal ring (P=0.002)  Product preferences, South Africa:  Monthly injection: NR (54%)  Monthly ring: NR (35%)  Daily pill: NR (11%)  Preferred injection every 2 to 3 months (P<0.001)  Daily oral tablet was least preferred (P<0.001).  Preferred "2-in-1" products that prevent both HIV and pregnancy (vs. HIV or prevention product only): 92%  Important to use product without partner knowledge, N (%): NR (60%)  Product choice dominated by HIV prevention efficacy, participants, N (%): 106 (20%)    Important characteristics when selecting HIV prevention product, N (%):  Pregnancy prevention: NR (80%)  Distribution location: NR (60%)  Dosage frequency (57%)  Changes in menstrual cycle: NR (50%)  Partner awareness: NR (35%)  Weight gain: NR (28%)  Most important characteristic, N (%):  Pregnancy prevention: NR (44%)  South Africa: NR (59%)  Kenya: NR (30%)  P<0.001  Distribution location: NR (22%)  Frequency of use: NR (17%)  Kenya product-naïve:  Participants for whom choice dominated by HIV prevention efficacy, N (%): 55 (36%)  South Africa:  Preferred a product with “pregnancy prevention (with lighter or regular menstruation), and no side effects” |
| Menopause symptom management/urogenital atrophy/vaginal dryness | | | | | | |
| Nash;  1997;  Dominican Republic;  NR | G1*  Materials: Silicone elastomer (FE1 Technologies)  Dimensions: 56mm x 8mm  Dose: Approx. 100 µg/day of estradtiol-17B, administered for 22 days  G2*  Materials: Silicone elastomer (FE1 Technologies)  Dimensions: 56mm x 8mm  Dose: Approx. 150 µg/day of estradtiol-17B, administered for 22 days  G3*  Materials: Silicone elastomer (FE1 Technologies)  Dimensions: 56mm x 8mm  Dose: Approx. 200 µg/day of estradtiol-17B, administered for 22 days | General experience with ring good or very good, endline, N (%): 21 (100%) | Ring size acceptable, endline, N (%): 18 (85.7%)  Withdrawal bleeding, during study, N (%):  G1*: 0 (0.0%)  G2*: 0 (0.0%)  G3*: 2 (28.6%)  Spotting after removal of ring, during study, N (%):  G1*: 1 (14.3%)  G2*: 1 (14.3%)  G3*: 1 (14.3%) | Lower abdominal pain, during study, N (%): 3 (14.3%) (groups not specified)  Spontaneous expulsion, N (%): 1 (4.8%) (group not specified)  Initial vaginal discomfort, during study, N (%):  12 (57.1%) (groups not specified) | NR | NR |
| Multipurpose prevention technology | | | | | | |
| Kuteesa;  2019;  Uganda;  NIH;  HIVCOMB | Materials: NA  Dimensions: NA  Dose: NA | NA | NA | NA | Predicted product uptake by scenario, N (%):  Scenario 1: Only oral PrEP available  Oral PrEP: NR (24.6%)  Condom: NR (12.3%)  No protection: NR (63.1%)  Scenario 2: Oral PrEP and ring available  Oral PrEP: NR (22.8%)  Condom: NR (12.3%)  Ring: NR (6.3%)  No protection: NR (58.5%)  Scenario 3: Oral PrEP, ring, injectable, implant available  Oral PrEP: NR (14.6%)  Condom: NR (12.0%)  Ring: NR (4.0%)  Injectable: NR (17.6%)  Implant: NR (14.4%)  No protection: NR (37.3%) | Product preference:  Implant (vs. oral PREP) PW^[[49]](#endnote-50)^=-0.125, NS; Injectable (vs. oral PrEP) PW=-0.223, NS; Vaginal ring (vs. oral PrEP) PW=-3.616, P<0.01.  Attribute preferences:  HIV protection PW=0.0668, P<0.01; Pregnancy prevention PW=0.710, P<0.01; STI protection PW=0.0792, NS; Ability to use in secret PW=0.131, NS; Waiting time PW=0.0498, NS; Male condom PW=11.17, P<0.01. |

Table 2c. Risk of Bias (Observational Studies)

| **Author;**  **Year** | **Potential for Confounding** | **Selection Bias** | **Bias in classification of intervention** | **Bias due to deviations from intended interventions** | **Bias due to missing data** | **Bias in measurement of outcomes** | **Bias in selection of reported result** | **Overall risk of bias** |
| --- | --- | --- | --- | --- | --- | --- | --- | --- |
|  | *Low/High/Unclear* | *Low/High/Unclear* | *Low/High/Unclear* | *Low/High/Unclear* | *Low/High/Unclear* | *Low/High/Unclear* | *Low/High/Unclear* | *Low/High/Unclear* |
| Barreiros, Guazzelli; 2007, 2009 | Low | Low | Low | Low | Low | Low | Low | Low |
| Bitzer 2012, Egarter 2013, Bitzer 2013 | Unclear | Low | Low | Low | Low | Low | Low | Low |
| Buckshee;  1990 | High | Unclear | Low | Low | Low | Low | Low | Unclear |
| Chen; 1998 | Unclear | Unclear | Low | Low | Low | Low | Low | Low |
| Faundes, Hardy;  1981, 1983 | High | High | Unclear | Unclear | Unclear | Unclear | Unclear | Unclear |
| McLellan-Lemal, 2018 | Low | Low | Low | Low | Low | Low | Low | Low |
| Minnis, 2019 | Low | High | Low | Low | Low | Low | Low | Low |
| Nash;  1997 | High | Unclear | Low | Low | Low | Unclear | Low | Low |
| Pandit;  2014 | High | Unclear | Low | Low | Low | Low | Low | Low |
| Shaaban; 1991 | Unclear | Unclear | Low | Low | Low | Unclear | Unclear | High |
| Weisberg; 1999 | Low | Unclear | Low | Low | Unclear | Low | Low | Low |

Table 2d. Risk of Bias (Uncontrolled Observational Studies)

| **Author; Year** | **Clear criteria for inclusion in the study?** | **Consecutive inclusion of patients?** | **Analyses have complete inclusion of participants (i.e., loss to follow-up)?** | **Included participants comparable?** | **All participants validly selected consistently, reliable?** | **Outcomes assessed using valid measures in a consistent, reliable way?** | **Follow-up long enough for the outcome to occur?** | **If done, statistical analyses used appropria-tely?** | **Clear reporting of participants' demographic information?** | **Clear reporting of participants' clinical information?** | **Participants described with sufficient details for others to replicate or clinicians to make inferences?** | **Rating** | **Comments** |
| --- | --- | --- | --- | --- | --- | --- | --- | --- | --- | --- | --- | --- | --- |
| Dakhly;  2018 | Yes | Unclear | Yes | Yes | Yes | Yes | NA | Yes | Yes | Yes | Yes | Low | None |
| Das;  2016 | Yes | Unclear | No | Unclear | Unclear | Unclear | Yes | NA | No | No | No | High | No information on participant demographics or clinical information, outcomes and outcomes assessment not described |
| Deng;  2017 | Yes | Unclear | Yes | Yes | Yes | Yes | Yes | Yes | Yes | No | Unclear | Unclear | None. |
| Gupta;  1986 | No | Unclear | Yes | Unclear | Unclear | Unclear | Yes | NA | No | No | No | High | No information on participant demographics or clinical information, lack of information about recruitment procedures, outcomes and outcomes assessment not described |
| Koetswang;  1990 | Yes | Unclear | Yes | Yes | Yes | Yes | Yes | Yes | Yes | Yes | Yes | Low | None |
| Kuteesa; 2019 | Yes | Unclear | Yes | Yes | Yes | Yes | NA | Yes | Yes | Yes | Yes | Low | None |
| Luecke;  2016 | Unclear | Unclear | Yes | Yes | Unclear | Unclear | NA | Yes | Yes | Yes | Unclear | Unclear | Some missing information about how participants were selected and how outcomes were assessed. |
| Madhavan Nair;  1986 | Unclear | Unclear | Unclear | Unclear | Unclear | Yes | Yes | No | No | No | No | High | Lack of detail on recruitment and demographics of participants, unclear which participants received which rings, results unclear as to whether they were presented as a proportion of participants or proportion of cycles. |
| Mehta; 1981 | Yes | Unclear | Yes | Unclear | Unclear | No | Yes | NA | No | No | No | High | Two separate groups with different inclusion criteria were enrolled and were studied for different periods of time, but results are reported together. Insufficient detail in reporting of acceptability. No participant demographic of clinical information reported. |
| RamaRao; 2018 | Yes | Unclear | Yes | Yes | Unclear | Yes | Yes | Yes | Yes | Yes | Yes | High | Lack of information about recruitment and selection of ring non-users; unclear if they were comparable to users. |
| Santiben-chakula;  2016 | Yes | Unclear | Unclear | Yes | Unclear | Yes | Yes | NA | Yes | Yes | Unclear | Unclear | Unclear if patients were chosen consecutively, whether there was any loss to follow up. |
| Sheriar,  2014 | Yes | Unclear | Yes | Unclear | Unclear | Yes | NA | No | Yes | Yes | Yes | Unclear | Lack of detail regarding participant selection/recruitment, concerns about analysis method presented in Table 4. Seems as though additional analyses to explore/control for demographic characteristics would be warranted |
| Sivin; 1981 | Yes | Unclear | Unclear | No | Unclear | Yes | Yes | NA | Yes | Yes | No | High | CVR and OCP groups non-comparable at baseline, information on recruitment incomplete, variation of baseline characteristics and most outcomes by country/site not explored |
| Soni; 2013 | Yes | Unclear | Yes | Yes | Unclear | Yes | Yes | NA | Unclear | No | No | High | Lack of detail about participant demographic information, recruitment process |

1. Demographics presented for analysis sample (N=157) [↑](#endnote-ref-2)
2. COC=Combined oral contraceptive [↑](#endnote-ref-3)
3. DRSP= Drospirenone [↑](#endnote-ref-4)
4. PBAC= pictorial blood loss assessment chart [↑](#endnote-ref-5)
5. TVF=Tenofovir [↑](#endnote-ref-6)
6. LNG=Levonorgestrel [↑](#endnote-ref-7)
7. Social harms=Nonmedical adverse consequences of study participation or dapivirine ring use [↑](#endnote-ref-8)
8. EE=Ethinyl Estradiol [↑](#endnote-ref-9)
9. Other reasons included visits outside of protocol-specified 5-day window period, consent withdrawn, missed visits, vaginal infections, not meeting trial's eligibility criteria at the crossover visit. [↑](#endnote-ref-10)
10. All reports of "problematic" outcomes were determined to be problematic for users; none were reported to be problematic for partners [↑](#endnote-ref-11)
11. COC=Combined oral contraceptive [↑](#endnote-ref-12)
12. LNG=Levonorgestrel [↑](#endnote-ref-13)
13. Compliance=For G1*, percent of women whose use did not deviate by 48 hrs. from schedule; for G2, percent of women who took all scheduled pills in a cycle [↑](#endnote-ref-14)
14. PBAC=Pictorial blood loss assessment chart [↑](#endnote-ref-15)
15. NS=Not statistically significant [↑](#endnote-ref-16)
16. Ring related events=foreign body sensation, coital problems, etc. No formal definition provided. [↑](#endnote-ref-17)
17. Liking score=1 to 5 Likert scale, higher score indicates higher acceptability [↑](#endnote-ref-18)
18. TFV=Tenofovir [↑](#endnote-ref-19)
19. Compliance=all pills taken or ring free period <48h within the 21 days of each ring use; satisfaction=call back rate <4 per month; acceptability= acceptable improvement in symptoms, incidence of side effects and whether the woman would recommend treatment to others [↑](#endnote-ref-20)
20. COC=Combined oral contraceptive [↑](#endnote-ref-21)
21. DMPA = Depot medroxyprogesterone acetate [↑](#endnote-ref-22)
22. OCP = Oral contraceptive pill [↑](#endnote-ref-23)
23. LNG=Levonorgestrel [↑](#endnote-ref-24)
24. Married or cohabitating [↑](#endnote-ref-25)
25. Defined as no live births [↑](#endnote-ref-26)
26. Married or cohabitating [↑](#endnote-ref-27)
27. POP=Progestin-only pill [↑](#endnote-ref-28)
28. Although Chile is currently classified as a high-income country by the World Bank, the country's GNI per capita at the time the study was published was USD 2,340 (source: https://data.worldbank.org/country/chile), consistent with an LMIC. [↑](#endnote-ref-29)
29. Married or cohabitating [↑](#endnote-ref-30)
30. Married or cohabitating [↑](#endnote-ref-31)
31. Demographics presented for female participants [↑](#endnote-ref-32)
32. Married and living with husband [↑](#endnote-ref-33)
33. EE=Ethinyl Estradiol [↑](#endnote-ref-34)
34. NS=Not statistically significant [↑](#endnote-ref-35)
35. Repeated expulsion=>5 expulsions in a month or >3 expulsions in a week [↑](#endnote-ref-36)
36. Device-related events included coital problems, foreign body sensation, and spontaneous expulsion; no formal definition provided [↑](#endnote-ref-37)
37. Percent is expressed as percent of those who were informed about both methods (vaginal ring and pill) during the first visit [↑](#endnote-ref-38)
38. LNG= Levonorgestrel [↑](#endnote-ref-39)
39. Occasional variation in administration noted by authors: "At times when women wanted to have longer cycles for social reasons, the rings were left in situ up to 35 days" [↑](#endnote-ref-40)
40. Occasional variation in administration noted by authors: "At times when women wanted to have longer cycles for social reasons, the rings were left in situ up to 35 days" [↑](#endnote-ref-41)
41. By pharmacy record [↑](#endnote-ref-42)
42. Other dissatisfaction aspects included lack of partner support (N=8), unclear instructions (N=5), and miscellaneous responses (N=25) [↑](#endnote-ref-43)
43. Other side effects included genital pain (N=4), depression (N=3), and elevated blood pressure (N=1) [↑](#endnote-ref-44)
44. Satisfaction defined as “satisfaction and responses to using the ring” [↑](#endnote-ref-45)
45. Binomial regression; not specified whether coefficients reported are odds or probabilities [↑](#endnote-ref-46)
46. Device-related events included vaginal discomfort, coital problems, foreign body sensation, and expulsion; no formal definition provided. [↑](#endnote-ref-47)
47. RTV= Room-Temperature-Vulcanizing [↑](#endnote-ref-48)
48. NET-ac=Norethindrone acetate [↑](#endnote-ref-49)
49. PW=Preference weight [↑](#endnote-ref-50)
