# Supplementary material for: Vaginal ring acceptability and related preferences among women in low- and middle-income countries: A systematic review and narrative synthesis
Source: PLoS One. 2019 Nov 8;14(11):e0224898. doi: 10.1371/journal.pone.0224898 (PMC6839883; doi:10.1371/journal.pone.0224898)
Supplement: S3 File — (DOCX) [file pone.0224898.s003.docx]

**S3 File. Study characteristics, acceptability outcomes, and risk of bias assessment for studies reported in conference abstracts and the grey literature**

Table 1a. Study characteristics and risk of bias of randomized controlled trials addressing vaginal ring acceptability, by indication

| **Author;**  **Year;**  **Country;**  **Funding;**  **Study name;**  **Reference type** | **Study design;**  **Setting;**  **Intervention (N group)** | **Timing of acceptability assessment**  (prospective / concurrent / retrospective) | **Key inclusion and exclusion criteria** | **Sample characteristics** | **Risk of bias** |
| --- | --- | --- | --- | --- | --- |
| HIV prevention | | | | | |
| Duby NA, van der Straten NA, Browne 2019;  Malawi, South Africa, Uganda, Zimbabwe;  National Institutes of Health;  MTN 020/ASPIRE;  Unpublished manuscripts^[[1]](#endnote-2)^ (Duby NA, van der Straten NA), Conference presentation (Browne 2019) | Phase III randomized placebo-controlled trial;  15 research sites in Malawi (Blantyre, Lilongwe), South Africa (Cape Town, Durban [7 sites], Johannesburg), Uganda (Kampala), Zimbabwe (Chitungwiza [2 sites], Harare);  G1*: Dapivirine vaginal ring (N=1,313)  G2*: Placebo vaginal ring (N=1,316)  Descriptive study with subset of G1* and G2* participants (n=214) | Prospective, concurrent | Inclusion: 18 to 45 years, able and willing to provide adequate locator information, HIV negative, sexually active (defined as having vaginal intercourse at least once in the 3 months prior to screening, using an effective method of contraception at enrollment, and intending to use an effective method for the duration of study participation), and agreeing not to participate in other research studies involving drugs, medical devices, vaginal products, or vaccines for the duration of study participation. Inclusion criteria for sub-study NR.  Exclusion: Intention to become pregnant during study; plan to relocate or travel away from the study site; pregnant; currently breastfeeding; diagnosed with a UTI, pelvic inflammatory disease, an STI or reproductive tract infection; clinically apparent Grade 2 or higher pelvic exam finding; laboratory abnormalities; or other clinical contraindications. | Mean age (SD):  Full sample:  G1*: 27.2 (6.1)  G2*: 27.3 (6.3)  Subset, G1* and G2*: 26.5 (NR)  N (%) married, G1* and G2*:  Full sample: 1,074 (41%)  Subset: 96 (45%)  N (%) nulliparous: NR  N (%) rural: NR | Low |
| Montgomery;  NA;  Zimbabwe, South Africa;  USAID, Bill and Melinda Gates Foundation;  Quatro;  Unpublished manuscript | Randomized cross-over study;  2 clinical sites in Chitungwiza, Zimbabwe, and Durban, South Africa;  G1*: Placebo monthly vaginal ring (N=200)  G2: Placebo vaginal insert (N=200)  G3: Placebo vaginal film (N=200)  G4: Placebo gel (N=200) | Prospective, concurrent | Inclusion: HIV-negative, non-pregnant, sexually active (defined as heterosexual vaginal intercourse at least 4 times per month in the past 3 months), ages 18 to 30.  Exclusion: Participation in prior HIV prevention product trials or demonstration studies. | Median age (IQR): 24 (21 to 26)  South Africa: 23 (20 to 26)  Zimbabwe: 24 (22 to 26)  N (%) married^[[2]](#endnote-3)^: NR (52%)  South Africa: NR (10%)  Zimbabwe: NR (94%)  N (%) nulliparous: NR (14%)  Zimbabwe: NR (0%)  South Africa: NR (28%)  N (%) rural: NR | Low |

Table 1b. Acceptability outcomes of randomized controlled trials and clinical trials addressing vaginal ring acceptability, by indication

| **Author;**  **Year;**  **Country;**  **Funding;**  **Study name;**  **Reference type** | **Product attributes** | **Global acceptability outcomes: N (%)** | **Secondary acceptability outcomes (Sekhet construct): N (%)** | | **Choice/use outcomes: N (%)** | **Values and preferences outcomes: N (%)** |
| --- | --- | --- | --- | --- | --- | --- |
|  |  |  | **Benefits** | **Harms** |  |  |
| HIV prevention | | | | | |  |
| Duby NA, van der Straten NA, Browne 2019;  Malawi, South Africa, Uganda, Zimbabwe;  National Institutes of Health;  MTN 020/ASPIRE;  Unpublished manuscripts^[[3]](#endnote-4)^ (Duby NA, van der Straten NA), Conference presentation (Browne 2019) | G1*:  Materials: Silicone elastomer  Dimensions: NR  Dose: 25 mg of dapivirine, administered monthly  G2*:  Materials: Silicone elastomer  Dimensions: NR (IPM)  Dose: Placebo, administered monthly | Browne 2019: Likely or very likely to use ring in future, product use end visit, N (%): NR (96%)  Browne 2019: Very likely to use ring in future, product use end visit, N (%):  Zimbabwe: NR (75%)  Uganda: NR (76%)  South Africa: NR (58%)  Malawi: NR (52%) | NR | van der Straten NA: Full sample:  General ring worry^[[4]](#endnote-5)^, baseline, N (%):  South Africa: NR (43%)  Uganda: NR (33%)  Zimbabwe: NR (6%)  Malawi: NR (8%)  P<0.001  Most common specific ring worries, baseline, N (%):  Not staying in place: NR (26%)  Coming out by accident: NR (25%)  Causing discomfort or pain during sex: NR (24%)  Coming out during sex: NR (23%)  Least common specific ring worries, baseline, N (%):  Not being liked/approved by partner: NR (4%)  Not being liked/approved by family: NR (10%)  Most common specific ring worries, 3 mo., N (%):  Not staying in place: NR (8%)  Causing health problems: NR (8%)  Coming out by accident: NR (8%)  Getting stuck inside: NR (7%)  Early study discontinuation: General ring worry at baseline (vs. no ring worry) aOR=1.58 (95%CI 1.10, 2.27)  Adjusted for country, time in study.  General ring worry at baseline: Enrolled before start of ASPIRE adherence intervention^[[5]](#endnote-6)^ (vs. enrolled after) aOR=2.26 (95%CI 1.68, 3.03)  Adjusted for country, time in study.  Browne 2019, Full sample: Unable to wear as instructed, 3 mo., N (%): NR (44%)  Felt ring during sex, 3 mo., N (%): NR (26%)  Aware of ring during normal activities, 3 mo., N (%): NR (21%)  Partner felt ring during sex, 3 mo., N (%): NR (19%)  Difficulty inserting, 3 mo., N (%): NR (15%)  Uncomfortable to wear every day, 3 mo., N (%): NR (13%)  Ring unacceptable to partner, product use end visit, N (%): NR (12%)  Minded wearing during menses, product use end visit, N (%): NR (17%)  Minded wearing during sex, product use end visit, N (%): NR (18%)  Cross-sectional non-adherence, 3 mo.:  Uncomfortable to wear every day (comparator NR): aRR^[[6]](#endnote-7)^=1.47 (95%CI NR), P<0.05  Felt ring during sex (comparator NR): aRR=1.45 (95%CI NR), P<0.05  Unable to wear as instructed (comparator NR): aRR=1.29 (95%CI NR), P<0.05  Partner felt ring during sex (comparator NR): aRR=1.29 (95%CI NR), P<0.05  Aware of ring during normal activities (comparator NR): aRR=1.28 (95%CI NR), NS^[[7]](#endnote-8)^  Difficulty inserting ring (comparator NR): aRR=1.0 (95%CI NR), NS  Adjusted for country, enrollment post-adherence support activities, months in study  Cumulative non-adherence, post 3 mo.:  Minded wearing ring during sex (comparator NR): aRR=1.68 (95%CI NR), P<0.05  Aware of ring during normal activities (comparator NR): aRR=1.55 (95%CI NR), P<0.05  Felt ring during sex (comparator NR): aRR=1.5 (95%CI NR), P<0.05  Less than very likely to use in future (comparator NR): aRR=1.36 (95%CI NR), P<0.05  Partner felt ring during sex (comparator NR): aRR=1.34 (95%CI NR), P<0.05  Minded wearing during menses (comparator NR): aRR=1.21 (95%CI NR), NS  Unable to wear as instructed (comparator NR): aRR=1.16 (95%CI NR), NS  Unacceptable to partner (comparator NR): aRR=1.16 (95%CI NR), NS  Ring uncomfortable (comparator NR): aRR=1.05 (95%CI NR), NS  Difficulty inserting (comparator NR): aRR=0.83 (95%CI NR), NS  Adjusted for country, enrollment post adherence support activities, months in study  Duby NA: Descriptive study subset:  Worried about wearing ring during menses, N (%): 25 (12%) enrollment, NR (4%) 3 mo. visit  Minded wearing ring during menses, N (%): 70 (40%)  Uganda: NR (92%)  Malawi: NR (61%)  South Africa: NR (19%)  Zimbabwe: NR (2%)  P<0.001  Expulsion during menses, N (%): 2 (1%) | Duby NA: Descriptive study subset:  Used ring in past 3 months, N (%):  3 mo. visit: 191 (89.3%)  Malawi: 37 (97.4%)  South Africa: 86 (87.8%)  Uganda: 35 (89.7%)  Zimbabwe: 33 (84.6%)  Product use end visit: 178 (83.2%)  Malawi: 30 (78.9%)  South Africa: 78 (79.6%)  Uganda: 35 (89.7%)  Zimbabwe: 35 (89.7%)    Removed ring because of menses, N (%):  3 mo. visit: 9 (5%)  Malawi: 0 (0%)  South Africa: 5 (6%)  Uganda: 4 (11%)  Zimbabwe: 0 (0%)  Product use end visit: 4 (2%)  Malawi: 0 (0%)  South Africa: 3 (4%)  Uganda: 0 (0%)  Zimbabwe: 1 (3%) | NR |
| Montgomery;  NA;  Zimbabwe, South Africa;  USAID, Bill and Melinda Gates Foundation;  Quatro;  Unpublished manuscript | G1*:  Materials: Polyurethane ring  Dimensions: NR (Particle Sciences, Inc.)  Dose: None, administered monthly, no ring-free period  G2:  Materials: NR (CoreRx)  Dimensions: NR (CoreRx)  Dose: None, administered within two hours before having sex or once per week if use during sex not an option  G3:  Materials: NR (Par Pharmaceutical)  Dimensions: NR (Par Pharmaceutical)  Dose: None, administered within two hours before having sex or once per week if use during sex not an option  G4:  Materials: Hydroxyethyl cellulose  Dimensions: NR (DPT Laboratories)  Dose: None, administered within two hours before having sex or once per week if use during sex not an option | Most preferred product, N (%):  G1*:  Enrollment, pre-video: NR (15%)  Enrollment, post-video: NR (25%)  4 mo.: NR (29%)  Pre-video vs. post-video^[[8]](#endnote-9)^: P=0.01  Pe-video vs. 4 mo.: P=0.01  G2:  Enrollment, pre-video: NR (25%)  Enrollment, post-video: NR (25%)  4 mo.: NR (26%)  Pre-video vs. post-video: NS  Pre-video vs. 4 mo.: NS  G3:  Enrollment, pre-video: NR (19%)  Enrollment, post-video: NR (17%)  4 mo.: NR (29%)  Pre-video vs. post-video: NS  Pre-video vs. 4 mo.: P<0.05  G4:  Enrollment, pre-video: NR (41%)  Enrollment, post-video: NR (33%)  4 mo.: NR (16%)  Pre-video vs. post-video: NS  Pre-video vs. 4 mo.: P<0.05  Would not consider using product in future after using each for one month, N (%):  G1*: NR (32%)  G2: NR (8%)  G3: NR (14%)  G4: NR (17%)  Least preferred product, N (%):  G1*:  Enrollment, pre-video: NR (63%)  Enrollment, post-video: NR (53%)  4 mo.: NR (42%)  Pre-video vs. post-video: P<0.05  Pre-video vs. 4 mo.: P<0.05  G2:  Enrollment, pre-video: NR (9%)  Enrollment, post-video: NR (11%)  4 mo.: NR (12%)  Pre-video vs. post-video: NS  Pre-video vs. 4 mo.: NS  G3:  Enrollment, pre-video: NR (13%)  Enrollment, post-video: NR (17%)  4 mo.: NR (23%)  Pre-video vs. post-video: NS  Pre-video vs. 4 mo.: P<0.05  G4:  Enrollment, pre-video: NR (15%)  Enrollment, post-video: NR (19%)  4 mo.: NR (23%)  Pre-video vs. post-video: NS  Pre-video vs. 4 mo.: P<0.05 | NR | Product-related adverse events, N (rate per person-months):  Vaginal discharge:  G1*: 14 (0.06)  G2: 1 (<0.01)  G3: 4 (0.02)  G4: 2 (0.01)  Intravaginal pain:  G1*: 3 (0.01)  G2: 0 (0.00)  G3: 0 (0.00)  G4: 0 (0.00)  Abnormal vaginal bleeding (spotting):  G1*: 0 (0.00)  G2: 0 (0.00)  G3: 2 (0.01)  G4: 0 (0.00) | Product choice for use in month 5, N (%):  G1*: NR (28%)  South Africa: NR (28%)  Zimbabwe: NR (29%)  G2: NR (26%)  South Africa: NR (34%)  Zimbabwe: NR (18%)  G3: NR (29%)  South Africa: NR (13%)  Zimbabwe: NR (45%)  G4: NR (16%)  South Africa: NR (25%)  Zimbabwe: NR (8%)  Used all product, crossover period, N (%):  G1*: NR (67%)  South Africa: NR (52%)  Zimbabwe: NR (81%)  G2: NR (86%)  South Africa: NR (82%)  Zimbabwe: NR (90%)  G3: NR (84%)  South Africa: NR (79%)  Zimbabwe: NR (90%)  G4: NR (87%)  South Africa: NR (78%)  Zimbabwe: NR (95%)  Used product at least once with sex, crossover period, N (%):  G1*: NR (84%)  South Africa: NR (71%)  Zimbabwe: NR (96%)  G2: NR (81%)  South Africa: NR (69%)  Zimbabwe: NR (92%)  G3: NR (80%)  South Africa: NR (64%)  Zimbabwe: NR (95%)  G4: NR (84%)  South Africa: NR (72%)  Zimbabwe: NR (95%)  Used product most or all of time with sex, choice period, N (%):  G1*: NR (88%)  South Africa: NR (77%)  Zimbabwe: NR (96%)  G2: NR (50%)  South Africa: NR (48%)  Zimbabwe: NR (53%)  G3: NR (42%)  South Africa: NR (58%)  Zimbabwe: NR (37%)  G4: NR (55%)  South Africa: NR (55%)  Zimbabwe: NR (57%)  Used product some of the time with sex, choice period, N (%):  G1*: NR (4%)  South Africa: NR (5%)  Zimbabwe: NR (4%)  G2: NR (48%)  South Africa: NR (48%)  Zimbabwe: NR (47%)  G3: NR (53%)  South Africa: NR (42%)  Zimbabwe: NR (56%)  G4: NR (31%)  South Africa: NR (27%)  Zimbabwe: NR (43%)  Choice of product for use in month 5^[[9]](#endnote-10)^:  G1*:  Ever used implants (vs. never used) aOR=2.24 (95%CI 1.08, 4.66); Zimbabwe (vs. South Africa) aOR=1.07 (95%CI 0.58, 2.06); 25 to 30 years old (vs. 18 to 24) aOR=0.95 (95%CI 0.48, 1.86); Married or living with partner (comparator NR) aOR=0.56 (95%CI 0.15, 2.12); Parous (vs. nulliparous) aOR=1.3 (95%CI 0.44, 3.85); Ever used male condoms (vs. never used) aOR=1.55 (95%CI 0.65, 3.70); Ever used injectable (vs. never used) aOR=1.08 (95%CI 0.54, 2.18); Ever used contraceptive pills (vs. never used) aOR=0.93 (95%CI 0.41, 2.12); Knew about product at enrollment (vs. did not know) aOR=2.3 (95%CI 0.59, 8.98); Told partner about product during crossover (vs. did not tell) aOR=1.11 (95%CI 0.48, 2.56); Sex during crossover month, >4 times (vs. ≤4 times) aOR=1.51 (95%CI 0.54, 4.28)  G2:  Zimbabwe (vs. South Africa) aOR= 0.43 (95%CI 0.21, 0.85); 25 to 30 years old (vs. 18 to 24) aOR= 0.61 (95%CI 0.29, 1.26); Married or living with partner (comparator NR) aOR= 1.31 (95%CI 0.37, 4.67); Parous (vs. nulliparous) aOR= 1.31 (95%CI 0.47, 3.69); Ever used male condoms (vs. never used) aOR= 0.75 (95%CI 0.30, 1.89); Ever used implants (vs. never used) aOR= 0.61 (95%CI 0.27, 1.40); Ever used injectable (vs. never used) aOR= 1.5 (95%CI 0.72, 3.12); Ever used contraceptive pills (vs. never used) aOR= 1.16 (95%CI 0.51, 2.67); Knew about product at enrollment (vs. did not know) aOR= 1.12 (95%CI 0.37, 3.35); Told partner about product during crossover (vs. did not tell) aOR= 1.06 (95%CI 0.48, 2.33); Sex during crossover month, >4 times (vs. ≤4 times) aOR= 1.55 (95%CI 0.56, 4.29)  G3:  Zimbabwe (vs. South Africa) aOR= 5.8 (95%CI 2.71, 12.40); 25 to 30 years old (vs. 18 to 24) aOR= 1.07 (95%CI 0.53, 2.16); Married or living with partner (comparator NR) aOR= 2.34 (95%CI 0.59, 9.30); Parous (vs. nulliparous) aOR= 1.65 (95%CI 0.33, 8.33); Ever used male condoms (vs. never used) aOR= 0.51 (95%CI 0.23, 1.12); Ever used implants (vs. never used) aOR= 0.69 (95%CI 0.32, 1.47); Ever used injectable (vs. never used) aOR= 0.87 (95%CI 0.41, 1.81); Ever used contraceptive pills (vs. never used) aOR= 0.94 (95%CI 0.37, 2.42); Knew about product at enrollment (vs. did not know) aOR= 0.63 (95%CI 0.26, 1.53); Told partner about product during crossover (vs. did not tell) aOR= 0.44 (95%CI 0.16, 1.23); Sex during crossover month, >4 times (vs. ≤4 times) aOR= 1.23 (95%CI 0.43, 3.52)  G4:  Zimbabwe (vs. South Africa) aOR= 0.23 (95%CI 0.09, 0.58); 25 to 30 years old (vs. 18 to 24) aOR= 2.00 (95%CI 0.85, 4.72); Married or living with partner (comparator NR) aOR= 0.55 (95%CI 0.11, 2.60); Parous (vs. nulliparous) aOR= 0.48 (95%CI 0.17, 1.37); Ever used male condoms (vs. never used) aOR= 6.35 (95%CI 0.79, 51.34); Ever used implants (vs. never used) aOR= 0.98 (95%CI 0.36, 2.70); Ever used injectable (vs. never used) aOR= 0.59 (95%CI 0.24, 1.43); Ever used contraceptive pills (vs. never used) aOR= 0.95 (95%CI 0.36, 2.50); Knew about product at enrollment (vs. did not know) aOR= 0.38 (95%CI 0.10, 1.39); Told partner about product during crossover (vs. did not tell) aOR= 1.78 (95%CI 0.69, 4.58); Sex during crossover month, >4 times (vs. ≤4 times) aOR= 0.48 (95%CI 0.16, 1.45  All models adjusted for country and crossover month of use. | NR |

Table 1c. Risk of bias table for randomized controlled trials addressing vaginal ring acceptability

| **Author;**  **Year** | **Selection Bias** | **Performance Bias** | **Detection Bias** | **Attrition Bias** | **Reporting Bias** | **Other sources of bias** | **Summary Assessment** | **Comments** |
| --- | --- | --- | --- | --- | --- | --- | --- | --- |
|  | *Low/High/Unclear* | *Low/High/Unclear* | *Low/High/Unclear* | *%Attrition and/or % Crossover*  *Low/High/Unclear* | *Low/High/Unclear* | *Low/High/Unclear* | *Low/High/Unclear* | *Narrative if high or unclear* |
| Duby NA, van der Straten NA, Browne 2019 | Low | Low | Low | Low | Low | Low | Low |  |
| Montgomery;  NA | Low | Unclear | Low | Low | Unclear | Low | Low |  |

Table 2a. Study characteristics and risk of bias of observational and comparative studies addressing vaginal ring acceptability, by indication

| **Author;**  **Year;**  **Country;**  **Funding;**  **Study name;**  **Reference type** | **Study design;**  **Setting;**  **Study years;**  **Intervention (N group)** | **Timing of acceptability assessment**  (prospective / concurrent / retrospective) | **Study population (N);** | **Sample characteristics** | **Risk of bias** |
| --- | --- | --- | --- | --- | --- |
| Contraception | | | | | |
| Merkatz;  2018;  India;  NR;  NR;  Conference abstract | Acceptability sub-study of phase III clinical trial;  NR;  Vaginal ring (N=NR) | Concurrent | Inclusion: Lactating postpartum women  Exclusion: NR | Mean age (SD): 24 (3.3)  N (%) married: NR  N (%) nulliparous: NR  N (%) rural: NR | Unclear |
| Plascencia-Nieto;  2016;  Mexico;  NR;  NR;  Conference abstract | Multicenter trial;  NR;  NR;  Influence of systematic counseling on contraceptive choice (N=1226) | Prospective | Women who received contraceptive counselling regarding the pills, vaginal ring, and contraceptive patch. | Mean age in years (SD): NR  Married N (%): NR  Nulliparous N (%): NR  Rural N (%): NR | Unclear |
| Prilepskaya;  2012;  Russia;  NR;  NR;  Conference abstract | Single-group pre-test post-test;  Physician’s office, St. Petersburg and Moscow;  NR;  Influence of provider counseling on contraceptive choice (N=1,749) | Prospective | Patients aged 18 to 40 years | Mean age in years (SD): NR  Married N (%): NR  Nulliparous N (%): NR  Rural N (%): NR | Unclear |
| RamaRao 2015, Ishaku 2015;^[[10]](#endnote-11)^  Senegal, Nigeria, Kenya;  Bill and Melinda Gates Foundation;  NR;  Reports | Prospective observational study;  15 family planning units within primary health centers and hospitals;  November 2013 to August 2014;  G1*: Women who chose progesterone vaginal ring (N=189; 174 participated in quantitative surveys)  G2: Women who did not choose the ring (N=174) | Concurrent | Women aged 18 to 35 years that were 6 to 9 weeks postpartum and seeking contraceptive services. | Mean age in years (SD): NR  Married or cohabitating N (%): Full sample: 174 (99%)  Nigeria: 58 (100%)  Nulliparous N (%): 0 (0%)  Rural N (%):  Full sample: 21 (12%)  Nigeria: 1 (1.7%) | Unclear |
| Vijayaletchumi, 2012, Siraj 2015;  2015;  Malaysia;  NR;  NR;  Conference abstracts | Cross-sectional^[[11]](#endnote-12)^;  UKM Medical Centre (UKMMC);  NR;  Observational (N=422, Siraj 2015) and sub-study among those interested in using contraceptive vaginal ring for 2 months (n=35, Vijayaletchumi 2012) | Prospective (Hypothetical) (Siraj 2015), concurrent (Vijayaletchumi 2012) | Nurses in UKM Medical Centre in Kuala Lumpur, Malaysia | Mean age in years (SD): NR  Married N (%): NR  Nulliparous N (%): NR  Rural N (%): NR | Unclear |
| Multipurpose prevention technology | | | | | |
| Ipsos 2014, Ipsos NR;  South Africa, Uganda, Nigeria;  Bill and Melinda Gates Foundation;  NR;  Report (IPSOS 2014), Summary Booklet (IPSOS NR) | Descriptive observational study of hypothetical MPT products;  NR;  NR;  G1: Injectable (N=1722)  G2: Implant (N=1722)  G3: Intra-vaginal film (N=1722)  G4*: Intra-vaginal ring (N=1722) | Prospective (Hypothetical) | NR | Mean age in years (SD): NR  Married N (%): NR  Nulliparous N (%): NR  Rural N (%): NR | Unclear |
| HIV prevention | | | | | |
| Browne;  2018;  Zimbabwe, South Africa;  USAID, Bill and Melinda Gates Foundation;  Quatro sub-study;  Conference abstract | DCE^[[12]](#endnote-13)^ with product-experienced participants of the Quatro study and product-naïve community members;  2 sites in Chitungwiza, Zimbabwe, and Durban, South Africa;  NR;  NA (DCE) (N=395) | Prospective (Hypothetical) | Inclusion: sexually active women aged 18 to 30 years.  Exclusion: NR | Median age in years (IQR): 24 (21 to 26)  N (%) married: NR N (%) nulliparous: NR (15%)  Zimbabwe: 0 (0%)  South Africa: NR (30%)  N (%) rural: NR | Unclear |
| Nassuuna 2018, Ndagire 2018;  Uganda;  NR;  NR;  Conference abstracts | Open label extension trial;  NR;  Dapivirine vaginal ring (N=121) | Concurrent | Inclusion: HIV negative, healthy  Exclusion: HIV positive, unhealthy | Mean age (SD): 32 (6)  N (%) married: NR  N (%) nulliparous: NR  N (%) rural: NR | Unclear |
| Quaife 2016, Quaife 2017;  South Africa;  USAID;  NR;  Conference Presentation (Quaife 2016), Conference Abstract (Quaife 2017) | Discrete choice experiment;  Ekhurhuleni Municipality;  2015;  G1: Diaphragm (N=661)  G2: Injection (N=661)  G3*: Vaginal ring (N=661)  G4: Microbicide gel (N=661)  G5: Oral PrEP (N=661) | Prospective (Hypothetical) | Sexually active adult males and females age 18 to 45, adolescent girls age 16 to 17, commercially active FSW^[[13]](#endnote-14)^ age 18 to 45. | Mean age in years (SD): NR  Married N (%): NR  Nulliparous N (%): NR  Rural N (%): NR | Unclear |
| Routes 2 Results;  2017;  South Africa;  MTN;  NR;  Report | Consumer product-driven market research;  South Africa;  Pre-selected areas that were non-clinical trial sites in Gauteng, Western Cape, and KwaZulu Natal;  G1*: Hypothetical ring for HIV prevention (N=1,241)  G2: Hypothetical pill for HIV prevention (N=1,241) | Prospective (Hypothetical) | Women 18 to 21 years, self-report as sexually active, never taken part in a clinical trial of a new drug. | Mean age in years (SD): NR  Married N (%): NR  Nulliparous N (%): NR  Rural N (%): NR (50%) | Unclear |

Table 2b. Product attributes and acceptability outcomes of observational and comparative studies addressing vaginal ring acceptability

| **Author;**  **Year;**  **Country;**  **Funding;**  **Study name;**  **Reference type** | **Product attributes** | **Global acceptability outcomes: N (%)** | **Secondary acceptability outcomes (Sekhet construct): N (%)** | | **Choice/use outcomes: N (%)** | **Values and preferences outcomes: N (%)** |
| --- | --- | --- | --- | --- | --- | --- |
|  |  |  | **Benefits** | **Harms** |  |  |
| Contraception | | | | | |  |
| Merkatz;  2018;  India;  NR;  NR;  Conference abstract | Materials: Silicone  Dimensions: NR  Dose: Progesterone, dose NR, administration NR | Satisfaction, N (%): NR (89%) | NR | Felt the ring while wearing it, N (%): NR (65%)  Ring expulsions more than 1 time/week, N (%): NR (18%) | Removed before sex, N (%): NR (1.5%) | NR |
| Plascencia-Nieto;  2016;  Mexico;  NR;  NR;  Conference abstract | Materials: NR  Dimensions: NR  Dose: NR | NR | NR | NR | Method choice before and after counseling, N (%):  Pill:  501 (40.9%) before, 435 (35.5%) after  P<0.001  Ring:  120 (9.8%) before, 317 (25.9%) after  P<0.001  Patch:  297 (24.3%) before, 295 (24.1%) after  Intended method choice before and after counseling:  Pill:  632 (51.6%) before, 435 (35.5%) after  P<0.001  Patch:  561 (45.8%) before, 295 (24.1%) after  P<0.001  Ring:  230 (18.8%) before, 317 (25.9%) after  P<0.001 | Reasons for choosing contraceptive ring: ease of use, monthly change, discretion (Data NR) |
| Prilepskaya;  2012;  Russia;  NR;  NR;  Conference abstract | Materials: NR  Dimensions: NR  Dose: NR | NR | NR | NR | Method choice before and after counseling, N (%):  Ring:  92 (5.3%) before, 594 (34%) after  Pill:  657 (37.6%) before, 839 (48%) after | Main reasons for choosing ring: non-daily use, stable hormone levels (Data NR) |
| RamaRao 2015, Ishaku 2015;  Senegal, Nigeria, Kenya;  Bill and Melinda Gates Foundation;  NR;  Reports | Materials: Silicone elastomer  Dimensions: 58mm x 8.4mm  Dose: 10 mg progesterone daily, administered continuously up to 3 months | NR | Ishaku 2015: Among women in Nigeria who completed study (N=47):  Ease of use outcomes by satisfaction, N (%):  Interested in using ring in future:  Satisfied: NR (92%)  Not satisfied: NR (40%)  P<0.01  Partner/family would support ring use:  Satisfied: NR (95%)  Not satisfied: NR (20%)  P<0.01  Will recommend ring to others:  Satisfied: NR (100%)  Not satisfied: NR (60%)  P<0.01  Already recommended ring to others:  Satisfied: NR (86%)  Not satisfied: NR (50%)  P=0.02  Willing to pay:  Satisfied: NR (71%)  Not satisfied: 0 (0%)  P<0.01  Easy/very easy to insert:  Satisfied: NR (100%)  Not satisfied: NR (100%)  NS^[[14]](#endnote-15)^  Easy/very easy to remove:  Satisfied: NR (93%)  Not satisfied: NR (86%)  NS  Easy/very easy to reinsert:  Satisfied: NR (100%)  Not satisfied: NR (86%)  NS  No reported side effects:  Satisfied: NR (86%)  Not satisfied: NR (60%)  NS  Ring did not fall out on its own:  Satisfied: NR (97%)  Not satisfied: NR (100%)  NS  Did not feel ring during sex:  Satisfied: NR (79%)  Not satisfied: NR (60%)  NS  Partner did not feel ring during sex:  Satisfied: NR (71%)  Not satisfied: NR (60%)  NS  No change in frequency of sex:  Satisfied: NR (84%)  Not satisfied: NR (79%)  NS  Increase in sexual pleasure:  Satisfied: NR (79%)  Not satisfied: NR (80%)  NS | NR | Ishaku 2015: Among ring users in Nigeria (N=58):  Satisfaction outcomes by study completion status:  No change or increase in frequency of sex:  Completed: NR (85%)  Terminated: 0 (0%)  P=0.03  No change or increase in sexual pleasure:  Completed: NR (81%)  Terminated: 0 (0%)  P=0.04  Easy/very easy to insert:  Completed: NR (100%)  Terminated: NR (100%)  NS  Easy/very easy to remove:  Completed: NR (89%)  Terminated: NR (100%)  NS  Easy/very easy to reinsert:  Completed: NR (96%)  Terminated: NR (100%)  NS  PVR never fell out on its own:  Completed: NR (91%)  Terminated: NR (50%)  NS  Feeling PVR <1/week or never:  Completed: NR (79%)  Terminated: NR (100%)  NS Did not feel PVR during sex: Completed: NR (79%)  Terminated: 0 (0%)  NS  Partner did not feel PVR during sex:  Completed: NR (72%)  Terminated: 0 (0%)  NS  Did not remove during sex:  Completed: NR (95%)  Terminated: NR (50%)  NS  Completed study, N (%): 47 (81.0%)  RamaRao 2015: Among women who chose ring (N=191):  Most cited reasons for choice, N (%):  User-controlled: NR (35%)  Did not have many side-effects: NR (31%)  Short-acting: NR (10%)  Did not interfere with breastfeeding, aided it: NR (6%)  Wished to try new method: NR (5%)  Ishaku 2015: Among women who chose ring in Nigeria (N=58):  Additional reasons for choosing ring, N(%):  Free: NR (47%)  RamaRao 2015: Among ring non-users (N=174):  Reasons for choosing other method, N (%):  Knew somebody who had used it: NR (48%)  Cost: NR (31%)  Prior knowledge of method: NR (29%)  Easy to use: NR (13%)  Long-acting: NR (4%) | NR |
| Vijayaletchumi, 2012, Siraj 2015;  2015;  Malaysia;  NR;  NR;  Conference abstracts | Materials: NR (NuvaRing)  Dimensions: NR (NuvaRing)  Dose: NR (NuvaRing), administered monthly | Siraj 2015 (Full sample, none used VR):  Perceived ring to be suitable contraception, N (%): 111 (26.3%)  No associations with age, marital status (Data NR)  Vijayaletchumi 2012 (Ring users): Among those who completed the trial (N=29):  Would recommend to family or friends, N (%): 29 (100%) | NR | Siraj 2015 (Full sample, none used VR):  Reasons stated for ring unsuitability, N (%):  Discomfort towards self-insertion: NR (72.2%)  Pain during sexual intercourse NR (70.2%)  Feared that their sexual partners might feel it: NR (68.9%)  Hormonal side effects: NR (62.2%)  Fear of weight gain: NR (59.7%) | Vijayaletchumi 2012 (Ring users): Among those who agreed to participate (N=34):  Discontinued^[[15]](#endnote-16)^, N (%): 5 (14.7%)  Reasons for discontinuation, N (%):  Increased vaginal discharge: 1 (20%) Uncomfortable with ring after insertion: 4 (80%)  Among those who completed the trial (N=29):  Happy to continue with ring, N (%): NR (93%) | NR |
| Multipurpose prevention technology | | | | | |  |
| Ipsos 2014, Ipsos NR;  South Africa, Uganda, Nigeria;  Bill and Melinda Gates Foundation;  NR;  Report (Ipsos 2014), Summary Booklet (Ipsos NR) | G1:  Materials: NA  Dimensions: NA  Dose: NA, administered 3-monthly  G2:  Materials: NA  Dimensions: NA  Dose: NA, 5-year contraceptive coverage and 6-month HIV protection coverage  G3:  Materials: NA  Dimensions: NA  Dose: NA  G4*:  Materials: NA  Dimensions: NA  Dose: NA | Ipsos NR: Willing to use method if all methods available, N (%):  G1: NR (28%)  South Africa: NR (27%)  Uganda: NR (35%)  Nigeria: NR (20%)  G2: NR (41%)  South Africa: NR (46%)  Uganda: NR (35%)  Nigeria: NR (44%)  G3: NR (20%)  South Africa: NR (16%)  Uganda: NR (19%)  Nigeria: NR (24%)  G4*: NR (9%)  South Africa: NR (10%)  Uganda: NR (7%)  Nigeria: NR (9%)  Ipsos NR: Willing to use method if it were the only one available, N (%):  G1: NR (71%)  South Africa: NR (77%)  Uganda: NR (81%)  Nigeria: NR (49%)  G2: NR (75%)  South Africa: NR (82%)  Uganda: NR (79%)  Nigeria: NR (64%)  G3: NR (60%)  South Africa: NR (59%)  Uganda: NR (67%)  Nigeria: NR (52%)  G4*: NR (36%)  South Africa: NR (35%)  Uganda: NR (48%)  Nigeria: NR (20%) | NR | NR | NR | Ipsos NR: Would pick a combination product over a contraceptive or an HIV prevention product alone, N (%): NR (93%)  South Africa: NR (95%)  Uganda: NR (92%)  Nigeria: NR (94%)  Ipsos 2014: Major drivers for usage and preference, N (%):  G1:  South Africa:  Good duration: NR (42%)  Trust health facilities: NR (44%)  Dal protection: NR (32%)  Discrete: NR (30%)  Uganda:  Good duration: NR (47%)  Easy to use: NR (41%)  Do not like inserting methods into vagina: NR (30%)  Dual protection: NR (15%)  Nigeria:  Do not like inserting methods into vagina: NR (54%)  Good duration: NR (33%)  Don't have to worry: NR (32%)  G2: NR  G3:  South Africa:  Easy to use: NR (43%)  Good duration: NR (29%)  Do not like injections: NR (29%)  Uganda:  Easy to use: NR (50%)  Discrete: NR (25%)  Does not have sex that often: NR (23%)  Nigeria:  Does not have sex that often: NR (36%)  Dual protection: NR (31%)  Do not like injections: NR (27%)  G4*:  South Africa:  Do not like injections: NR (31%)  Duration: NR (31%)  Dual protection: NR (29%)  Trust healthcare facilities: NR (29%)  Uganda:  Ease of use: NR (40%)  Duration: NR (23%)  Can remove it: NR (21%)  Nigeria:  Do not like injections: NR (44%)  Do not like other products: NR (35%)  Duration: NR (31%)  May want to get pregnant: NR (25%) |
| HIV prevention | | | | | | |
| Browne;  2018;  Zimbabwe, South Africa;  USAID, Bill and Melinda Gates Foundation;  Quatro sub-study;  Conference abstract | Materials: NA (DCE) Dimensions: NA (DCE)  Dose: Varied | NR | NR | NR | NR | Preferred product features:  HIV prevention efficacy (p-value NR), some vaginal wetness (P<0.001), pregnancy protection (P<0.001)  Disliked product features: Needs to be used every day (P=0.003), insertion by finger (vs. reusable applicator) (P=0.001), a lot of vaginal wetness (P<0.001).  Partner awareness of method during sex was not a significant factor in product choice (p=0.09).  Participants ranking product feature as most important to influence choice of HIV prevention product, N (%):  HIV protection efficacy: NR (67%)  Cost: NR (14%)  Mode of insertion: NR (<10%)  Location where product is collected: NR (<10%)  When you insert it: NR (<5%)  Whether it causes wetness: NR (<5%)  Whether it prevents pregnancy: NR (<10%)  Whether partner notices it during sex: NR (<10%)  Participants ranking product feature as least important to influence choice of HIV prevention product, N (%):  Whether partner notices it during sex: NR (25%)  Whether it causes wetness: NR (19%)  Cost: NR (16%)  Location where product is collected: NR (16%)  When you insert it: NR (~10%)  How you insert it: NR (~10%)  Whether it prevents pregnancy: NR (~5%)  HIV protection efficacy: NR (<5%) |
| Nassuuna 2018, Ndagire 2018;  Uganda;  NR;  NR;  Conference abstract | Materials: NR  Dimensions: NR  Dose: Dapivirine, dose NR, administered three-monthly | NR | Nassuuna 2018: Felt they could support themselves to change ring on time, 1 mo., N (%): 37 (30.6%)  Ndagire 2018: Disclosed trial participation to male partner, N (%): 46 (57%) | NR | Nassuuna 2018: Optimally supported self (changed ring on time), N (%): 64 (52.9%) 3 mo., 90 (74.4%) 6 mo.  Nassuuna 2018: Among those that needed support to change ring (n=11):  Reasons for needing support, N (%):  Busy: 6 (54.5% )  Had not disclosed to partner, could not use reminder tools^[[16]](#endnote-17)^: 3 (27.3%)  No reason: 2 (18.2%)  Ndagire 2018: Ring removals due to partner influence, N (%): 10 (34%) | NR |
| Quaife 2016, Quaife 2017;  South Africa;  USAID;  NR;  Conference Presentation (Quaife 2016), Conference Abstract (Quaife 2017) | G1:  Materials: NA  Dimensions: NA  Dose: NA  G2:  Materials: NA  Dimensions: NA  Dose: NA  G3*: Materials: NA  Dimensions: NA  Dose: NA  G4:  Materials: NA  Dimensions: NA  Dose: NA  G5:  Materials: NA  Dimensions: NA  Dose: NA | NR |  | NR | Quaife 2017: Predicted uptake by population group (Data NR):  Adolescent women:  G1: ~2% just HIV protection, ~5% HIV and pregnancy protection, ~7% HIV pregnancy and STI protection  G2: ~4% just HIV protection, ~10% HIV and pregnancy protection, ~13% HIV pregnancy and STI protection  G3*:~1% just HIV protection, ~3% HIV and pregnancy protection, ~4% HIV pregnancy and STI protection  G4: ~2% just HIV protection, ~7% HIV and pregnancy protection, ~10% HIV pregnancy and STI protection  G5: ~2% just HIV protection, ~7% HIV and pregnancy protection, ~9% HIV pregnancy and STI protection  Adult women:  G1: ~3% just HIV protection, ~4% HIV and pregnancy protection, ~5% HIV pregnancy and STI protection  G2: ~12% just HIV protection, ~14% HIV and pregnancy protection, ~17% HIV pregnancy and STI protection  G3*:~3% just HIV protection, ~4% HIV and pregnancy protection, ~5% HIV pregnancy and STI protection  G4: ~3% just HIV protection, ~3% HIV and pregnancy protection, ~4% HIV pregnancy and STI protection  FSW^[[17]](#endnote-18)^:  G1: ~2% just HIV protection, ~2% HIV and pregnancy protection, ~2% HIV pregnancy and STI protection  G2: ~17% just HIV protection, ~18% HIV and pregnancy protection, ~20% HIV pregnancy and STI protection  G3*:~5% just HIV protection, ~5% HIV and pregnancy protection, ~6% HIV pregnancy and STI protection  G4: ~3% just HIV protection, ~3% HIV and pregnancy protection, ~3% HIV pregnancy and STI protection  G5: ~4% just HIV protection, ~4% HIV and pregnancy protection, ~5% HIV pregnancy and STI protection | Quaife 2017: Product preference rankings: Injectable (highest), microbicide gel, diaphragm, oral PrEP, vaginal ring (lowest)  Quaife 2017: Population group with highest preference for each product (Data NR):  G1: Adolescent females  G2: FSW  G3*: Adult females  G4: Adolescent females  G5: Adolescent females  Quaife 2017: Preferred product attributes, variation by participant group (Data NR):  STI protection: Lowest for adult females, highest for FSW  Pregnancy prevention: Lowest for adult females, highest for adolescent females  HIV protection: lowest for adult females, highest for adult males  Quaife 2016: Ring preference lower among adolescent girls: NR  P=0.01  Quaife 2016: Differences in attribute preferences by population group:  HIV protection, FSW vs. adult women: PWR^[[18]](#endnote-19)^=1.87  P=0.04  HIV protection FSW vs. adolescent girls: PWR: 2.14  P=0.02 |
| Routes 2 Results;  2017;  South Africa;  MTN;  NR;  Report | G1*:  Materials: NA  Dimensions: NA  Dose: NA  G2:  Materials: NA  Dimensions: NA  Dose: NA | Most common emotional reactions to product after hearing short description of product, N (%):  G1*:  Shocked: NR (32%)  Happy: NR (31%)  Amazed: NR (23%)  Safe: NR (23%)  Frightened: NR (22%)  Excited: NR (22%)  G2:  Happy: NR (53%)  Safe: NR (33%)  Excited: NR (30%)  Amazed: NR (24%)  Confident: NR (20%)  Protected: NR (20%)  Interested or very interested in finding out more about product, N (%):  G1*: NR (57%)  G2: NR (76%)  Willing to try product, N (%):  G1*:  Yes: NR (44%)  Not sure: NR (25%)  No: NR (31%)  G2:  Yes: NR (67%)  No: NR (22%)  Not sure: NR (12%)  How product makes participants feel, N (%):  Safe:  G1*: NR (65%)  G2: NR (58%)  Protected:  G1*: NR (59%)  G2: NR (54%)  Healthy:  G1*: NR (38%)  G2: NR (31%)  Happy:  G1*: NR (28%)  G2: NR (26%)  Confident:  G1*: NR (24%)  G2: NR (25%)  Proud:  G1*: NR (21%)  G2: NR (18%)  Free:  G1*: NR (20%)  G2: NR (21%)  Comfortable: G1*: NR (19%)  G2: NR (24%)  In control:  G1*: NR (17%)  G2: NR (22%)  Independent:  G1*: NR (11%)  G2: NR (11%) | Change in product opinion after viewing full product profile, N (%):  G1*:  Improved: NR (53%)  Stayed the same: NR (26%)  Worsened: NR (21%)  G2:  Improved: NR (75%)  Stayed the same: NR (17%)  Worsened: NR (8%)  Among those with improved opinion after viewing full product profile (N=656 G1*, N=925 G2)  Main reasons for improved opinion, N (%):  G1*:  Benefits of HIV prevention: NR (31%)  Reassurance about safety/side effects: NR (24%)  Additional information: NR (18%)  Good duration of use: NR (13%)  G2:  Reassurance about safety/ side effects: NR (23%)  Product will protect people: NR (19%)  Benefits of HIV prevention: NR (18%)  Familiar with format: NR (16%)  Additional information: NR (16%)  Perceived benefits, N (%):  G1*:  Proven to be safe: NR (35%)  Tested in 2,500 women: NR (32%)  Will not harm baby if become pregnant: NR (25%)  Few side effects: NR (23%)  Does not increase size of vagina: NR (31%)  Not painful/cannot be seen: NR (29%)  Easy to insert and remove at home/ comes in one size: NR (28%)  The ring stays in at all times/ monthly use: NR (25%)  In order to work, the ring is used continually for 30 days/ periods will not stop: NR (24%)  No need for cleaning: NR (23%)  It is inserted into the vagina/ a HCP is not required for insertion or removal: NR (22%)  G2:  Proven to be safe: N R (51%)  Tested in 566 women in Kenya and Uganda: NR (41%)  Provided by healthcare providers: NR (34%)  It will not harm your baby if you become pregnant: NR (33%)  Manageable side effects: NR (28%)  It is easy to take at home/ you can choose the time of day you take the pill: NR (34%)  It can be taken alongside other medication/ vitamins: NR (34%)  30 days' worth of pills is convenient/ daily use/ familiarity: NR (31%)  It does not need to be refrigerated: NR (25%)  Do not have to tell partner/ child-proof packaging: NR (24%) | Concerns about comfort, N (%):  G1*: NR (~30%)  G2: NR  Among those with worsened opinion after viewing full product profile (N=260 G1*, N=104 G2):  Main reasons for worsened opinions, N (%):  G1*:  Looks uncomfortable/ painful: NR (52%)  Concerns about something in vagina: NR (21%)  G2:  Concerns about safety/ side effects: NR (44%)  Too big/ difficult to swallow: NR (23%)  Dislike taking pills: NR (20%)  Perceived drawbacks, N (%):  G1*:  It might be painful or uncomfortable: NR (33%)  Partner may feel it during sex: NR (31%)  Does not protect against other STIs: NR (27%)  It might increase the size of your vagina: NR (25%)  It is inserted into the vagina / It might be too big: NR (24%)  Unfamiliarity: NR (23%)  It might become unclean during your period: NR (22%)  Not sure that it is safe: NR (22%)  G2:  Concern about possible impact to kidneys: NR (36%)  Concern about vomiting and sickness: NR (33%)  Does not protect against other STIs: NR (29%)  Concern about diarrhea: NR (28%)  Can easily forget to take the pill: NR (28%)  Potential of side effects (general): NR (26%)  The pill looks too big and may be difficult to swallow: NR (26%)  Does not protect against pregnancy // Concern about protection if you forget to take the pill: NR (26%)  Could be mistaken for an ARV: NR (22%) | NR | Interest in dual prevention product, N (%):  Both HIV and STI prevention in same product: NR (94%)  Both pregnancy and HIV prevention in same product: NR (91%)  Daily HIV prevention pill and daily contraceptive pill, packaged together: NR (90%)  Attitudes towards HIV prevention and sexual health, N (%):  Want to enjoy safe sex: NR (88%)  Protecting sexual health is a priority: NR (86%)  Sexual health is an important part of overall health: NR (85%)  Protection from HIV is important: NR (92%)  G1* and G2, attitudes towards products generally, N (%):  Important that products do not look too medical: NR (71%)  Need more before feel comfortable enough to try: NR (85%)  Unwilling to try products if efficacy is 30-50%: NR (60%)  Willing to try if efficacy is 90%: NR (≥50%)  Would consider trying preferred product if free: NR (92%)  Product-agnostic reasons behind willingness to use products, N (%):  I will be protected:  G1*: NR (55%)  G2: NR (54%)  I will be healthy:  G1*: NR (34%)  G2: NR (37%)  Proven to be safe:  G1*: NR (22%)  G2: NR (32%)  It will give me control:  G1*: NR (18%)  G2: NR (19%)  Tested among 2500/566 women:  G1*: NR (16%)  G2: NR (19%)  Protect my family:  G1*: NR (12%)  G2: NR (18%)  I won't be afraid:  G1*: NR (15%)  G2: NR (16%)  Among those willing to try method (N=550 G1*, N=828 G2):  Main product-specific reasons behind willingness to try, N (%):  G1*:  I would forget to take pills: NR (57%)  I dislike taking pills: NR (55%)  Side effects are less severe than daily pills: NR (40%)  I prefer to use a product every month than every day: NR (23%)  It is more likely that pills will be seen by others: NR (22%)  I am confident I can learn to insert it: NR (22%)  It cannot be seen: NR (13%)  It does not look like a traditional medicine: NR (13%)  Monthly use: NR (11%)  Few side effects: NR (10%)  Easy home insertion/ removal: NR (10%)  It is not painful: NR (10%)  It comes in one size: NR (9%)  G2:  More familiar and comfortable taking pills: NR (55%)  I do not like the idea of insertion: NR (43%)  I dislike the form of the monthly ring: NR (38%)  My partner may feel the ring: NR (27%)  I am confident I will remember/ use reminders: NR (22%)  It is familiar: NR (14%)  It is easy to take at home: NR (14%)  I prefer to take a product daily than monthly: NR (13%)  30 days worth is convenient: NR (12%)  Daily use: NR (11%)  It can be taken alongside other medicines and vitamins: NR (8%)  Among those unwilling to try method (N=691 G1*, N=413 G2):  Main reasons behind unwillingness to try, N (%):  G1*:  Need more information from experienced user: NR (23%)  It might be painful or uncomfortable: NR (25%)  It might fall out: NR (19%)  Partner might not like it: NR (18%)  It is inserted into the vagina: NR (17%)  Need more information about the ring: NR (16%)  It is unfamiliar: NR (15%)  Need more information from health care provider: NR (13%)  G2:  Need more information from experienced user: NR (23%)  Concern about possible impact to kidneys: NR (22%)  Concern about sickness:  Potential side effects: NR (22%)  Need more information about the pill: NR (20%) Need more information from health care provider: NR (16%) Can easily forget to take the pill: NR (19%)  Dislike taking pills: NR (15%)  Among full sample:  Product preference, N (%):  G1*:  Clear preference: NR (31%)  Selected when asked to pick between two options: NR (5%)  Would use if pill not available: NR (59%)  G2:  Clear preference: NR (57%)  Selected when asked to pick between two options: NR (7%)  Would use if ring not available: NR (71%) |

Table 2c. Risk of Bias (Observational Studies)

| **Author;**  **Year** | **Potential for Confounding** | **Selection Bias** | **Bias in classification of intervention** | **Bias due to deviations from intended interventions** | **Bias due to missing data** | **Bias in measurement of outcomes** | **Bias in selection of reported result** | **Overall risk of bias** |
| --- | --- | --- | --- | --- | --- | --- | --- | --- |
|  | *Low/High/Unclear* | *Low/High/Unclear* | *Low/High/Unclear* | *Low/High/Unclear* | *Low/High/Unclear* | *Low/High/Unclear* | *Low/High/Unclear* | *Low/High/Unclear* |
| Browne;  2018 | Unclear | Unclear | NA | NA | Unclear | Unclear | Unclear | Unclear |
| RamaRao 2015, Ishaku 2015 | Unclear | Low | Low | Unclear | Low | Unclear | Unclear | Unclear |
| Routes 2 Results;  2017 | Low | Unclear | NA | NA | Unclear | Low | Low | Unclear |

Table 2d. Risk of Bias (Uncontrolled Observational Studies)

| **Author; Year** | **Clear criteria for inclusion in the study?** | **Consecutive inclusion of patients?** | **Analyses have complete inclusion of participants (i.e., loss to follow-up)?** | **Included participants comparable?** | **All participants validly selected consistently, reliable?** | **Outcomes assessed using valid measures in a consistent, reliable way?** | **Follow-up long enough for the outcome to occur?** | **If done, statistical analyses used appropria-tely?** | **Clear reporting of participants' demographic information?** | **Clear reporting of participants' clinical information?** | **Participants described with sufficient details for others to replicate or clinicians to make inferences?** | **Rating** | **Comments** |
| --- | --- | --- | --- | --- | --- | --- | --- | --- | --- | --- | --- | --- | --- |
| Merkatz;  2018 | Unclear | Unclear | Unclear | Unclear | Low | Unclear | Unclear | NA | No | No | No | Unclear |  |
| Nassuuna 2018, Ndagire 2018 | Unclear | Unclear | Unclear | Unclear | Unclear | Unclear | Unclear | NA | No | No | No | Unclear |  |
| Ipsos 2014, Ipsos NR | Unclear | Unclear | Unclear | Unclear | Unclear | Unclear | NA | NA | No | No | No | Unclear |  |
| Plascencia-Nieto,  2016 | Clear | Unclear | Unclear | Unclear | Unclear | Unclear | Unclear | Unclear | Unclear | Unclear | Unclear | Unclear |  |
| Prilepskaya,  2012 | Clear | Unclear | Unclear | Unclear | Unclear | Unclear | Unclear | Unclear | Unclear | Unclear | Unclear | Unclear |  |
| Quaife 2016, Quaife 2017 | Unclear | Unclear | Unclear | Unclear | Unclear | Unclear | Unclear | Unclear | Unclear | Unclear | Unclear | Unclear |  |
| Vijayaletchumi  2012, Siraj  2015 | Unclear | Unclear | Unclear | Unclear | Unclear | Unclear | Unclear | Unclear | Unclear | Unclear | Unclear | Unclear |  |

1. All unpublished manuscripts identified through the grey literature search have since been published. [↑](#endnote-ref-2)
2. Married or live with partner [↑](#endnote-ref-3)
3. All unpublished manuscripts identified through the grey literature search have since been published. [↑](#endnote-ref-4)
4. General ring worry=Very or somewhat worried about wearing a vaginal ring every day for at least a year (vs. not-at-all worried). [↑](#endnote-ref-5)
5. Adherence intervention included counseling and social support. [↑](#endnote-ref-6)
6. aRR=Adjusted relative risk [↑](#endnote-ref-7)
7. NS=Not statistically significant. [↑](#endnote-ref-8)
8. P-values from mixed-effect logistic regression models adjusted for country. [↑](#endnote-ref-9)
9. Selected results presented. Other logistic regression models were not statistically significant for any product: Completed secondary school, Earns income, Never/rarely attends religious service, Has place for privacy in home, Food worry past 4 weeks, Worried about HIV, Any intravaginal practices past 3 months. [↑](#endnote-ref-10)
10. This study also produced a peer-reviewed manuscript included in the acceptability tables. The outcomes reported here are additional to those reported in the manuscript. [↑](#endnote-ref-11)
11. Described by authors as cross-sectional study, although included intervention (VR) and two months of follow-up [↑](#endnote-ref-12)
12. DCE=Discrete choice experiment [↑](#endnote-ref-13)
13. FSW=Female sex workers [↑](#endnote-ref-14)
14. NS=Not statistically significant [↑](#endnote-ref-15)
15. Discontinued use before end of 2 month trial [↑](#endnote-ref-16)
16. Reminder strategies to replace the VR on time included: person centered counselling after administration of adherence questionnaires, sharing of DVR residual results, diary card training, and participant meetings conducted twice monthly, courtesy calls, home visits and interim clinic visits for participants with high residual levels. [↑](#endnote-ref-17)
17. FSW=Female sex workers [↑](#endnote-ref-18)
18. PWR=Preference weight ratio [↑](#endnote-ref-19)
